# A Randomised Controlled Trial of Therapist-Assisted, Internet-Delivered Cognitive Behavior Therapy for Women with Maternal Depression

**DOI:** 10.1371/journal.pone.0149186

**Published:** 2016-03-01

**Authors:** Nicole E. Pugh, Heather D. Hadjistavropoulos, Dale Dirkse

**Affiliations:** Department of Psychology, University of Regina, Regina, SK, Canada; University of Pennsylvania, UNITED STATES

## Abstract

Postpartum depression impacts up to 15% of Canadian women following childbirth. Remarkably, many women suffering from this disorder do not receive appropriate treatment. The aim of this study was to conduct a parallel-group randomized controlled trial to determine the efficacy of Therapist-Assisted Internet-delivered Cognitive Behavior Therapy (TA-ICBT) for the treatment of postpartum depression. This study was registered with the International Standard Randomized Controlled Trials (ISRCTN: 85456371) and received funding from Canadian Institutes of Health Research (#101526) and the Saskatchewan Health Research Foundation. Fifty women who gave birth to an infant in the past year, who scored above 10 on the Edinburgh Postnatal Depression Scale (EPDS), and who resided in Saskatchewan, Canada were eligible to participate. Participants were randomly assigned to receive either TA-ICBT (*n* = 25) or waitlist control (*n* = 25). The efficacy of the treatment was investigated at baseline and at seven- to 10-week follow-up. TA-ICBT participants were also contacted four-weeks following treatment completion. Symptoms of postpartum depression decreased more for participants in the TA-ICBT group (average reduction of 6.24 points on the EPDS; *n* = 21 included in analyses) compared to those participants in the waitlist control group (average reduction of 2.42 points on the EPDS; *n* = 20 included in analyses), and these results were clinically significant and maintained at four-week follow-up. TA-ICBT participants demonstrated a reduction in postnatal anxiety, general stress, and parental distress, and an increase in psychological and environmental quality of life when compared to the waitlist control participants. Study implications, limitations, and future research directions are discussed.

**Trial Registration:**

Controlled-Trials.com ISRCTN85456371

## Introduction

Postpartum depression (PPD) impacts approximately 8 to 15% of women within the first year of childbirth [1]. The disorder affects a woman’s daily functioning, ability to care for her infant, the mother-infant bond, and overall quality of life [2]. PPD may also result in short- and long-term developmental consequences for the infant [3]. While there is growing evidence in support of psychotherapy for PPD [4], participation rates in treatments are extremely low [5]. Stigma associated with receiving mental health treatment, difficulty arranging childcare, transportation challenges, and time and financial constraints are factors that contribute to the under-treatment of PPD [6].

### Internet-Delivered Therapy

Internet delivery systems could overcome many of these face-to-face treatment barriers. Receiving treatment from the comfort and convenience of a woman’s home would reduce mobility and childcare concerns. Relatively anonymous Internet-delivered therapy may circumvent women’s concerns of stigma [7]. Further, evidence is accumulating to suggest that Internet-delivered treatments are as efficacious and more cost effective in terms of the provider’s time and resources as compared to face-to-face treatments [8].

Ample empirical evidence supports the efficacy of Internet-delivered Cognitive Behavior Therapy (ICBT) in the treatment of major depression and sub-threshold depression. Particularly with therapist-assistance, ICBT is an effective method for reducing depressive symptoms when compared to treatment as usual (TAU), control groups, and treatment from a general practitioner [9–11]. Meta-analytic data from 21 studies indicate that effects obtained by supported Internet-delivered therapy for major and sub-threshold depression is analogous to those obtained in face-to-face therapy [9]. Moreover, supported Internet-delivered interventions evidence both greater program adherence [12] and stronger effects than self-directed interventions [13], with more frequent support resulting in greater symptom change [14].

### Internet-Delivered Therapy for Postpartum Depression

Preliminary research suggests that Internet-delivered treatment is efficacious for women in the postnatal period. In one study, researchers tested the efficacy of an Internet-delivered Behavioral Activation (iBA) program compared with TAU for PPD [15]. The 11-session program consisted of psychoeducation and behavioral exercises. Although the iBA program was self-directed, it included access to an Internet chat room that was moderated by parent supporters and supervised by specialist health visitors. Findings from 910 women with PPD indicated that participants in the iBA group reported greater reductions in depressive symptoms compared to the TAU group; attrition rates were high, however, with less than one-third of the participants completing the program. In an effort to improve treatment understanding and adherence rates, these same researchers recently created an 11-module iBA program and added a guided support component (i.e., telephone calls; [16]). Again, attrition rates were high. Only 5% of the women completed more than eight modules, with less than 2% completing all iBA modules. Women who reported lower perceived support, who were working or studying, and who reported lower socio-economic status completed fewer modules.

Another Internet-delivered intervention, Mom-Net, provided Therapist-Assisted ICBT (TA-ICBT) to economically disadvantaged mothers of young children [17]. Consisting of eight modules, Mom-Net included cognitive behavioral skills with adaptations relevant to mothers. Weekly phone calls were arranged between coaches and participants to assist with understanding and applying the materials. Women (*n* = 70) with elevated levels of depression, a child under five years of age, and attending a low-income program were randomly assigned to TA-ICBT or TAU. When compared to the TAU participants, Mom-Net participants reported improvements on measures of depression, parental satisfaction and efficacy, as well as a reduction on observational indices of harsh parenting behavior [17]. For the Mom-Net intervention, there was less attrition, with nearly two-thirds of women completing all Internet-delivered modules [17].

While preliminary research supports the efficacy of TA-ICBT for women of young children [17] and Internet-delivered behavioral intervention for PPD [15,16], researchers have not yet determined whether ICBT is an efficacious modality for treating sub-threshold and clinical PPD within the first post-natal year. Prior to implementation of TA-ICBT within community settings, it is important to ensure that the approach is more efficacious than symptom reduction over time as PPD is often a spontaneously remitting condition [18]. The purpose of the present investigation was to explore the efficacy of a specialized TA-ICBT program for women with symptoms of sub-threshold and clinical PPD. It was hypothesized that TA-ICBT participants would demonstrate greater symptom improvement than participants who received the delayed intervention on primary and secondary outcome measures (i.e., PPD, postnatal anxiety, general stress, parental stress, quality of life). These changes were expected to be clinically significant and maintained at follow-up.

## Materials and Methods

### Participants

Using the GPower program [19], to achieve power at 80% (alpha = 0.05), *n* = 23 participants per group were required to detect a medium effect size for a mixed model ANOVA. A medium effect size is consistent with previous meta-analyses of therapist-assisted ICBT [11,13].

A total of 50 participants were recruited to account for participant attrition. Participant recruitment and follow-up occurred between March, 2012 and February, 2013. Recruitment efforts included notifying physicians, perinatal nurses, and the public through the use of posters, information cards, Internet advertisements, and media interviews. Participant recruitment concluded when data collection was complete. Eligibility criteria were: (a) 18 years of age or older; (b) gave birth to an infant within the past year; (c) residing in Saskatchewan; (d) self-reported access to and comfort using a computer and the Internet; (e) score of ≥ 10 on the Edinburgh Postnatal Depression Scale (EPDS [20]); (f) consent to notify a physician of their participation; (g) not receiving other psychotherapy; (h) if taking medication, stable dose for more than a month; and (i) no past or present psychotic mental illness (schizophrenia), bipolar disorder, or current suicide plan or intent. S1 Table provides demographic details for the participants in the study. As displayed in S1 File, a total of 56 adult women responded to recruitment efforts. Of these, six participants were considered ineligible for reasons including: (1) they did not meet PPD symptom inclusion criteria, *n* = 2; (2) they declined to participate, *n* = 1; and (3) they recently had a change in their medication, *n =* 3.

### S1 File Flow of process for research participants

Participants who were interested in taking part in the study contacted the researcher. Participants were informed of the need to conduct an initial telephone screening interview to determine eligibility for the study. Verbal informed consent for the telephone screening was obtained at this stage and documented on each participant’s file. Participants then completed the telephone screening. Following this initial verbal consent and telephone screening, eligible participants then completed an electronic informed consent form followed by the online questionnaire battery. Participants were not paid for their participation in the study. The informed consent process was approved by the research ethics boards of the institutions involved. Verbal and electronic consent were deemed sufficient for participation. Ethics approval was granted by the Universities of Regina and Saskatchewan and the Regina Qu’Appelle Health Region in March, 2012. The trial was registered with the International Standard Randomized Controlled Trials (ISRCTN: 85456371) in June 2013. There was a delay in registering the trial as we were made aware of the benefits of registering the trial after data collection had begun. No changes were made to the study protocol between the start of the trial and the time of registration. The authors confirm that all ongoing and related trials for this intervention are registered. The project was partially supported by funding provided by the Canadian Institutes of Health Research (#101526) and the Saskatchewan Health Research Foundation.

### Procedure

#### Screening

Initial assessment included a telephone screen using the Edinburgh Postnatal Depression Scale (EPDS [20]) along with collection of background information including age of infant, computer and Internet access, and any current psychological or pharmacological treatment. Elevated PPD symptomatology was operationalized as a score of ≥ 10 on the EPDS. A threshold of ≥ 10 is commonly used to screen for subclinical PPD to increase sensitivity, and has been recommended for community settings to ensure that all potential cases of PPD are identified [21]. The mean participant EPDS score at pre-screening was 15.96 (*SD* = 3.89). Participants reporting sub-threshold PPD were included given that sub-clinical PPD symptoms are associated with parenting difficulties and adverse child outcome [22].

Following the initial assessment, participants then completed a telephone interview consisting of the administration of the MINI International Neuropsychiatric Interview (MINI [23]). The MINI confirmed sub-clinical and clinical symptoms of PPD and ruled out psychotic mental illnesses, bipolar disorder, alcohol or substance disorders, and those at elevated risk of suicide. Participants were also required to complete a battery of self-report measures administered over the Internet as described below.

#### Randomization

Eligible and willing participants were individually randomized (allocation 1:1) to either TA-ICBT/ Maternal Depression Online or WLC conditions. Participants were allocated in equal numbers to each intervention using blocked randomization. Random allocation was conducted via an Internet computer program by a researcher who was not involved with the research study. The researchers were blind to the randomization process and were notified through e-mail regarding participant allocation following the screening process. Following the notification, the primary researcher (N.E.P.) contacted the participants over the telephone and informed them of their assigned treatment group (i.e., TA-ICBT or WLC). Due to the nature of the conditions (i.e., TA-ICBT or WLC), it was not possible for the study therapists and participants to be blind to the treatment assignment.

#### Post-Intervention and Follow-Up Assessment

Post intervention/delay period measures were administered to all participants over a secure Internet website (T2; 7–10 weeks after T1). Once the T2 measures were completed by the WLC participants, they were contacted and offered TA-ICBT. Four-week follow-up measures were administered over the Internet for participants assigned to the TA-ICBT condition only (T3; 4 weeks after T2).

### Measures

#### Primary Assessment Measure

The EPDS [20] is a 10-item self-report measure that assesses emotional and cognitive symptoms of PPD. Each EPDS item is scored from 0 to 3 in relation to the past seven days. Total scores range from 0 to 30 with higher scores indicating more severe symptomology. The EPDS has demonstrated excellent psychometric properties [20], is sensitive to change over time [21], and the computerized version has been validated [24]. In the current study, the EPDS demonstrated acceptable internal consistency, α = .80.

#### Secondary Assessment Measures

The Depression Anxiety Stress Scale- Short Form [25] (DASS) is a 21-item measure assessing dysphoric mood, fear and autonomic arousal, and general nervousness and agitation. With reference to the past week, participants respond to items on a 4-point Likert scale. The computerized version of the DASS has been validated [26] and the measure has been assessed in a population of postnatal women with and without PPD [27]. In the current study, the DASS-21 demonstrated acceptable internal consistency for each subscale: Depression α = .81; Anxiety α = .77; and Stress α = .78.

The Parenting Stress Index-Short Form (PSI-SF [28]) comprises three subscales assessing parental distress, parent-child dysfunctional interaction, and perception of a difficult child. Each subscale consists of 12 items rated from 1 (*strongly disagree*) to 5 (*strongly agree*). The PSI-SF has demonstrated excellent psychometric properties and is appropriate for use with parents of children as young as one month [28]. In the current study, the PSI-SF exhibited acceptable internal reliability: Parental Distress α = .77; Parent-Child Dysfunction α = .87; and Difficult Child α = .90.

The World Health Organization Quality of Life Assessment BREF (WHOQOL-BREF [29]) is a 26-item measure assessing quality of life in four domains: Physical health (e.g., sleep, pain); Psychological health (e.g., self-esteem, concentration); Social relationships (e.g., support, personal relationships); and Environment (e.g., physical safety, financial resources). Participants respond on a 5-point Likert scale ranging from 1 (*not at all*) to 5 (*an extreme amount*). The computerized version of the WHOQOL-BREF has demonstrated excellent psychometric properties [30] and the measure has been validated on women with and without PPD [31]. In the current study, the four WHOQOL-BREF domains demonstrated acceptable internal consistency: Physical α = .65; Psychological α = .67; Social α = .65; and Environment α = .87.

#### Treatment Relevant Outcome Measures

The Therapeutic Alliance Questionnaire (TAQ) consists of 17 items assessing the perceived helpfulness of the ICBT therapeutic relationship and has been used in multiple TA-ICBT studies [32]. The TAQ was adapted from the Helping Alliance Questionnaire (HAQ-II [33]). Statements are rated from 1 (*strongly disagree*) to 6 (*strongly agree)* with higher scores indicating the participant perceived the ICBT relationship as helpful. The HAQ-II has demonstrated acceptable psychometric properties [34], but the psychometric properties of the TAQ have not been reported. In the current study, the TAQ demonstrated excellent internal consistency, α = .94.

Treatment satisfaction was assessed with two questions: “How much did you like the treatment program?”, and “How much did you enjoy communicating with your Internet therapist?” Each item was rated on a 0 (*not at all*) to 7 (*very much so*) Likert scale. These questions were adapted from the Treatment Satisfaction Questionnaire-Modified (TSQ [35]). In this study, the responses to the two items were summed to create a total TSQ score. The responses to the two items were highly correlated, *r* = 0.77.

The Credibility/Expectancy Questionnaire (CEQ [36]) assessed perception of treatment credibility with four items rated on a scale ranging from 1 (*not at all*) to 9 (*very much*), while treatment expectancy was measured with two items rated using a 0 to 100% scale. Psychometric properties are strong and factor analysis has confirmed the subscales of the CEQ [36].

### Treatment Conditions

#### Waitlist Control

Participants randomized to the WLC condition were provided with an information pamphlet that included psycho-education on PPD and websites to access provincial mental health support services. They were requested to inform the researchers of any treatment they received during the wait period.

#### TA-ICBT for PPD

Maternal Depression Online was adapted from a TA-ICBT program for depression offered through the Online Therapy Unit for Service Education and Research (www.onlinetherapyuser.ca [37]), located in Saskatchewan, Canada. The original program content was licensed from Swinburne University of Technology National eTherapy Centre [38]. Maternal Depression Online maintained much of the original program’s content, but incorporated adaptations relevant to mothers of young infants based on Milgrom et al.’s treatment for PPD [39]. Given that treatment studies for in-person CBT for PPD typically range from six to eight sessions [40] and TA-ICBT studies for major depression often range from five to eight modules [13], the depression program was shortened from 12 modules to seven modules. Material that is not traditionally part of CBT was removed such as information on nutrition and mindfulness. Each module included a range of media (e.g., text, graphics, animation, audio, video) given research to suggest that multimedia options enhance the effectiveness of Internet-delivered treatment and are preferred over static treatments [41].

Maternal Depression Online was designed so that each page of the program was viewed prior to proceeding to the next page. Check-in questions focusing on the module content were presented at the beginning of each module while homework exercises were assigned at the end of each module. Participants were encouraged to progress at a pace of one module per week although more time was often taken. Most TA-ICBT and WLC participants completed T2 measures at a standard 10-week follow-up time. Measures were administered earlier, however, if the participants withdrew from the program (*n* = 3) or completed seven modules in a shorter period of time (*n* = 6).

Participants were provided with a username and password that allowed them to access the site and message their therapist over a secure private messaging system. An industry-standard encryption algorithm was used for all data, emails, and check-in questions, and stored on a dedicated university server [37].

The Internet therapists included two doctoral students in Clinical Psychology (including co-author N.E.P.) who were supervised by a registered psychologist and expert in TA-ICBT (co-author H.D.H.). Internet therapists completed a training workshop in the provision of TA-ICBT [42]. Therapists emailed their assigned participant on a set day each week to provide support and encouragement, and to answer questions. E-mails took a variable length of time to compose depending on the information submitted to the therapist with the average email taking 15 to 20 minutes to compose. While the e-mail content overlapped substantially with the treatment material, different components were emphasized and individualized depending on the problems presented by the participant. The e-mails were not pre-prepared. If a participant reported significant distress or failed to log into the program for over seven days, the therapist telephoned the participant.

### Statistical Analysis

All outcome data were analyzed on an intention-to-treat basis such that the analyses included every participant who was randomized according to randomized treatment assignment. Between-group intervention effects on the EPDS from baseline (screening) to T2 (post-intervention/wait-time) were compared using longitudinal mixed effects models [43]. The longitudinal mixed model uses all data on each subject, is unaffected by randomly missing data, and it can flexibly model time affects. The more familiar mixed model ANOVA (condition as the between subject factor; time as the within subject factor) is a special case of a longitudinal mixed effect model. Random intercept models were computed and a maximum likelihood method with covariance type (based on the variance components) was employed to provide the estimates. Chi-square analysis will determine if the difference between the two conditions is statistical significant.

Clinically significant change on the EDPS was calculated using procedures outlined by Matthey [44] in their study of 181 women with PPD. Matthey followed Jacobson and Truax’s [45] calculations to determine the magnitude of change on the EPDS required to be considered clinically significant. Participants whose EPDS score of more than 10 at baseline, decreased by at least four points were classified as *recovered* based on calculations of Matthey’s Reliable Change Index (RCI) for the EPDS. Those who demonstrated a decrease in their EPDS score of four or more points but still scored 10 or more after treatment were classified as *improved but not recovered*. Those participants who showed a four point increase in their EPDS score after treatment were classified as *deteriorated*. Finally, participants whose pre-post difference score was less than four points were classified as *no change* regardless of whether or not their post-treatment scores fell below the cut-off score of ten. The procedure and recommendations outlined by Matthey [44] have been utilized in other Internet therapy research (e.g., [16])

Multiple regression analyses were used to determine if group differences (TA-ICBT versus WLC) predicted secondary outcome measure scores (i.e., DASS, PSI, WHO-QOL subscales) when controlling for pre-treatment scores on each respective secondary outcome measures. The effects of missing follow-up data were explored by imputing missing data using chained equations [46, 47] that impute data for all relevant variables. All multiple regressions were analyzed twice—once with the original data set and once with the imputed data set. Unstandardized coefficients were used as a measure of effect size to assess the proportion of variance associated with or accounted for by each of the regressions. The results of the two regression analyses were compared to determine if the missing data impacted the results.

A within-subjects analysis for participants receiving TA-ICBT was conducted comparing four-week follow-up (T3) EPDS scores with post-treatment (T2) and baseline EPDS scores (screening). Finally, descriptive statistics are reported for program engagement, satisfaction, and therapeutic alliance reported by the TA-ICBT group (i.e., TSQ, TAQ, CEQ).

## Results

### Preliminary Analyses

The TA-ICBT and WLC groups did not differ with respect to demographic characteristics (see S1 Table). Descriptive statistics for primary and secondary outcome measures administered at T1 and T2 are presented in S2 Table. There were no differences between completers and non-completers on the baseline EPDS: *t*(48) = 1.87, *p* = .067, *d* = .54. The distribution of the EPDS was normal and similar variance was revealed between the two groups. Attrition rates for TA-ICBT and WLC groups were 16% (*n* = 4) and 12.5% (*n* = 3), respectively between T1 and T2. Attrition rates for the TA-ICBT group between T2 and T3 was 28.5% (*n* = 5).

### Treatment Received Reported by WLC Group

WLC participants reported receiving various support/care during the waiting period. Close to 58% reported regular contact with their family physician, 32% sought psychotherapy for maternal depression, and 25% of participants reported using psychotropic medication.

### Primary Outcome Measure

Results from the longitudinal mixed model analysis indicated no effect for condition on EPDS scores, *F*(1, 48.07) = .35, *p* = .56, a statistically significant change in EPDS scores over time, *F(*1,20.99) = 16.23, *p* = .001, but most importantly and consistent with our primary hypothesis, a condition by time interaction, *F*(1, 11.82) = 5.15, *p* = .02. Following the criteria outlined by Matthey [44], approximately 20% of participants receiving TA-ICBT were classified as improved, while over 62% were recovered. On the other hand, while 38% of the participants allocated to WLC condition were classified as recovered, over 50% exhibited no reliable change. Chi-square analysis showed the differences between the two conditions approached statistical significance: χ^2^(1) = 2.93, *p* = .08, Cramer’s *V* = .026.

### Secondary Outcome Measures

In order to determine whether the treatment influenced participants’ stress, anxiety, depression, parental stress, and quality of life, scores on these variables at T2 were the outcome variables in a series of multiple regressions. The predictor variables were condition (TA-ICBT or WLC) and scores on the secondary outcome variables at T2. The results for these analyses are presented in S3 Table. When compared to the WLC, the TA-ICBT at T2 (controlling for scores at T1) produced lower stress (β = -.41), lower parental stress (β = -.25) and distress (β = -.41), and better psychological health (β = .34) and environmental quality of life (β = .31). A comparison of the results from the original data set to the pooled results indicated no statistically significant differences.

### Follow-Up for the TA-ICBT Condition

We conducted within-subjects analyses comparing follow-up scores (T3) with post-treatment scores (T2) for participants in the TA-ICBT condition. The EPDS collected at follow-up had statistically significant and large gains compared with post-treatment scores, *t*(14) = 4.13, *p* < .01, *d* = 1.10, indicating that intervention effects not only were maintained, but also that the TA-ICBT participants continued to improve.

### Program Engagement and Satisfaction

S4 Table presents the descriptive statistics for program engagement. TA-ICBT participants completed on average 5.92 of the seven modules (60% of the participants completed all seven modules). Less than 18% of participants dropped out before the fourth module; the greatest attrition occurred during the 5^th^ of 7 modules (11.8%). The website was extensively used by participants as indicated by the mean number of program visits (*M* = 26.88; *SD* = 11.63), and the number of emails sent (*M* = 5.4; *SD* = 4.15) and received by the participants (*M* = 10.52; *SD* = 3.95). Over 80% of the participants reported that they liked the overall program and enjoyed communicating with their therapist (TSQ). Participants also reported a high level of therapeutic alliance, giving a rating of 86.42% (TSQ).

## Discussion

PPD is an undertreated condition that may result in both short and long-term consequences for the infant [3]. The results of this study suggest that a therapist-assisted Internet-delivered intervention is efficacious for women with PPD. While we were not able to compare the findings to an active treatment condition, when compared to WLC TA-ICBT not only reduced symptoms of PPD, but also reduced stress, parental distress and improved domains of quality of life. Reductions in PPD were clinically significant and symptom reduction on the EPDS was maintained at follow-up. Our findings were in accordance with evidence to support the efficacy of Internet-delivered behavioral activation for PPD [15,16], TA-ICBT for economically disadvantaged mothers of young children [17], and face-to-face CBT for PPD [4]. Further, approximately 20% of TA-ICBT participants were considered improved, while over 62% were considered recovered. This high rate of symptom recovery was promising given not only the morbidity and consequences associated with PPD for the mother, but also the pervasive effects PPD has on the family. Moreover, resulting from the multiple face-to-face treatment barriers reported for women with PPD, this is the first study providing evidence that TA-ICBT is an efficacious treatment for women struggling with PPD and this novel approach may overcome face-to-face treatment barriers [6].

In addition to the efficacy of TA-ICBT, our results indicated significant program adherence, with participants completing, on average, 5.92 of the seven modules (60% of completed the entire program). Participants not only completed weekly check-ins with each module, but they also messaged their therapist on average five times. Therapists, in turn, provided considerable support to participants sending, on average, eleven messages over the course of treatment. A strong therapeutic alliance and high satisfaction with the overall program was also reported. Findings are consistent with a qualitative investigation that explored participants’ perceptions of this program [48]; the majority of women expressed that the TA-ICBT program was flexible, accessible, convenient, and afforded them increased feelings of anonymity and privacy. The degree of engagement was promising given past research findings of lower participant involvement in Internet-delivered treatment. O’Mahen et al. [15], for instance reported only a 39% completion rate for an Internet-delivered behavioral activation intervention for PPD that included weekly automated email reminders and no therapist assistance. On the other hand, for a TA-ICBT program offered to mothers of young children that included weekly telephone calls from a therapist, similar to the present study, 63% of participants completed all treatment modules [17]. Collectively, these findings are in accordance with a meta-analysis suggesting that for the treatment of major depression, when compared with self-administered Internet–delivered interventions, some form of guidance is superior (i.e., TA-ICBT [13]). While weekly Internet therapeutic contact has been validated in a sample of participants diagnosed with panic disorder [32], research has yet to establish the ideal therapist-client contact frequency for women with PPD. Given the multiple demands and challenges of parenting, often coupled with low levels of social support [49], women with PPD may reap additional benefits from more frequent therapeutic contact.

We did not find any group differences for the secondary outcome measures relating to general depression, general anxiety, overall parental stress, and select domains of quality of life. Interestingly, group differences were revealed for depression when assessed with the EPDS, but not when measured with using the DASS-21. This may have been the result of the sensitivity of the DASS-21 to assess postnatal depression. Indeed, general depression measures often rely on somatic symptoms of depression, which overlap with physical symptoms associated with the postpartum period [50]. While statistically significant group differences were revealed with respect to parental distress, group differences were not identified for the other parental stress subscales. The Parental Stress Inventory may not have adequately tapped into the parental stress reported by women with PPD as the measure was originally validated on a sample of mothers of young children (mean age under four years) and a variety of questions focused on parental stress related to caring for an older child [28].

Given the dearth of literature in the area of TA-ICBT for PPD, future research directions are abundant. To begin, additional well-designed trials of TA-ICBT for PPD are required. It is suggested that these studies include a larger, more diverse sample, as well as longer-term follow-up times. Varying Internet therapeutic contact and styles could also be compared, such as weekly versus bi-weekly therapist contact or telephone versus email versus audio/video correspondence. Additionally, given the efficacy and participant satisfaction of TA-ICBT for PPD, future research exploring TA-ICBT for the treatment of other perinatal clinical disorders is warranted, such as a transdiagnostic program to concurrently address symptoms of perinatal anxiety and depression. The comparison of TA-ICBT to an active treatment condition such as face-to-face CBT as well as other Internet-delivered treatments (e.g., Internet behavioral activation) would also be beneficial, as not all participants responded favorably to TA-ICBT. An economic evaluation study comparing the cost-effectiveness and cost utility of TA-ICBT for PPD compared with face-to-face treatment is also warranted. Finally, future implementation research is required to determine whether different health care professionals are equally effective in delivering TA-ICBT for PPD and how this type of novel programming can be effectively implemented and utilized within community settings.

### Limitations

There were a number of limitations that should be considered. The sample utilized was relatively small and prohibited an examination of other factors. The results, however, are promising as the sample included participants with sub-threshold PPD. An additional limitation was the absence of an active control condition, which may have exaggerated our results. However, the study demonstrates considerable feasibility and acceptability of TA-ICBT for PPD. The limited sample with respect to demographic characteristics was an additional drawback. Moreover, given that more than half of the sample reported some university education, additional research utilizing a more heterogenous sample is required. A group comparison at follow-up was not conducted, which would have been informative given that PPD is often a spontaneously remitting condition [18] and, therefore, may improve over time irrespective of treatment. For ethical reasons, however, we did not unduly extend the waiting period for WLC participants. Additionally, depressive symptoms were assessed using self-report measures, which are known to produce false positives. While we conducted a clinical interview (i.e., MINI) to screen participants, a post-treatment clinical interview would have provided a more objective measure of changes in depressive symptoms. Finally, these findings are specifically related to one TA-ICBT program (i.e., Maternal Depression Online) delivered by Internet therapists who were trained graduate students in clinical psychology. A recent literature review suggests that ICBT outcomes do not differ when delivered by experienced versus inexperienced therapists [51]. It is possible that the structure of TA-ICBT (e.g., modules) results in less variation in the delivery of TA-ICBT than is observed in face-to-face therapy; therefore, similar outcomes may arise regardless of therapist experience [52]. Nevertheless, it is possible that outcomes could have been more impressive had a different TA-ICBT program been used. It would be valuable to conduct further research on which type of allied health provider will obtain the best outcomes with TA-ICBT for PPD and who is best positioned to deliver TA-ICBT if delivered in community settings.

## Conclusion

Despite some limitations to the research design, we demonstrated that TA-ICBT was an efficacious, well-utilized, and desirable intervention for women with PPD. When compared to a WLC condition, TA-ICBT appeared more efficacious at reducing symptoms of PPD, parental stress, and improved psychological and environmental quality of life. Further investigation for TA-ICBT for PPD is required, particularly comparing TA-ICBT to an active control condition, utilizing a larger and more heterogeneous sample, with longer-term follow-up. Additionally, an examination of the cost-effectiveness of TA-ICBT when delivered in community settings would also be desirable alongside the study of factors that constrain or enhance the implementation of TA-ICBT in community practice.

## Supporting Information

S1 CONSORT Checklist Consort List. (DOC)

S1 File Consort Fig. (TIFF)

S2 File Database. (SAV)

S1 Protocol Ethics Protocol. (DOCX)

S1 Table Demographic Characteristics by Group. (DOC)

S2 Table Means and Standard Deviations for Primary and Secondary Outcomes. (DOC)

S3 Table Regression Analyses of Secondary Outcome Variables. (DOC)

S4 Table Descriptive Statistics for Program Engagement in the TA-ICBT condition (*N* = 24). (DOC)
