# Supplementary figures and images for: A Randomised Controlled Trial of Therapist-Assisted, Internet-Delivered Cognitive Behavior Therapy for Women with Maternal Depression
Source: PLoS One. 2016 Mar 1;11(3):e0149186. doi: 10.1371/journal.pone.0149186 (PMC4773121; doi:10.1371/journal.pone.0149186)

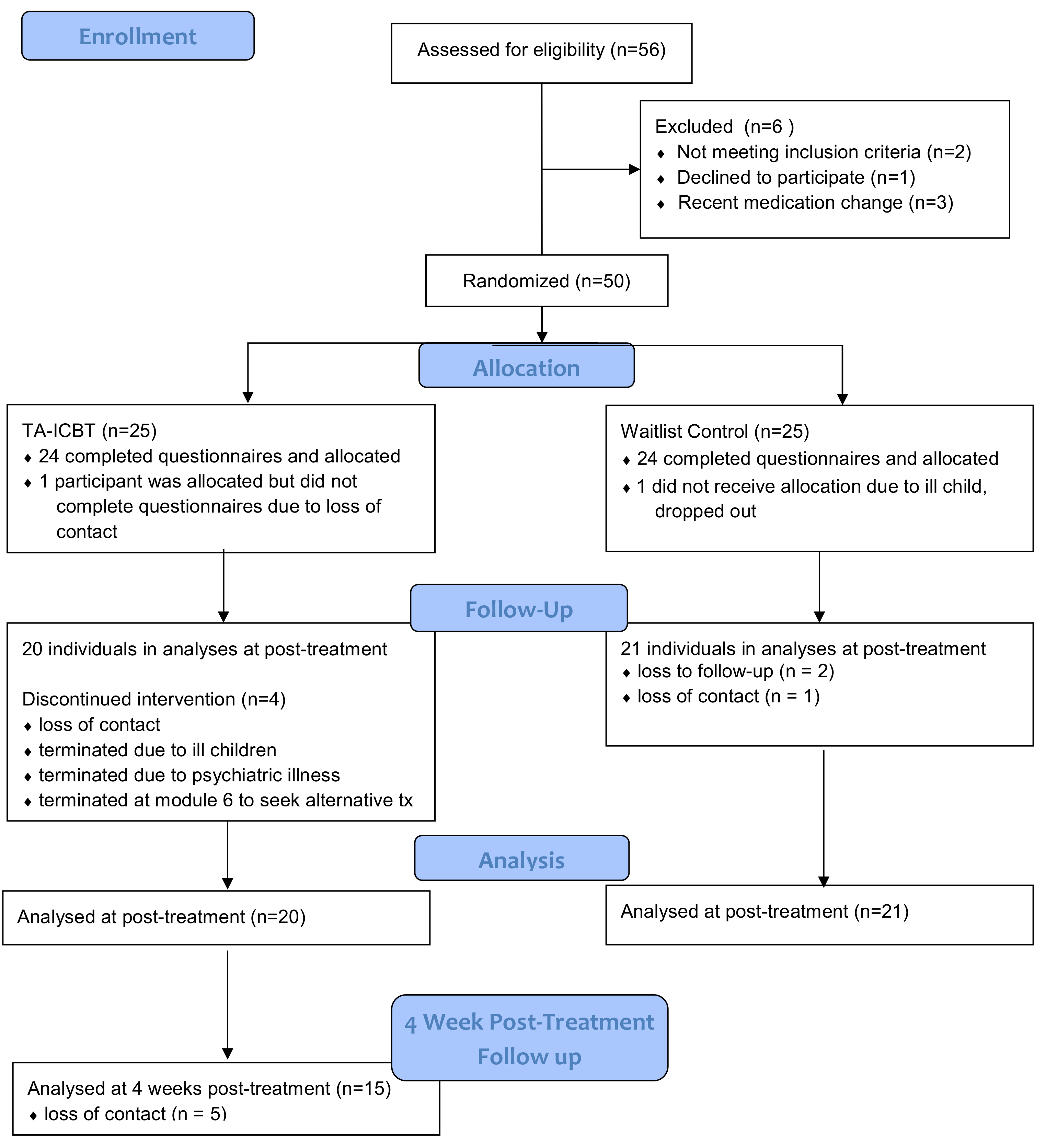

Supplement: S1 File — (TIFF) [file pone.0149186.s002.tiff]
