# Supplementary material for: A Randomised Controlled Trial of Therapist-Assisted, Internet-Delivered Cognitive Behavior Therapy for Women with Maternal Depression
Source: PLoS One. 2016 Mar 1;11(3):e0149186. doi: 10.1371/journal.pone.0149186 (PMC4773121; doi:10.1371/journal.pone.0149186)
Supplement: S1 Protocol — (DOCX) [file pone.0149186.s004.docx]

**UNIVERSITY OF REGINA**

**RESEARCH ETHICS BOARD**

**Application for Approval of Research Procedures**

**(last updated April 2010)**

# Section I: Identification and Purposes

1. Date: January 11^th^, 2012

Name of Applicant(s): Nicole Pugh

Department or Faculty: Psychology

Co-Applicants: N/A

Student # (if applicable): 200277350

Mailing Address (if different than Department):

Telephone #: (306) 581-2468

E-mail: nicky.e.pugh@gmail.com

Title of Research: A randomized controlled trial of a therapist-assisted internet cognitive behaviour therapy program for women with postpartum depression.

2. Students please provide:

Student level Graduate Undergraduate

Name of supervisor: Dr. Heather Hadjistavropoulos Department: Psychology

3. Signatures and Acknowledgement:

Your signature(s) below acknowledges that:

- the information in this application is correct to the best of your knowledge

- you will notify the REB of any changes or amendments to this application

- contact with human subjects in the proposed research will not commence until ethical approval is obtained

- all members of the research team are aware of, and adhere to, University of Regina regulations and policies for conducting research, including the Tri-Council Policy Statement (TCPS).

Signature of Applicant(s) Signature of Advisor or Instructor

_______________________________ ________________________________

____________________

Date

**Reminder**: Please attach a copy of your recruitment letter or poster, consent form, questionnaire, interview questions, etc.

4. Provide an overview of the main features and variables of the research problem. Include a brief review of the relevant literature, a statement that describes the significance and potential benefits /of the study, your hypotheses (if applicable), a brief description of your measures and some information about your design and analytic approach (e.g., "narrative data will be analyzed through grounded theory methodology"; "a 2 x 2 Multivariate Analysis of Variance will be employed for the data analysis").Maximum one page.

***Introduction***. Postpartum depression (PPD) and sub-clinical PPD affect approximately 8 to 15% of Canadian women following childbirth (Chalmers et al., 2008). The disorder is debilitating and impacts a woman’s quality of life and her ability to care for and connect with her infant. PPD is also related to short- and long-term consequences for the infant (Tronick & Reck, 2009). Unfortunately, PPD is undertreated for reasons including lack of access to providers and limited mobility. Using the internet to deliver therapy is an innovative way to improve access to psychological treatment. Evidence has suggested that internet cognitive behavior therapy (ICBT) results in significantly larger effect sizes compared to all other online interventions (Barak et al., 2008) and that therapist-assisted ICBT (TAICBT) is comparable to in-person CBT (Spek, 2007).While there is evidence to support the efficacy of TAICBT for the treatment of major depression (Cuijpers et al., 2010), to date, researchers have yet to determine whether this modality is efficacious for the treatment of PPD.

***Purpose and Hypotheses****.* The proposed study will determine whether TAICBT is more efficacious than a waitlist control (WLC) condition in reducing depressive symptom severity, anxiety, general stress, parental stress, and for improving quality of life for women with PPD. It is hypothesized that TAICBT participants will demonstrate significantly greater symptom and clinical improvement from pre- to post-treatment on all outcome measures. It is also expected that symptom improvements will be maintained four weeks following the conclusion of treatment. Given that the TAICBT has not been examined in PPD populations, treatment expectancy, satisfaction, and therapeutic alliance will also be investigated. It is hypothesized that: (a)TAICBT participants will report satisfaction with the treatment,(b) significant associations will be revealed between post-treatment therapeutic alliance ratings and symptom changes scores, and (c) treatment expectancy and satisfaction will be moderately correlated with symptom change scores on all outcome measures. To further understand the participants’ experience with TAICBT, ten open-ended questions will be asked.

***Method****.* Using a randomized controlled design, 58 women will be randomized to receive either TAICBT treatment or assigned to a 7-10 week WLC. Participants assigned to the WLC will receive a pamphlet that includes information on information on PPD and websites for support services offered in Saskatchewan. The intervention will consist of a seven-module TAICBT program adapted for women afflicted with PPD. Each TAICBT participant will be assigned to a therapist who they can correspond with weekly via email. Potential participants will be screened using the **Edinburgh Postnatal Depression Scale** (EPDS; Cox et al., 1987), modules from the **MINI International Neuropsychiatric Interview** (Sheehan et al., 1998), as well as standard screening questions. If deemed eligible, the following additional outcome measures will be administered: (a)**Depression Anxiety Stress Scale- Short Form** (DASS; Lovibond & Lovibond, 1995) to measure levels of depression, stress, and anxiety; (b)**Parenting Stress Index- Short Form** (PSI-SF; Abidin, 1995) to assess stress in the parent-child system; (c) **World Health Organization Quality of Life – BREF** (WHOQOL-BREF; WHOQOL Group, 1998) to assess quality of life;(d) **Therapist Alliance Questionnaire** (TAQ; Kiropoulos et al., 2008, modified from Luborsky’s 1985 HAQ-II) to determine the degree to which participants perceive the relationship with their therapist as helpful; (e) **Treatment Satisfaction Questionnaire-Modified** (TSQ; Cox et al., 1994) to assess the client’s perceived satisfaction with treatment; and (f) **Credibility/Expectancy Questionnaire**(CEQ; Devilly & Borkovec, 2000) to measure credibility and treatment expectancy of the TAICBT treatment. The TAQ, TSQ, and CEQ will only be completed by those participants receiving TAICBT treatment. All other measures will be completed by both groups of participants at pre-treatment and post-treatment (10 weeks after baseline). Following the 7-10 week wait, participants in the WLC group will be contacted, re-screened, and if still eligible, they will be offered the treatment. At this time, the TAICBT participants will be asked to respond to ten open-ended questions regarding their experiences with the program. To provide a longer-term follow-up of depressive symptoms, TAICBT participants will be contacted by telephone four weeks following treatment completion and administered the EPDS and the depression module of the MINI.

***Analysis****.* Means, standard deviations, and ranges of all scores for all pre- and post-treatment measures will be computed. Independent samples *t*-tests and chi-square tests will be conducted to assess for group differences in demographic data and baseline measures. Treatment efficacy will be determined using repeated measures mixed-model analysis of variances (ANOVAs). Eta square effect sizes will be computed for all outcome measures. The Jacobson and Truax (1991) method will be used to discern clinical significance, and remission and recovery rates will be reported for both groups. Mixed-model ANOVAs along with tests of simple effects and interaction contrasts will be computed to determine if the TAICBT group maintained the expected treatment gains. The open-ended questions will be analyzed using a grounded theory approach.

***Discussion.*** If found effective, the TAICBT program for PPD could benefit not only Saskatchewan mothers’ mental health and quality of life, but also their infants’ physical and psychological health and the family environment.

5. Researcher Qualifications: Describe any special training or qualifications you and/or the research team have - only in cases where the research involves special or vulnerable populations (e.g. children, incarcerated individuals, etc), distinct cultural groups, or the research is above minimal risk, otherwise this section may be omitted.

N/A

# Section II: Application Checklist

1. Do you consider that this project involves:

HIGH RISK to subjects

MORE THAN MINIMUM RISK to subjects

MINIMUM RISK to subjects

Researchers are advised that "Risk" is defined broadly to include not only threats to one's physical integrity or health but also temporary as well as permanent psychosocial consequences (e.g., experiencing negative mood for a brief period as a result of research participation, potential for violations of privacy, potential for upsetting a third party because of research participation).

If other than MINIMUM RISK, please explain and submit the full research proposal (e.g., grant application, thesis proposal) or, if a full proposal is not available, contact the REB Chair.

We have classified this research as minimum risk. Clients will either be assigned to a waiting list condition or an intervention condition. In the waiting list condition, clients are free to receive usual care. Furthermore, we will be providing them a pamphlet that has been designed to assist women with PPD with accessing care in Saskatchewan. Clients who are assigned to the intervention condition, will be receiving treatment that has been found to be helpful for clients who have depression, and, therefore the program is anticipated to actually reduce risk. Following the 10-week wait time, participants in the waitlist group will be re-screened, and if they still meet eligibility criteria for the study, they will be offered the opportunity to receive TAICBT treatment.

Clients who receive TAICBT, will be communicating with their therapists over the onlinetherapyuser website. There are multiple safeguards in place to ensure that information is protected and kept securely (see below).

The REB reserves the right to request a copy of the full research proposal for any project that is assessed (by any one of the reviewers of the application) as involving more than minimum risk.

2 What are the sources of funding (if any) for the proposed research?

Nicole Pugh is funded by the Canadian Institutes of Health Research’s Regional Partnerships Program (CIHR-RPP) and the Saskatchewan Health Research Foundation. Her supervisor Dr. Heather Hadjistavropoulos and the Online Therapy Unit are funded by the Canadian Institutes of Health Research and the Saskatchewan Health Research Foundation.

3a. Will the researcher(s), members of the research team and/or their immediate family members receive any personal benefit (e.g. financial benefit such as remuneration, intellectual property rights, rights of employments, consultancies, board membership, share ownership, stock options, etc) as a result of or in connection to this study?

Yes

No

If Yes, please describe the benefits.

3b. Is there any compensation for this study that is affected by the study outcome?

Yes

No

If Yes, please explain.

3c. Do you think that the research findings from this project might be commercially valuable to others (e.g. the researchers’ employers, the project sponsors)?

Yes

No

If Yes, please explain.

Dr. Hadjistavroplous licensed the online therapy programs for depression, anxiety, and panic disorder from the National eTherapy Centre in Australia. The license included the option to adapt the online program into revised treatment programs. The findings of this project will not be commercially valuable.

3d. If yes to any of the above, please describe the arrangement and discuss the implications of a potential conflict of interest. Please describe how the conflict is being managed and what additional protections have been put in place to protect the study participants. This should be included in the Letter of Information to participants.

*N/A*

4. Where relevant, please explain any pre-existing relationship between the researcher(s) and the researched (e.g. instructor-student, manager-employee, co-workers, etc). Please pay special attention to relationships in which there may be a power differential. Describe any safeguards and procedures to prevent possible undue influence, coercion or inducement.

There will be no pre-existing relationships between the researcher and any participants enrolled in this study.

5. Does your research project require approvals from other organizations such as school boards, aboriginal communities, local governments, etc? Please describe. What steps will you take (or have taken) to obtain these approvals?

N/A

6. How long do you expect your research project (contact with human subjects for data collection) to last?

Less than one year from the date of approval

More than one year from the date of approval (an annual renewal will be needed every year)

# Section III: Subjects

1. **Briefly describe the number and characteristics of participants required for the study, and how a potential sample of such participants will be identified.**

Participants will include approximately 58 women residing in Saskatchewan seeking TAICBT treatment for symptoms of PPD. Potential participants will respond to advertising for *Maternal Depression Online* or will be referred by healthcare providers. Next, participants will be screened through questions on the Online Therapy USER website and by telephone to determine if they are eligible for TAICBT services and whether this form of service is appropriate for their concerns.

A woman will be deemed eligible to participate if she is a Saskatchewan resident, 18 years or older, meets diagnostic criteria for major depression or sub-clinical depression, and has a child under 12 months of age. Diagnostic criteria for PPD and sub-clinical PPD will be determined using cut-off scores on the Edinburgh Postnatal Depression Scale (Cox et al., 1987; see Appendix C) and a structured clinical interview for psychological disorders (MINI; Sheehan et al., 1998; See Appendix E).Participants will also be required to have regular access to a computer and the internet and endorse familiarity and comfort with using them. Lastly, participants will need to provide a contact name and number of a physician or another medical contact as this contact will be notified of the client’s participation in the online treatment program (see Appendix N).

Participants will be excluded from the study if they do not meet the above criteria or if they are (a) currently receiving psychotherapeutic treatment elsewhere or in some other form; (b) meet criteria for current substance abuse or dependence (drugs or alcohol); (c) meet criteria for a current psychotic disorder or bipolar disorder, or severe symptoms of depression, including suicidal ideation; or (d) report having a serious medical condition that may interfere with treatment completion.

2. Describe the recruitment procedures. Who will approach potential participants (researcher, assistant or third party) and how (e.g. by phone, mail)?

The principal investigator will recruit participants from across Saskatchewan using several methods. In an effort to reach women in both rural and urban Saskatchewan, advertising will begin on internet websites including: www.skmaternalmentalhealth.ca, preventioninstitute.sk.ca, kinderbuzz.com, onestopkidshop.com, earlylearning.ca, and facebook.com (see Appendix A for a sample advertisement). In addition, postings will be placed on list serves and emailed to individuals around Saskatchewan. Recruitment efforts will also involve advertising and news stories related to the study, which will be published in newsletters and urban and rural newspapers (e.g., *The Leader Post*, *Star Phoenix, Yorkton This Week*). Advertisements will outline the details of the treatment study, will include the researchers’ name and contact information, and will provide a link to the website (http://www.onlinetherapyuser.ca/), where additional information and instructions on how to participate in the study can be found.

In person recruitment strategies will also be used. This will include the principal investigator offering brief presentations at community center postpartum depression support groups (e.g., YMCA) and exhibitions offered around Regina at sites frequented by women and families (e.g., Regina Family Expo, Welcome Wagon Baby Shower). Moreover, as evidence suggests that many women with PPD seek help from their obstetricians or primary care physicians (Yonkers & Chantilis, 1995), advertisements will be posted at medical clinics (e.g., general practitioner clinics, specialized obstetric clinics, mental health clinics). Healthcare providers across Saskatchewan will also be notified of this research and asked to provide referrals of any women under their care who might benefit from TAICBT for PPD as an alternative to in-person therapy. Finally, advertisements will also be posted at community organizations that offer mother-infant classes (e.g., “Mom and Baby Yoga” held at Birth Bliss Yoga Centre; Y’s Mom’s Group held at the YMCA) and will be placed around hospital maternity wards, pharmacies, community mental health clinics, medical clinics, and other appropriate public places around urban and rural southern Saskatchewan.

3. What will the participants be required to do in the course of the project?

All potential participants will participate in a telephone pre-screening and full-screening interview. When participants first contact the researcher, they will be informed of the nature of the study including the potential to be randomized to a waitlist condition (see Appendix X). The pre-screen interview will take approximately 15 minutes and will inquire about referral source, demographic questions, and questions about their symptoms (see Appendix B). To assess for severity of depressive symptoms, the Edinburgh Postnatal Depression Scale (Cox et al., 1987) will also be administered (see Appendix C). The full-screen interview will include additional demographic questions as well as the administration of the M.I.N.I. International Psychiatric Interview (M.I.N.I; Sheehan et al., 2006; see Appendices D and E). These interviews will be used to determine if they meet the inclusion and exclusion criteria established for the study.

As the current project is a randomized controlled trial, following the telephone screening and assessments, if the participant is eligible and interested in participating, she will be randomly assigned to either receive TAICBT treatment or to a 7-10 week waitlist group. Participants in both groups will be provided with a website link to a secure Canadian survey program (fluidsurveys.com). An information form and a consent form will be presented for the specific groups (see Appendices F and G). Each group will then be presented with a battery of baseline outcome measures (see Appendices H to M). These measures will take approximately 30 minutes to complete and will assess symptoms related to PPD, general stress, parental stress, quality of life, and treatment expectations.

***TAICBT treatment***: Following completion of the baseline measures, the participants in the TAICBT condition will be provided with a login username and password to access Maternal Depression Online, which will be located at the following address: <http://www.onlinetherapyuser.ca>. As part of clinical practice, with client consent, a letter will be sent to the participant’s family physician informing them that their patient is participating in the Maternal Depression Online program (see Appendix N). This is done for two reasons. The first is to verify the client’s identity, and the second reason is to have a contact number in the unlikely case of an emergency. Upon initial login to the website, participants are provided with an information page and consent form (see Appendix G). The participants will then be offered the seven-module online treatment, with each module taking approximately one week to complete. Each module provides education about the disorder and assigns suggested exercises and homework to be completed in consort with an online therapist. The therapist communicates with their client once a week through emails that are contained within the Online Therapy USER website. Upon login for a new module, participants complete a weekly mood monitor (see Appendix O) and provide examples and answers questions from the previous module (see Introduction pages from http://www.onlinetherapyuser.ca/sitemap).

In order to assess change over time, following treatment completion, participants will be emailed the same website link to fluidsurvey.com to complete the post-treatment outcome measures along with measures related to therapeutic alliance (see Appendices H to M). The measures will take approximately 30-45 minutes to complete and will assess change in symptoms over the course of treatment. In addition to the measures, the participants will be asked to respond to a series of follow-up, open-ended questions regarding their experiences with the program (see Appendix P) as well as questions regarding any additional treatment they sought over the 10-week period (see Appendix O).

***Pamphlet Condition***. In similar fashion to the TAU condition used in Austin and colleagues’ (2008) study, the participants who are randomly assigned to the TAU group will receive an information pamphlet that will be delivered via email (see Appendix Q). The MotherFirst campaign created an information pamphlet that is currently available to Saskatchewan healthcare providers, various support groups, and is displayed in places frequented by new mothers and their families. The chair of MotherFirst has agreed that the MotherFirst information pamphlet can be used for the purpose of the TAU condition. The pamphlet contains information on PPD symptoms and strategies to prevent and/or manage such symptoms. The pamphlet also includes a contact number and a website for postnatal support services offered in Saskatchewan (skmaternalmentalhealth.ca). Participants will be briefed over the telephone with the pamphlet information and informed of the postnatal support contacts should they choose to receive treatment. Seven to ten weeks following baseline measures, the participants will be emailed the same website link to fluidsurvey.com to complete the post-treatment outcome measures along with additional questions regarding any treatment they may have received during the wait time (see Appendix R). Following the completion of the post-treatment outcome measures, participants who received the pamphlet condition will be called over the telephone. They will be asked if they are still interested in receiving TAICBT. If they are screened eligible to participate according to the original screening criteria, the full treatment will be offered.

4. What information about the research project and their role will participants be given during the initial contact?

When participants first contact the unit they will be provided with a brief description of the study including the potential of being randomized to a 7-10 week wait list condition (see Appendix X). At this time a brief description of the Maternal Depression Online program will also be provided (i.e., number of modules, description of topics covered, the online therapist’s involvement in the client’s treatment). If a participant indicates interest in participation, she will be administered the prescreening interview (see Appendix B). If deemed eligible after the pre-screen interview, participants will be asked to participate in a more comprehensive telephone assessment (see Appendix D). They will be asked to verbally consent prior to starting this assessment (see Appendix D). If a participant is determined to be eligible to participate in the study after the telephone assessments, they will be notified if they have been randomized to receive TAICBT right away or if they will receive a pamphlet and informed that they will be offered the TAICBT after a 7-10 week wait period. At this time, they will be sent a website link that will include a consent form describing their role in participating, depending on which group they are assigned to (see Appendices F and G).

5. Will a consent form be used? If so, when will it be presented (e.g. immediately before interviews take place)?

Information and consent forms will be presented to the TAICBT participants (see Appendix G) and to the pamphlet control group participants (see Appendix F). Both consent forms will be presented online prior to completing baseline outcome measures. Prior to this, verbal consent will be obtained when participants undergo the comprehensive telephone assessment (see Appendix D). Participants assigned to the pamphlet control condition will also receive a consent form prior to starting the TAICBT treatment (see Appendix V).

6. a)Will participants be anonymous in the data gathering phase of the study?(Anonymous means that no link can be established between the participant and the research – no one including the researcher knows who has participated in the research)

No  Yes, the researcher only  Yes, no one including the researcher knows

1. Will the confidentiality of participants and their data be protected? (Confidentiality means that no link can be established between the collected information and the participant’s identity)

No

Yes

Yes, with the following limits:

Limits due to the nature of group activities (e.g. focus groups): the researcher cannot guarantee confidentiality

Limits due to context: individual participants could be identified because of the nature or size of the sample or because of their relationship with the researcher.

Limits due to selection: procedures for recruiting or selecting participants may compromise the confidentiality of participants (e.g., participants are referred to the study by a person outside the research team)

Other:

c) What assurances will participants be given and what precautions will be taken regarding the confidentiality of the data or information which they provide in the study?

Due to the nature of a treatment study, participants will have ongoing contact with a student therapist and supervisor and will therefore be identifiable to this individual and Dr. Heather Hadjistavropoulos. Participants will be informed that their confidentiality will be protected, as only the principal investigator, their therapist (if not Nicole Pugh), Dr. Hadjistavropoulos, and the Online Therapy Unit Co-ordinator (Marcie Nugent, MSW) will know their identity, and their identifying information will not be shared with anyone else. As part of clinical practice, with client consent, the client’s family physician will be informed that the client is participating in online therapy (see Appendix N). With client consent, the therapist may refer the client to another provider following treatment completion or termination from the program.

The student therapist and their supervisor will have access to participants’ outcome measures at the beginning and end-point of treatment in order to monitor symptoms, suicidal ideation (Item 10 on the EPDS, see Appendix C), and treatment progress, but will not have access to this information at follow-up, unless they are treated by Nicole Pugh.

Nicole Pugh will have access to de-identified client information, including progress in the Online Therapy USER program, demographic information, and responses to outcome measures. This information will be analyzed and presented in aggregate so that clients are not individually identified in any reports. As the research and clinical supervisor, only Dr. Hadjistavropoulos will have access to client information in both its identifiable and de-identified forms.

As part of clinical practice, clients will be informed that there are rare and limited circumstances under which confidentiality may need to be breached, and these circumstances include: (a) if a client poses an immediate threat to his/her life, or to another individual’s life;(b) if a client discloses information suggesting that any child is at risk of abuse;(c) if a client becomes involved in a legal case and the judge subpoenas information relevant to the legal problem;(d) if a client is concerned about his/her therapist’s professional conduct (or his/her supervisor’s), it may be necessary to release information from the file to evaluate and address this concern; and (e) if a client requests that information be released to another provider or insurer.

As an Internet-based research study, there is also a very small risk that participation in the research may compromise participants’ privacy. Participants will be informed of these possibilities on the information page prior to providing their consent for the overall study. Descriptions of these risks are as follows:

a) When submitting information to your therapist through the internet, including questionnaire answers, e-mail messages, exercises within the modules, and exercise check-ins at the beginning of modules, there is a possibility your information will be intercepted by unauthorized third parties using sophisticated tools. It should be noted that this rarely occurs, although it is a risk about which you should be advised. In order to limit this risk, the Online Therapy system utilizes encryption in the form of HTTPS to transmit the data both to and from yourself and your therapist. The data that are stored within the Online Therapy system, such as messages to your therapist, offline exercise examples, and responses to questionnaires, are encrypted with AES encryption. Furthermore, the system itself uses strict access controls whereby users of this system are only able to access their own information.

b) Any computer connected to the Internet will store information about visited websites on the Internet browser’s history list and its disk cache. The responses to the questionnaires are only temporarily stored on your computer until you close down your browser window. In other words, after you complete and submit your responses, your computer will automatically delete this information. You may also delete this information, as well as information about visiting the Online Therapy USER website by clearing your history list and disk cache. Your email messages are stored in the database, and on the server, that hosts the Online Therapy USER website. This sever is located in secure facilities at the University of Regina. The content of the messages are encrypted using AES encryption with 256bit key length. The Online Therapy system enforces strict access controls, and only your assigned therapist (and their supervisor if they are being supervised) can contact you, see your responses to exercises, and see your progress throughout therapy.

c) In regards to the questionnaires and questions about offline exercises that you will be filling out, after these are completed, the information you provide will be sent directly to the survey software website. The information will then be sent to a private folder that is only accessible by your therapist and researchers; however, your therapist will not have access to any questionnaires related to how you rate your therapist and the therapeutic process. All responses will be downloaded weekly and kept in a secure location by the researchers until completion of the study. The results will be stored on a secure file, and the information will not be linked to your Internet address.

Participants will be informed of these possibilities on the information page prior to providing their consent for the follow-up study. Descriptions of these risks are as follows:

a) The electronic submission of the participants responses may, in rare instances, be intercepted by unauthorized third parties using sophisticated tools. The likelihood of this occurring is rare and is a general risk when using a computer.

b) When using a computer that is connected to the Internet to access websites, information about the websites that participants visit will be stored in the Internet browser's history list and in its disk cache. This can be resolved by clearing the history list and disk cache. Note that responses to the questionnaires are only stored temporarily on the computer until the browser window is closed. Thus, after the responses are submitted, the computer automatically deletes this information.

c) Given the manner in which this study is being conducted, all of the questionnaire responses will be sent immediately to the survey software website. The survey software website then stores the responses in a database, and the data in the database are encrypted. The only people with access to this information are the client, the therapist, and the therapist supervisor if the therapist is being supervised.

7. Will children be used as a source of data?

Yes

No

If Yes, indicate how consent will be obtained on their behalf.

8. Describe any apparatus, substance, element of the physical environment or other materials that could cause harm to a participant if a side effect, malfunction, misuse accident or allergic reaction were to occur. If the participant comes into contact with a potentially hazardous apparatus or material, who is responsible for checking defects or malfunctions, and on what schedule will inspections be made? If participants come into contact with some substance that could cause harm, please document your safeguards.

This is not applicable for the present study.

1. Will deception of any kind be necessary in the project?

Yes

No

If yes, explain why.

10. Describe any debriefing procedures that will be used. (Note that if deception is used, debriefing is necessary).

Debriefing will not be used for this study.

11. Will participants be compensated?

Yes

No

If yes, explain how and when they will be compensated and why you think that amount and form of compensation is appropriate.

# Section IV: Access to Data and Findings

1. Who will have access to the original data? (For example: co-investigators, students). How will all those who have access to the data be made aware of their responsibilities concerning privacy and confidentiality?

The principal investigator, Dr. Hadjistavropoulos, and Unit Coordinator (Marcie Nugent) will have access to the de-identified data for data entry and then for storage purposes. An undergraduate student who is seeking volunteer experience in our research lab may also be involved in data entry. He or she will be asked to sign a *Declaration of Confidentiality and Non-Disclosure* form that is required of all volunteers in the Online Therapy Unit (see Appendix S). When qualitative data are coded by the principal investigator and a second non-biased researcher, all identifying information will be removed from the participants’ responses to ensure privacy and confidentiality.

1. How do you anticipate disseminating your research results?
2. Directly to participants, describe how (e.g. website location of findings, location of published study, etc)

The results of this study will be published on the Online Therapy Unit’s website ([www.onlinetherapyuser.ca](http://www.onlinetherapyuser.ca)) and on our research lab’s website (<http://uregina.ca/~hadjista/results.html>). This information is outlined in the consent form.

b) Check all others that apply:

Thesis/Dissertation/class presentation Media (e.g. newspaper, radio, TV) Presentations at scholarly meetings Published article, chapter or book

Internet Other, explain:

3. Describe your plans for protecting data as well as preserving or destroying data after the research is completed. For all data (e.g. paper records, audio or visual recordings, electronic recordings), indicate the:

a) means and location of storage (e.g. a locked filing cabinet, password protected computer files)

b) time duration of storage. (REB requires that data be archived for a minimum of three (3) years)

c) final disposition (archive, shredding, electronic file deletion)

(See Section IV-3 of the Guidelines)

In terms of clinical practice, the professional standard is to retain client records for a period of seven years. Hard-copy client records (i.e., the screening and assessment materials) will be locked in a secure cabinet and a secure office after data analysis and publication. E-mail correspondence between therapist and client will be retained on the secure website. The only individuals who can access this information will be the principal investigator (Nicole Pugh), her supervisor (Dr. Heather Hadjistavropoulos), the client’s therapist if not Nicole Pugh, and the Online Therapy USER coordinator (Marcie Nugent). After seven years, the paper records will be destroyed through shredding, and the e-mail correspondence will be downloaded and saved to a hard drive and then securely deleted.

**LIST OF APPENDICES**

APPENDIX A. ADVERTISMENT EXAMPLE FOR MATERNAL DEPRESSION ONLINE

APPE**N**DIX B. PRE-SCREEN TELEPHONE INTERVIEW

APPENDIX C. EDINBURGH POSTNATAL DEPRESSION SCALE (EPDS)

APPENDIX D. FULL SCREEN INTERVIEW

APPENDIX E. THE MINI-INTERNATIONAL NEUROPSYCHIATRIC INTERVIEW (MINI)

APPENDIX F. INFORMATION PAGE AND CONSENT FORM FOR WAITLIST CONTROLPARTICIPANTS

APPEDIX G. INFORMATION PAGE AND CONSENT FORM FOR THERAPIST ASSISTED ICBT (TAICBT) PARTICIPANTS

APPENDIX H. DEPRESSION ANXIETY STRESS SCALE (DASS-21)

APPENDIX I. PARENTAL STRESS INDEX-SHORT FORM (PSI-SF)

APPENDIX J. WORLD HEALTH ORGANIZATION QUALITY OF LIFE ASSESSMENT BREF (WHOQOL-BREF)

APPENDIX K. THE THERAPEUTIC ALLIANCE QUESTIONNAIRE (TAQ)

APPENDIX L. THE TREATMENT SATISFACTION QUESTIONNAIRE (TSQ)

APPENDIX M. CREDIBILITY/EXPECTANCY QUESTIONNAIRE (CEQ)

APPENDIX N. PHYSICIAN NOTIFICATION FORM

APPENDIX O. WEEKLY MOOD MONITOR

APPENDIX P. FOLLOW-UP QUESTIONS

APPENDIX Q. MOTHERFIRST AND CONTROL CONDITION PAMPHLET

APPENDIX R. 10-WEEK TREATMENT QUESITONS

APPENDIX S. VOLUNTEER CONFIDENTIALITY FORM

APPEDIX T. INFORMATION PAGE FOR THERAPIST ASSISTED ICBT (TAICBT) PARTICIPANTS, TIME 2

APPENDIX U: LIST OF ALTERNATIVE RESOURCES

APPENDIX V. INFORMATION PAGE AND CONSENT FORM FOR WAITLISTED PARTICIPANTS

APPDENDIX W. PROTOCOL TO ADDRESS A CLIENT WHO IS AT RISK OF SUICIDE

APPENDIX X. TELEPHONE SCRIPT (USED BEFORE PRE-SCREENING INTERVIEW)

APPDENDIX Y. PROTOCOL TO ADDRESS A CLIENT WHO INDICATES PARTNER ABUSE

APPENDIX Z: LIST OF SHELTERS IN SASKACHEWAN

APPENDIX A. ADVERTISMENT FOR MATERNAL DEPRESSION ONLINE


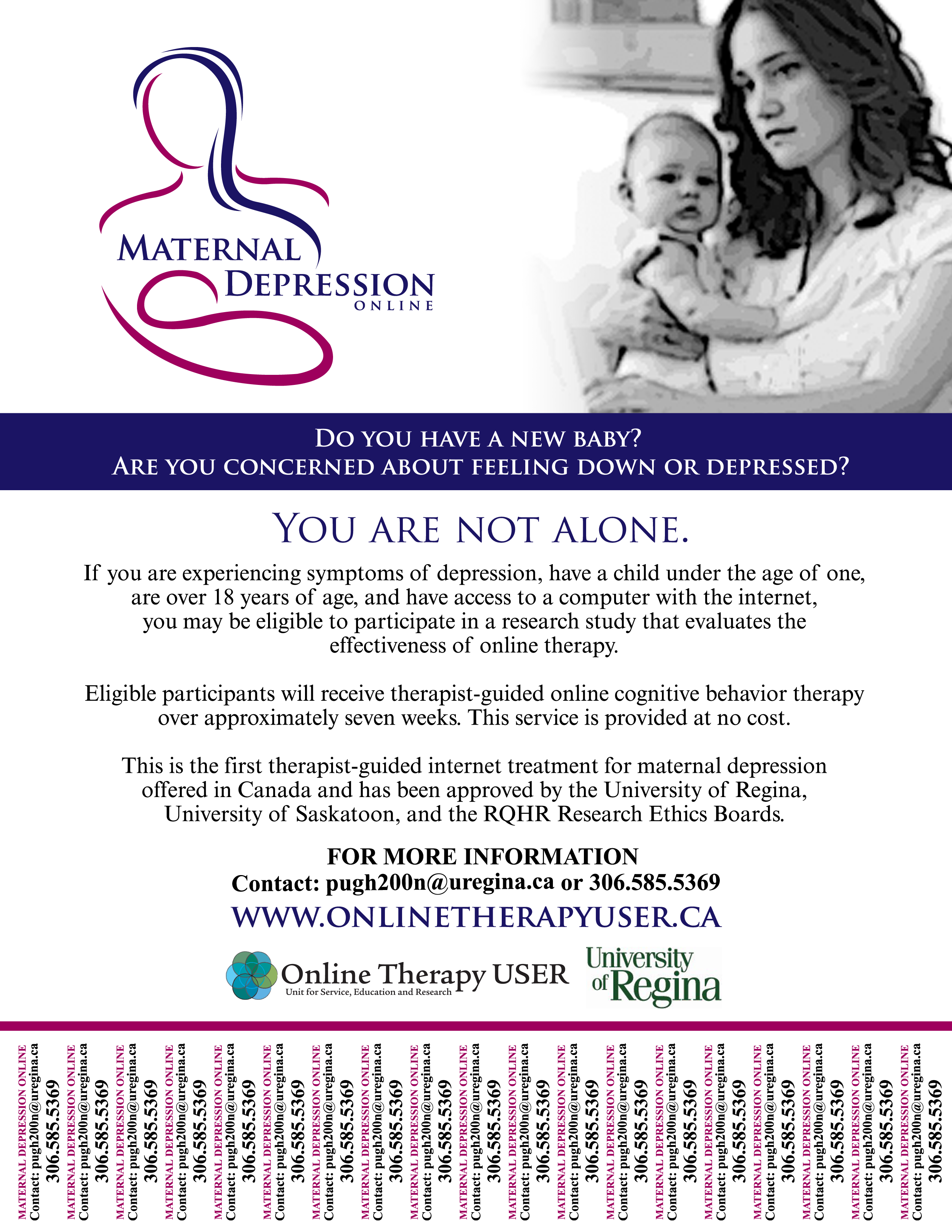


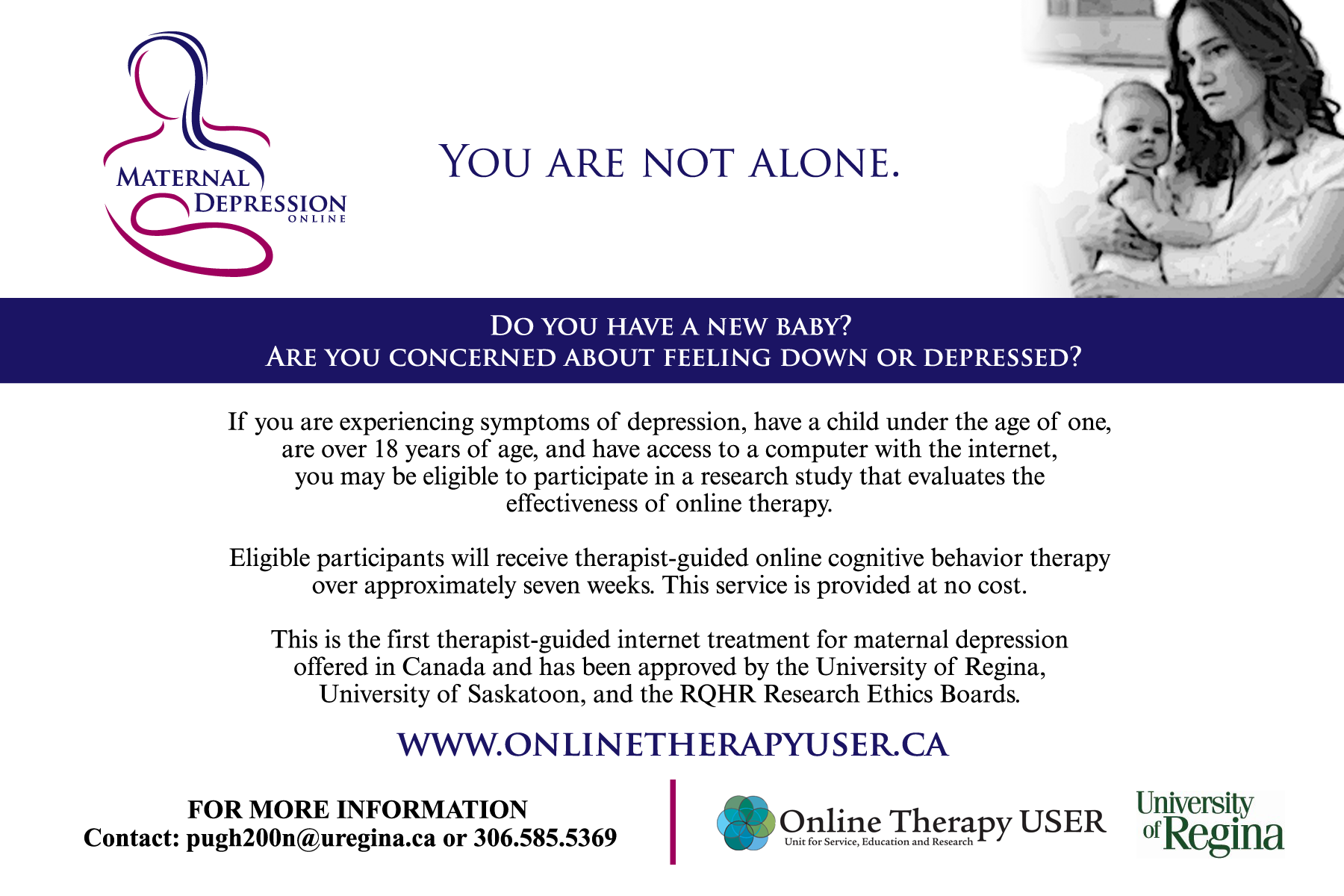
APPENDIX B. PRE-SCREEN TELEPHONE INTERVIEW


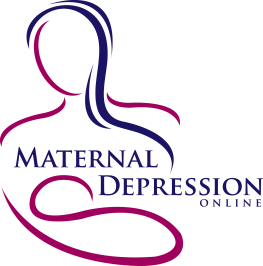
**Pre-Screening Interview**

**Participant ID# Date:**

****Read Pre-Screening Script*(See Appendix X)***

***So now that you know about the formatting of the program and the research involved, does this still sound like something that you would be interested in?***

Yes (continue)

No… why? ___________________________________Screened out.

* IF SCREENED OUT REFER TO APPROPRIATE RESOURCES.

***Thanks for your interest in Maternal Depression Online. Can I ask how you heard about us?***

Referral from physician, social worker, nurse, other healthcare provider (not currently an eTherapist)

Referral from Intake (Health Region)

Referral from health care provider who is currently an eTherapist

**Name of e-therapist**______________________ **Agency:**______________________

Referral from another patient

Online advertisement: Which one? ___________________________

Newsletter: Which one? ___________________________________

Newspaper: Which one? __________________________________

Poster/brochure advertisement in community: Whereabouts? __________________________

Found Online Therapy USER website one own

Other; Please describe below:

**Limits of Confidentiality.**Before we begin our pre-screening interview, I have to inform you that although the interview will be confidential, there are certain limits to confidentiality including:

- If you pose an immediate threat to your life, or another individual’s life; or
- If you disclose information suggesting that any child is at risk of abuse, confidentiality will be broken.

   **Client understands confidentiality.**

| ***Demographic Data*** | | | | |
| --- | --- | --- | --- | --- |
| Client Name: | Date of Birth (DD/MM/YYYY) | | | |
| Address: | Appt #: | | | |
| City: | Postal Code: | | | |
| Phone No.:(home)  (work)  (other)  Phone No. during the day: | Can we contact you by: (Y or N)  Phone _______ Letter______  Leave Message____ **Email**____ | | | |
| Health Card No.: |  | | | |
| ***Exclusion Criteria*** | | | |  |
|  | | **Appropriate** | **Not Appropriate** |  |
| 1. Are you a Saskatchewan resident? | | Yes | No |  |
| 2. How old are you? _________ years | | Over 18 | Under 18 |  |
| 3. Do you have a **child** under the age of **one**? | | Yes | No |  |
| 4. Do you have access to a computer and printer at home? | | Yes | No |  |
| 5. Do you have access to the Internet at home? | | Yes | No |  |
| 6. Do you feel comfortable using the Internet? | | Yes | No |  |
| 7. Do you feel comfortable writing e-mails? | | Yes | No |  |
| 8. Would you be willing to have your physician, a medical clinic, or an emergency hospital be notified of your participation in this program?  Doctor’s Name:_________________________________  Medical Clinic:__________________________________  Telephone Number:_______________________________ | | Yes | No |  |

9. How far did you go in school?

Less than Grade 6  College/technical certificate; some university

Less than Grade 9  University undergraduate degree(s)

Less than Grade 12  University graduate degree(s)

High school diploma or GED

10. How comfortable would you say you are with writing out your thoughts and describing your feelings in words? Would you say you are…

| *Not very comfortable* | *A little comfortable* | *Somewhat comfortable* | *Fairly comfortable* | *Very comfortable* |
| --- | --- | --- | --- | --- |
| 1 | 2 | 3 | 4 | 5 |

11. Do you have any concerns about your ability to read or write?

______________________________________________________________________________

****Administer the EPDS. (Now I am going to ask you a few questions about your mood)****

APPENDIX C. EDINBURGH POSTNATAL DEPRESSION SCALE (EPDS)^[[1]](#footnote-1)^

| **As you have recently had a baby, we would like to know how you are feeling.  Please UNDERLINE the answer which comes closest to how you have felt  IN THE PAST 7 days, not just how you feel today.** |
| --- |

**In the Past 7 days:**

1. I have been able to laugh and see the funny side of things
 As much as I always could
 Not quite so much now
 Definitely not so much now
 Not at all

2. I have looked forward with enjoyment
 As much as I ever did
 Rather less than I used to
 Definitely less that I used to
 Hardly at all

3. I have blamed myself unnecessarily when things went wrong
 Yes, most of the time
 Yes, some of the time
 Not very often
 No, never

4. I have been anxious or worried for no good reason
 No, not at all
 Hardly ever
 Yes, sometimes
 Yes, very often

5. I have felt scared or panicky for no very good reason
 Yes, quite a lot
 Yes, sometimes
 No, not much
 No, not at all

6. Things have been getting on top of me
 Yes, most of the time I haven’t been able to cope at all
 Yes, sometimes I haven’t been coping as well as usual
 No, most of the time I have coped quite well
 No, I have been coping as well as ever

7. I have been so unhappy that I have had difficulty sleeping
 Yes, most of the time
 Yes, sometimes
 Not very often
 No, not at all

8. I have felt sad or miserable
 Yes, most of the time
 Yes, quite often
 Not very often
 No, not at all

9. I have been so unhappy that I have been crying
 Yes, most of the time
 Yes, quite often
 Only occasionally
 No, never

10. The thought of harming myself has occurred to me
 Yes, quite often
 Sometimes
 Hardly ever
 Never

Appendix D. FUll Screen InteRview
 *BEFORE WE BEGIN OUR INTERVIEW, I HAVE TO INFORM YOU THAT ALTHOUGH THE INTERVIEW WILL BE CONFIDENTIAL, THERE ARE CERTAIN LIMITS TO CONFIDENTIALITY INCLUDING: IF YOU POSE AN IMMEDIATE THREAT TO YOUR LIFE, OR ANOTHER INDIVIDUAL’S LIFE, AND IF YOU DISCLOSE INFORMATION SUGGESTING THAT ANY CHILD IS AT RISK OF ABUSE, CONFIDENTIALITY WILL BE BROKEN.**Client UNDERSTANDS LIMITS of confidentiality*

| ***Demographic Data*** | |
| --- | --- |
| Client Name: | Date of Birth (DD/MM/YYYY) |
| Address: | Appt #: |
| City: | Postal Code: |
| Phone No.: (home)  (work)  (other)  Phone No. during the day: | Can we contact you by: (Y or N)  Phone _______ Letter______  Leave Message____ Email_____ |
| Health Card No.: |  |
| Date of Screening: |  |

*Are you currently on any medication for any of the problems that I asked you about today?*

Yes (List medications) ______________________________________________

___________________________________________________________________

___________________________________________________________________

No

If ‘YES’: *For how long have you been taking the medication?* _____________________

______________________________________________________________________
*When did you last have a change in the dosage?_______________________________*

______________________________________________________________________
(If less than 1 month: inform the person that we will need to wait until the medication has been stabilized for 1 month)

*Will you inform us if your medication changes or if you begin a new medication?*

Yes  No

*Are you currently in (psychological) treatment for any of the problems that I asked about today?*  Yes  No

*Will you tell us if you begin treatment for any of the problems that I asked you about today?*  Yes  No

*Have you previously been in treatment for any of the problems that I asked about today?*  Yes  No

If ‘Yes’, *when?* _____________________________________________________________________

*What kind of therapy was it?* ______________________________________________________________________

Do you have involvement with other agencies: (Addiction Services, Crisis Services, RCMP, Social Services)

_____________________________________________________________________

*Do you have any illnesses or medical conditions?* (List below) ____________________

_______________________________________________________________________

*Is there a possibility that this/these could interfere with your ability to participate in this* ***Online Therapy USER*** *program?*  Yes  No

*In order to provide some background information to your therapist, can I ask you a few personal questions?*

**Personal History:**

Ethnicity:

Marital Status:

Spousal or partner problems:

Will your partner know that you are participating?  Yes  No

How many children do you have? What are their ages?

Do you use childcare services (e.g., daycare, preschool)?  Yes  No

If yes, please explain:­­­­­­____________________________________________________________________

Please answer the following questions regarding your *most recent child*:

Vaginal Delivery: Yes  No

Breastfeed: Yes  No

Twin Births: Yes  No

Complications with delivery: Yes  No

If yes, please explain_________________________________________________________

Any health concerns with the infant? Yes  No

If yes, please explain_________________________________________________________

**Additional Demographic Questions:**

Family Psychiatric/Medical History (e.g. Depression, Addictions etc.)___________________________________________________________________________

Abuse Within/Outside the Family (e.g. Sexual, Physical, Verbal, Emotional)______________________________________________________________________________
Who do you turn to for support? ___________________________________________________

Are you currently working outside of the home or are you on maternity leave?

Employer? ____________________________________

How long have you done this? _________________________________months / years

Total length of maternity leave?

Living Arrangement:  Alone  With Family  With Friends
 Other: _____________________________________________________________________________

Are you facing any current legal difficulties right now?:

**Medical History:**

Prescription Drugs:

Non-Prescription Drugs:

Addictions:

Tobacco Use:

Alcohol Use:

Illicit Drugs:

Gambling:

Treatment:

*Thanks for answering these questions.*

*Do you have any questions for me?*

APPENDIX E. THE MINI-INTERNATIONAL NEUROPSYCHIATRIC INTERVIEW (MINI)^[[2]](#footnote-2)^

**A. MAJOR DEPRESSIVE EPISODE (MINI PLUS)**

**(**→** means: go to the diagnostic boxes, circle NO in all diagnostic boxes, and move to the next module)**

A1 a Have you **ever** been consistently depressed or down, most of the day, NO YES

nearly every day, for at least two weeks?

If **A1a** = **yes**:

b Have you been consistently depressed or down, most of the day, nearly every day, NO YES

for the past 2 weeks?

A2 a Have you **ever** been much less interested in most things or much less able to enjoy NO YES

the things you used to enjoy most of the time over at least 2 weeks?

If **A2a** = **yes**:

b In the past 2 weeks, have you been much less interested in most things or much NO YES

less able to enjoy the things you used to enjoy most of the time.

→** **

is **A1a** or **A2a** coded **YES?** NO YES

**if currently depressed** (**A1b** or **A2b** = **YES**), **explore the current episode AND the most symptomatic past episode.** otherwise explore the most symptomatic past episode.

A3 **Over the two week period when you felt depressed or uninterested,**

Current Episode Past Episode

a Was your appetite decreased or increased nearly every day? Did NO YES NO YES

your weight decrease or increase without trying intentionally

**(**i.e., by ±5% of body weight or ±8 lbs. or ±3.5 kgs. for a

160 lb./70 kgs.person in a month)? If **YES** to either, code **YES**.

b Did you have trouble sleeping nearly every night NO YES NO YES

(difficulty falling asleep, waking up in the middle of the night,

early morning wakening or sleeping excessively)?

c Did you talk or move more slowly than normal or were you fidgety, NO YES NO YES

restless or having trouble sitting still almost every day?

d Did you feel tired or without energy almost every day? NO YES NO YES

e Did you feel worthless or guilty almost every day? NO YES NO YES

IF **A3e** = **YES**: ask for an example*.*The example is consistent with a delusional idea. No Yes

f Did you have difficulty concentrating or making decisions NO YES NO YES
almost every day?

g Did you repeatedly consider hurting yourself, feel suicidal, NO YES NO YES

or wish that you were dead?

→** **

A4 Are **3** or more  **A3** answers coded **YES** (or **4 A3** answers, NO YES NO YES

if **A1a** or **A2a** are coded **NO** for past episode or if **A1b** or **A2b** are coded **NO** for current episode)?

**Verify if the positive symptoms occurred during the same 2 week time frame.**

If **A4** is coded **NO** for current episode then explore **A3a** - **A3g** for most symptomatic past episode.

→** **

A5 Did the symptoms of depression cause you significant distress or impair NO YES

your ability to function at work, socially, or in some other important way?

A6 Are the symptoms due entirely to the loss of a loved one (bereavement)

and are they similar in severity, level of impairment, and duration to

what most others would suffer under similar circumstances?

If so, this is uncomplicated bereavement.

→** **

has uncomplicated bereavement been ruled out? NO YES

A7 a Were you taking any drugs or medicines just before these symptoms began?

No  Yes

b Did you have any medical illness just before these symptoms began?

No  Yes

in the clinician’s judgment: are either of these likely to be direct causes of the patient's depression?
If necessary ask additional open-ended questions.

**A7 (summary**):has an organic cause been ruled out? NO YES UNCERTAIN

| A8code **yes** if **A7(summary)** = **yes** or **uncertain**.  Specify if the episode is current AND/ or past OR BOTH (recurrent). | **NO YES**  ***Major Depressive Episode***  Current  Past |
| --- | --- |

| A9code **yes** if **A7b** = **yes** and**A7 (summary)** = **no**.  Specify if the episode is current AND/ or past OR BOTH (recurrent). | **NO YES**  ***Mood Disorder Due to a***  ***General Medical Condition***  Current  Past |
| --- | --- |

| A10code **yes** if **A7a** = **yes** and **A7 (summary)** = **no**.  Specify if the episode is current AND/ or past OR BOTH (recurrent). | **NO YES**  ***Substance Induced Mood Disorder***  Current  Past |
| --- | --- |

CHRONOLOGY

| A11 | How old were you when you first began having symptoms of depression? |  | age |  |  |  |  |
| --- | --- | --- | --- | --- | --- | --- | --- |

| A12 | During your lifetime, how many distinct times did you have these symptoms |  |  |  |  |  |  |
| --- | --- | --- | --- | --- | --- | --- | --- |

of depression (daily for at least 2 weeks)?

| A13 | Is there any family history of bipolar disorder or any relative ever treated with a  mood stabilizer? | Yes |  | No |  |  |  |
| --- | --- | --- | --- | --- | --- | --- | --- |

**SUBTYPES OF MAJOR DEPRESSIVE EPISODE Mark all that apply.**

Mild  296.21/296.31

Moderate  296.22/296.32

Severe without psychotic features  296.23

Severe with psychotic features  296.24

In partial remission  296.25

In full remission  296.26

Chronic

With catatonic features

With melancholic features

With atypical features

With postpartum onset

With seasonal pattern

With full interepisode recovery

Without full interepisode recovery

**B. SUICIDALITY (MINI)**

Points

**In the past month did you:**

B1 Suffer any accident? This includes taking too much of your medication accidentally. NO YES 0

IF NO TO B1, SKIP TO B2; IF YES, ASK B1a:

**B**1a Plan or intend to hurt yourself in any accident either actively or passively (e.g. by not avoiding a risk)? NO YES 0

IF NO TO B1a, SKIP TO B2: IF YES, ASK B1b:

B1b Intend to die as a result of any accident? NO YES 0

B2 Feel hopeless? NO YES 1

B3 Think that you would be better off dead or wish you were dead? NO YES 1

B4 Think about hurting or injuring yourself or have mental images of harming yourself, NO YES 4 with at least some intent or awareness that you might die as a result?

How many times? _____

B5 Think about suicide (killing yourself)? NO YES 6

How many times? _____

IF NO TO B5, SKIP TO B7. OTHERWISE ASK:

Frequency Intensity

Occasionally π Mild π

Often π Moderate π

Very often π Severe π

B6 Feel unable to control these impulses? NO YES 8

| B7 | Have a suicide method or plan in mind (e.g. how, when or where)? | NO | YES | 8 |
| --- | --- | --- | --- | --- |

IF NO TO B7, SKIP TO B9.

NOTE: IF PARTICIPANT HAS A SUICIDE PLAN OR RESPONDS ‘YES’ TO ANY OF THE ITEMS FROM B7 ONWARD, END INTERVIEW AND REFER TO ALTERNATIVE RESOURCES.

B8 Intend to follow through on a suicide plan? NO YES 8

B9 Intend to die as a result of a suicidal act? NO YES 8

B10 Take any active steps to prepare to injure yourself or to prepare for a suicide attempt

in which you expected or intended to die? NO YES 9

How many times? _____

B11 Injure yourself on purpose without intending to kill yourself? NO YES 4

B12 Attempt suicide (to kill yourself)? NO YES 9

A suicide attempt means you did something where you could possibly be injured,

with at least a slight intent to die.

IF NO, SKIP TO B13:

How many times? _____

Hope to be rescued / survive π

Expected / intended to die π

**In your lifetime:**

B13 Did you ever make a suicide attempt (try to kill yourself)? NO YES 4

“A suicide attempt is any self-injurious behavior, with at least some intent (> 0) to die as a result or if intent can be inferred, e.g. if it is clearly not an accident or the individual thinks the act could be lethal, even though denying intent.”

(C-CASA definition). Posner K et al. Am J Psychiatry 164:7, July 2007.

| is at least **1** of the above (EXCEPT B1) coded **yes**?  If yes, add the total points for the answers (B1-B13)  checked ‘yes’ and specify the suicidality score as indicated in the diagnostic box:  make any additional comments about your assessment of this patient’s current  and near future suicidality in the space below:  If you do start having thoughts of suicide, or you begin to have a plan around suicide during this program, please make sure to let your therapist know. Since your therapist may not check their emails every day, it would be best if you phone them. You can also phone the Online Therapy Unit and speak to the co-ordinator for this project, Marcy Nugent. If you are unable to reach your therapist or the co-ordinator, there is a suicide prevention tab on the website that lists other agencies you can call as well. If you feel as though you are at risk for committing suicide, you can also call 911 for help. | ***NO YES***  ***SUICIDALITY***  ***CURRENT***  1-8 points Low π  9-16 points Moderate π  > 17 points High π   |
| --- | --- |

**Participant must have LOW Current Suicide Risk to participate in program. If > Low, DISCUSS OTHER OPTIONS FOR TREATMENT; DOCUMENT DISCUSSION; CONSULT WITH SUPERVISOR IMMEDIATELY.

**C. MANIC AND HYPOMANIC EPISODES (MINI)**

**(**** means:go to the diagnostic boxes, circle NO in manic and hypomanic diagnostic boxes, and move to next module)**

Do you have any family history of manic depressive illness or bipolar disorder,

or any family member who had mood swings treated with a medication like lithium, NO YES

sodium valproate (Depakote) or lamotrigine (Lamictal)?

THIS QUESTION IS NOT A CRITERION FOR BIPOLAR DISORDER, BUT IS ASKED TO INCREASE

THE CLINICIAN’S VIGILANCE ABOUT THE RISK FOR BIPOLAR DISORDER .

IF YES, PLEASE SPECIFY WHO:________________________________________

C1 a Have you **ever** had a period of time when you were feeling 'up' or 'high' or ‘hyper’ NO YES

or so full of energy or full of yourself that you got into trouble, - or that

other people thought you were not your usual self? (Do not consider

times when you were intoxicated on drugs or alcohol.)

if patient is puzzled or unclear about what you mean

by 'up' or 'high' or ‘hyper’, clarify as follows:By 'up' or 'high' or ‘hyper’

I mean: having elated mood; increased energy; needing less sleep; having rapid

thoughts; being full of ideas; having an increase in productivity, motivation,

creativity, or impulsive behavior; phoning or working excessively or spending more money.

IF NO, CODE NO TO **C1b**: if **yes** ask:

b Are you currently feeling ‘up’ or ‘high’ or ‘hyper’ or full of energy? NO YES

C2 a Have you **ever** been persistently irritable, for several days, so that you NO YES

had arguments or verbal or physical fights, or shouted at people outside

your family? Have you or others noticed that you have been more irritable

or over reacted, compared to other people, even in situations that you felt

were justified?

IF NO, CODE NO TO **C2b**: if **yes** ask:

b Are you currently feeling persistently irritable? NO YES



is **C1a** or **C2a** coded **yes**? NO YES

C3 if **C1b** or **C2b** = **yes**: explore THE **current** AND THE MOST SYMPTOMATIC **PAST** episode, otherwise

if **C1b** and **C2b** = **no**: explore ONLY the most symptomatic **past** episode

**During the times when you felt high, full of energy, or irritable did you:**

Current Episode Past Episode

a Feel that you could do things others couldn't do, or that you were an NO YES NO YES

especially important person? If **yes**, ask for examples**.**

The examples are consistent with a delusional idea. Current Episode πNo πYes

Past Episode πNo πYes

b Need less sleep (for example, feel rested after only a few hours sleep)? NO YES NO YES

c Talk too much without stopping, or so fast that people had difficulty NO YES NO YES

understanding?

d Have racing thoughts? NO YES NO YES

Current Episode Past Episode

e Become easily distracted so that any little interruption could distract you? NO YES NO YES

f Have a significant increase in your activity or drive, at work, at school, NO YES NO YES

socially or sexually or did you become physically or mentally restless?

g Want so much to engage in pleasurable activities that you ignored the risks or NO YES NO YES

consequences (for example, spending sprees, reckless driving, or sexual

indiscretions)?

C3Summary**:** when rating current episode: NO YES NO YES

if C1b is NO**,** are 4 or more C3 answers coded YES?

if C1b is YES, are 3 or more C3 answers coded YES?

when rating past episode:

if C1a is NO, are 4 or more C3 answers coded YES?

if C1a is YES, are 3 or more C3 answers coded YES?

code YES only if the above 3 or 4 symptoms occurred during the same time period.

rule: elation/expansiveness requires only three C3 symptoms, while

irritable mood alone requires 4 of the C3 symptoms.

C4 What is the longest time these symptoms lasted?

1. 3 days or less π π
2. 4 to 6 days π π
3. 7 days or more  π π

C5 Were you hospitalized for these problems? NO YES NO YES

IF YES, STOP HERE AND CIRCLE YES IN MANIC EPISODE FOR THAT TIME FRAME.

C6 Did these symptoms cause significant problems at home, at work, socially NO YES NO YES

in your relationships with others, at school or in some other important way?

| Are **C3** summary and **C5** and **C6** coded **yes**?  or  are **C3** summary and **C4c** and **C6** coded **yes** and is **C5** coded **no**?  specify if the episode is current and / or past. | **NO YES**  ***MANIC EPISODE***  CURRENT π  PAST π |
| --- | --- |

| **Is C3** summary **coded YES** and are **C5** and **C6** coded **no** and is either **C4b** or **C4c** coded **yes**?  or  are **C3** summary and **C4b** and **C6** coded **yes** and is **C5** coded **no?**  specify if the episode is current and / or past.  if **yes** to current manic episode, then code current hypomanic episode as **no.**  if **yes** to past manic episode, then code past hypomanic episode as **not explored.** | ***HYPOMANIC EPISODE***  CURRENT π **NO**   π **YES**  PAST π **NO**   π **YES**  π **NOT EXPLORED** |
| --- | --- |

| are **C3** summaryand **C4a** coded **yes** and is **C5** coded **no**?  specify if the episode is current and / or past.  if **yes** to current manic episode or hypomanic episode,  then code current hypomanic symptoms as **no.**  if **yes** to past manic episode or yes to past hypomanic episode,  then code past hypomanic symptoms as **not explored.** | ***HYPOMANIC SYMPTOMS***  CURRENT π **NO**   π **YES**  PAST π **NO**   π **YES**   π **NOT EXPLORED** |
| --- | --- |

C7 a) IF MANIC EPISODE IS POSITIVE FOR EITHER CURRENT OR PAST ASK:

Did you have 2 or more of these (manic) episodes lasting 7 days or more (**C4c**) in your

lifetime (including the current episode if present)?     NO      YES

b) IF HYPOMANIC EPISODE IS POSITIVE FOR EITHER CURRENT OR PAST ASK:

Did you have 2 or more of these (hypomanic) episodes lasting just 4 to 6 days (**C4b**)

in your lifetime (including the current episode)?             NO      YES

c) IF THE PAST “HYPOMANIC SYMPTOMS” CATEGORY IS CODED POSITIVE ASK:

Did you have these hypomanic symptoms lasting only 1 to 3 days (**C4a**) 2 or more times

in your lifetime, (including the current episode if present)?   NO     YES

**D. GENERALIZED ANXIETY DISORDER (MINI PLUS)**

**(**→** means: go to the diagnostic boxes, circle NO in all diagnostic boxes, and move to the next module)**

→** **

P1 a Have you worried excessively or been anxious about several things NO YES

over the past 6 months?

→** **

b Are these worries present most days? NO YES

→** **

is the patient’s anxiety restricted exclusively NO YES

to, or better explained by, any disorder prior to this point?

→** **

P2 Do you find it difficult to control the worries or do they interfere with your ability to NO YES

focus on what you are doing?

P3 for the following, code **no**, if the symptoms are confined to features of any disorder explored prior to this point. (E.G. PRIMARILY IN SOCIAL SITUATIONS, TRIGGERED PRIMARILY BY MEMORIES OF A TRAUMATIC EVENT OR PRIMARILY TO FEARS OF HAVING A PANIC ATTACK)

**When you were anxious over the past 6 months, most of the time did you:**

a Feel restless, keyed up or on edge? NO YES

b Feel tense? NO YES

c Feel tired, weak or exhausted easily? NO YES

d Have difficulty concentrating or find your mind going blank? NO YES

e Feel irritable? NO YES

f Have difficulty sleeping (difficulty falling asleep, waking up in the middle of the night, NO YES

early morning wakening, or sleeping excessively)?

→** **

**summary of P3:** are **3** or more **P3** answers coded **yes**? NO YES

→** **

P4 Did these symptoms of anxiety cause you significant distress or impair your ability NO YES

to function at work, socially, or in some other important way?

P5 a Were you taking any drugs or medicines just before these symptoms began?

No  Yes

b Did you have any medical illness just before these symptoms began?

No  Yes

in the clinician’s judgment: areeither of these likely to be direct causes of the patient's generalized anxiety disorder?

**P5 (summary):**has an organic cause been ruled out? NO YES

| is **P5 (summary)** coded **yes**? | **NO YES**  ***Generalized Anxiety Disorder***  **CURRENT** |
| --- | --- |

| P6 is **P5 (summary)** coded **no** and **P5b**coded **yes**? | **NO YES**  **Current**  ***Generalized Anxiety Disorder.***  **Due to a General**  **Medical Condition** |
| --- | --- |

| P7 is **P5 (summary)** coded **no** and **P5a**coded **yes**? | **NO YES**  **Current**  **Substance Induced**  ***Generalized Anxiety Disorder*** |
| --- | --- |

CHRONOLOGY

| P8 | How old were you when you first began having symptoms of generalized anxiety? |  | age |  |  |  |  |
| --- | --- | --- | --- | --- | --- | --- | --- |

| P9 | During the past year, for how many months did you have significant symptoms of |  |  |  |  |  |  |
| --- | --- | --- | --- | --- | --- | --- | --- |

generalized anxiety?

**E. ALCOHOL DEPENDENCE / ABUSE (MINI)**

**(**** means: go to diagnostic boxes, circle NO in both and move to the next module)**

** **

I1 **In the past 12 months**, have you had 3 or more alcoholic drinks, - within a NO YES

3 hour period, - on 3 or more occasions?

I2 **In the past 12 months**:

a Did you need to drink a lot more in order to get the same effect that you got when you first NO YES

started drinking or did you get much less effect with continued use of the same amount?

b When you cut down on drinking did your hands shake, did you sweat or feel agitated? Did NO YES

you drink to avoid these symptoms (for example, "the shakes", sweating or agitation)

or to avoid being hungover?

if **yes** to any, code **yes**.

c During the times when you drank alcohol, did you end up drinking more than NO YES

you planned when you started?

d Have you tried to reduce or stop drinking alcohol but failed? NO YES

e On the days that you drank, did you spend substantial time obtaining NO YES

alcohol, drinking, or recovering from the effects of alcohol?

f Did you spend less time working, enjoying hobbies, or being with others NO YES

because of your drinking?

g If your drinking caused you health or mental problems, NO YES

did you still keep on drinking?

| are **3** or more **I2** answers coded **yes**?  ***** IF YES, SKIP I3 QUESTIONS and go to next module. “DEPENDENCE PREEMPTSABUSE” in dsm iv TR. | **NOYES***  ***ALCOHOL DEPENDENCE***  **CURRENT** |
| --- | --- |

I3 **In the past 12 months:**

a Have you been intoxicated, high, or hungover more than once when you had other NO YES

responsibilities at school, at work, or at home? Did this cause any problems?

(code **yes** only if this caused problems.)

b Were you intoxicated more than once in any situation where you were physically at risk, NO YES

for example, driving a car, riding a motorbike, using machinery, boating, etc.?

c Did you have legal problems more than once because of your drinking, for example, NO YES

an arrest or disorderly conduct?

d If your drinking caused problems with your family or other people, NO YES

did you still keep on drinking?

| are **1** or more **I3** answers coded **yes**? | **NO YES**  ***ALCOHOL ABUSE***  **CURRENT** |
| --- | --- |

**F. SUBSTANCE DEPENDENCE / ABUSE (NON-ALCOHOL) (MINI)**

**(**** means: go to the diagnostic boxes, circle NO in all diagnostic boxes, and move to the next module)**

**Now I am going to show you / read to you a list of street drugs or medicines.**

** **

J1 a **In the past 12 months**, did you take any of these drugs more than once, NO YES

to get high, to feel elated, to get “a buzz” or to change your mood?

circle each drug taken:

**Stimulants:** amphetamines, "speed", crystal meth, “crank”, "rush", Dexedrine, Ritalin, diet pills.

**Cocaine:** snorting, IV, freebase, crack, "speedball".

**Narcotics:** heroin, morphine, Dilaudid, opium, Demerol, methadone, Darvon, codeine, Percodan, Vicodin, OxyContin.

**Hallucinogens:** LSD ("acid"), mescaline, peyote, psilocybin, STP, "mushrooms", “ecstasy”, MDA, MDMA.

**Phencyclidine**: PCP ("Angel Dust", "Peace Pill", “Tranq”, “Hog”), or ketamine (“Special K”).

**Inhalants:**  "glue", ethyl chloride, “rush”, nitrous oxide ("laughing gas"), amyl or butyl nitrate ("poppers").

**Cannabis:** marijuana, hashish ("hash"), THC, "pot", "grass", "weed", "reefer".

**Tranquilizers:** Quaalude, Seconal ("reds"), Valium, Xanax, Librium, Ativan, Dalmane, Halcion, barbiturates, Miltown, GHB, Roofinol, “Roofies”.

**Miscellaneous:** steroids, nonprescription sleep or diet pills. Cough Medicine? Any others?

specify the most used drug(s):

which drug(s) cause the biggest problems?: ­­­

first explore the drug causing the biggest problems and most likely to meet dependence / abuse criteria.

if meets criteria for abuse or dependence, skip to the next module. otherwise, explore the next most problematic drug.

J2 **Considering your use of (name the drug / drug class selected), in the past 12 months:**

a Have you found that you needed to use much more (name of drug / drug class selected) NO YES

to get the same effect that you did when you first started taking it?

b When you reduced or stopped using (name of drug / drug class selected), did you have NO YES

withdrawal symptoms (aches, shaking, fever, weakness, diarrhea, nausea, sweating,

heart pounding, difficulty sleeping, or feeling agitated, anxious, irritable, or depressed)?

Did you use any drug(s) to keep yourself from getting sick (withdrawal symptoms) or so

that you would feel better?

if **yes** to either, code **yes**.

c Have you often found that when you used (name of drug / drug class selected), NO YES

you ended up taking more than you thought you would?

d Have you tried to reduce or stop taking (name of drug / drug class selected) but failed? NO YES

e On the days that you used (name of drug / drug class selected), did you spend substantial NO YES

time (>2 hours), obtaining, using or recovering from the drug, or thinking about the drug?

f Did you spend less time working, enjoying hobbies, or being with family NO YES

or friends because of your drug use?

g If (nameof drug / drug class selected) caused you health or mental problems, NO YES did you still keep on using it?

| are **3** or more **J2** answers coded **yes**?  specify drug(s):**__________________________________**  ***** IF YES, SKIP J3 QUESTIONS, MOVE TO NEXT DISORDER.  “DEPENDENCE PREEMPTSABUSE” in dsm iv TR. | **NO YES ***  ***SUBSTANCE DEPENDENCE***  **CURRENT** |
| --- | --- |

**Considering your use of (name the drug class selected), in the past 12 months:**

J3 a Have you been intoxicated, high, or hungover from (name of drug / drug class selected) NO YES

more than once, when you had other responsibilities at school, at work, or at home?

Did this cause any problem?

(code **yes** only if this caused problems.)

b Have you been high or intoxicated from (name of drug / drug class selected) NO YES

more than once in any situation where you were physically at risk (for example,

driving a car, riding a motorbike, using machinery, boating, etc.)?

c Did you have legal problems more than once because of your drug use, for example, NO YES

an arrest or disorderly conduct?

d If (name of drug / drug class selected) caused problems NO YES

with your family or other people, did you still keep on using it?

| are **1** or more **J3** answers coded **yes**?  specify drug(s): **__________________________________** | **NO YES**  ***SUBSTANCE ABUSE***  **CURRENT** |
| --- | --- |

**G. PSYCHOTIC DISORDERS AND MOOD DISORDER WITH PSYCHOTIC FEATURES (MINI)**

ask for an example of each question answered positively. code **yes** only if the examples clearly show a distortion of thought or of perception or if they are not culturally appropriate.before coding, investigate whether delusions qualify as "bizarre".

delusions are "bizarre" if: clearly implausible, absurd, not understandable, and cannot derive from ordinary life experience.

hallucinations are scored "bizarre" if: a voice comments on the person's thoughts or behavior, or when two or more voices are conversing with each other. the purpose of this module is to exclude patients with psychotic disorders. this module needs experience.

Now I am going to ask you about unusual experiences that some people have. bizarre

K1 a Have you ever believed that people were spying on you, or that someone NO YES YES

was plotting against you, or trying to hurt you?

**note**: ask for examples to rule out actual stalking.

b **if yes or yes bizarre:** do you currently believe these things? NO YES YES

⮡**K6**

K2 a Have you ever believed that someone was reading your mind or could hear NO YES YES

your thoughts, or that you could actually read someone’s mind or hear what

another person was thinking?

b **if yes or yes bizarre:** do you currently believe these things? NO YES YES

⮡**K6**

K3 a Have you ever believed that someone or some force outside of yourself NO YES YES

put thoughts in your mind that were not your own, or made you act in a

way that was not your usual self? Have you ever felt that you were

possessed?

clinician:ask for examples and discount any that are not psychotic.

b **if yes or yes bizarre:** do you currently believe these things? NO YES YES

⮡**K6**

K4 a Have you ever believed that you were being sent special messages through NO YES YES

the TV, radio, internet, newspapers, books, or magazines or that a person

you did not personally know was particularly interested in you?

b **if yes or yes bizarre:** do you currently believe these things? NO YES YES

⮡**K6**

K5 a Have your relatives or friends ever considered any of your beliefs odd NO YES YES

or unusual?

interviewer**:** ask for examples. only code **yes** if the examples are **clearly**

delusional ideas not explored in questions K1 to K4, for example, SOMATIC OR RELIGIOUS

DELUSIONS OR DELUSIONS of grandiosity, JEALOUSY, guilt, ruin OR DESTITUTION, etc.

b **if yes or yes bizarre:** do they currently consider your beliefs strange? NO YES YES

K6 a Have you ever heard things other people couldn't hear, such as voices? NO YES

**if yes to voice hallucination:** Was the voice commenting on your NO YES

thoughts or behavior or did you hear two or more voices talking to each other?

b **if yes or yes bizarre to K6a:** have you heard sounds / voices in the past month? NO YES

**if yes to voice hallucination:** Was the voice commenting on your thoughts NO YES

or behavior or did you hear two or more voices talking to each other? ⮡**K8b**

K7 a Have you ever had visions when you were awake or have you ever seen things NO YES

other people couldn't see?

clinician: check to see if these are culturally inappropriate**.**

b **if yes:** have you seen these things in the past month? NO YES

**CLINICIAN'S JUDGMENT**

K8 b is the patient currently exhibiting incoherence, disorganized NO YES

speech, or marked loosening of associations?

K9 b is the patient currently exhibiting disorganized or catatonic NO YES

behavior?

K10 b are negative symptoms of schizophrenia, e.g. significant affective NO YES

flattening, poverty of speech (alogia) or an inability to initiate

or persist in goal-directed activities (avolition), prominent during

the interview?

K11 a are 1 or more « a » questions FROM K1a TO K7a coded **yes or yes bizarre**

AND IS EITHER:

major depressive episode, (current, recurrent or past)

**or**

manic or hypomanic episode, (current or past) CODED **YES**? NO YES

⮡**K13**

if no to K11 a, circle no in both ‘mood Disorder with psychotic

features’ diagnostic boxes and move to K13.

| b You told me earlier that you had period(s) when you felt (depressed/high/persistently  irritable).  Were the beliefs and experiences you just described (symptoms coded **yes** from **K1**a to **K7**a)  restricted exclusively to times when you were feeling depressed/high/irritable?  if the patient ever had a period of at least 2 weeks of having these beliefs or experiences (psychotic symptoms) when they were not depressed/high/irritable, code no to this disorder.  if the answer is no to this disorder, also circle no to K12 and move to K13 | **NO YES**  **MOOD DISORDER WITH**  *PSYCHOTIC FEATURES*  NOTE: If ‘YES’, END INTERVIEW and REFER to ALT. RESOURCES  *LIFETIME* |
| --- | --- |

| K12 a are 1 or more « b » questions FROM K1b TO K7b coded **yes or yes bizarre** AND IS EITHER:    major depressive episode, (current)  **or**  manic or hypomanic episode, (current) CODED **YES**?  if the answer is yes to this disorder (Lifetime or current), circle no to K13 and K14 and move to the next module. | **NO YES**  **MOOD DISORDER WITH**  *PSYCHOTIC FEATURES*  NOTE: If ‘YES’, END INTERVIEW and REFER to ALT. RESOURCES  *CURRENT* |
| --- | --- |

| K13 are 1 or more « b » questions FROM K1b TO K6b, coded **yes bizarre**?  **or**  are 2 or more « b » questions FROM K1b TO K10b, coded **yes** (rather than **yes bizarre**)?    and did at least two of the psychotic symptoms occur during the same 1 month period? | **NO YES**  ***PSYCHOTIC DISORDER***  **CURRENT**  **NOTE: If ‘YES’, END INTERVIEW and REFER to ALT. RESOURCES** |
| --- | --- |

| K14 is **K13** coded **yes**  **or**  are 1 or more « a » questions from K1a to K6a, coded **yes bizarre**?  **or**  are 2 or more « a » questions from K1a to K7a, coded **yes** (rather than **yes bizarre**)    and did at least two of the psychotic symptoms occur during the same 1 month period? | **NO YES**  ***PSYCHOTIC DISORDER***  **LIFETIME**  **NOTE: If ‘YES’, END INTERVIEW and REFER to ALT. RESOURCES** |
| --- | --- |

APPENDIX F. INFORMATION PAGE AND CONSENT FORM FOR WAITLIST CONTROL PARTICIPANTS


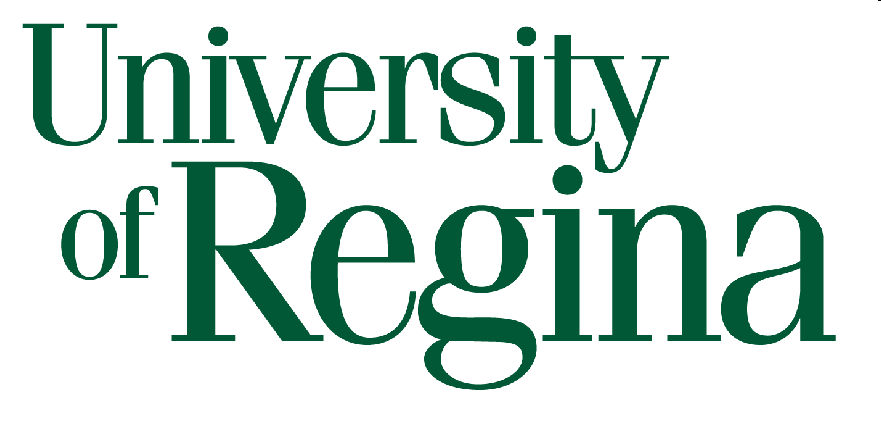

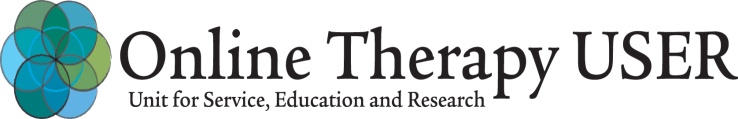


***Information Page***

Please take the time to carefully read the following information. If any of the presented information is unclear, please e-mail Nicole Pugh, at pugh200n@uregina.ca, for clarification. You may also phone her at 585-5369. If you understand and accept the terms and conditions of the Online Therapy USER program, your informed consent will be required before you can participate. The consent form is located at the end of this document. Please print a copy of the consent form for your records.

**Version Date:** April 4^th^, 2012

**Project Title:** A Randomized Controlled Trial of a Therapist-Assisted Online Therapy Program for Women with Postpartum Depression

**Principal Researcher:** Nicole Pugh, M. A., Doctoral Graduate Student (Department of Psychology, University of Regina)

**Supervisor:** Dr. Heather Hadjistavropoulos (Department of Psychology, University of Regina, and phone: 585-5133, email: heather.hadjistavropoulos@uregina.ca)

**Funded By**:The Canadian Institutes of Health Research (CIHR) and the Saskatchewan Health Research Foundation (SHRF).

**Background of Study:** Previous research has shown that therapist-guided Online Cognitive Behaviour Therapy (online-CBT) can be used to effectively treat major depression and anxiety. Such services have not been consistently available in Canada to date. Further, research has not investigated treating postpartum depression through online-CBT. The Online Therapy Unit has adapted an online-CBT depression program that was developed and tested by a team in Australia for use in Saskatchewan. We have specifically made these programs appropriate for the treatment of postpartum depression. This study will test how effective this program is in treating women with postpartum depression.

**Procedure**: To test the effectiveness of this program, participants will be randomly assigned to one of two groups: Online-CBT or a 7-10 week Pamphlet Waitlist. ***You have been randomly assigned to the Pamphlet Waitlist group.*** You will receive a pamphlet that is offered by to pregnant and postpartum women around Saskatchewan. This pamphlet provides information on postpartum depression and contact numbers and websites of postnatal support services offered in Saskatchewan. You are free to contact these or any other services during this period. You will be offered online-CBT in approximately 7-10 weeks.

**Questionnaires**: You will be asked to complete questionnaires about your levels of depression, anxiety, general and parental stress, and quality of life on two different occasions: once when the study begins and then again 7-10 weeks later. These questionnaires are located on a secure Canadian website and will take approximately ***30 minutes to complete***. After you finish reading this information page and provide consent to participate, you will be taken to the first set of questionnaires.

**Time 2 Questionnaires / Phone Call:** After you complete the first set of questionnaires, your responses will be submitted to the researcher. After **seven to** **ten weeks**, the researcher will e-mail and/or phone you to ask you to complete the same online questionnaires for a second time. This will help us to determine how you are feeling and whether your mood or other symptoms have changed over this period. At this time you will also be asked about any services you sought during the wait-period.
Following completion of the questionnaires at Time 2, you will then receive a phone call from the researcher that will involve a mini-assessment of your symptoms. This phone call will take approximately 15 minutes of your time.

**Online-CBT:** After you complete the second set of questionnaires and mini-phone assessment, if you are eligible for the treatment, you will be offered the opportunity to receive Online-CBT, which is designed to take 7-10 weeks. At this time, you will be assigned an online therapist and provided with a username and password for the onlinetherapyuser.ca website. We will go through a consent process with you again at this time.

**Storage of Clinical Information:** When you participate in this research and seek services from the Online Therapy Unit, we create a file for you. This consists of both a paper file and an online file. The paper file consists of information collected from you in the screening interview (e.g., personal information, questions about depression and anxiety). The paper file will be retained by the Online Therapy Unit. The online file consists of the e-mails you exchange in the future with your etherapist, notes your etherapists takes related to your case, and forms you complete online.

All information (whether paper or online) is kept securely for a period of seven years**,** which is consistent with standards of professional practice for psychologists in the province of Saskatchewan.

If you request access to your client file, you will be able to review the paper file and any therapy notes made on the website, as well as the emails that were exchanged between yourself and your therapist.

**Voluntary Participation:** Participation in this study is voluntary. Should you choose not to participate, or if you wish to withdraw from the study at any time after starting, you may do so without any consequences to your present or future health care. You should note that we will attempt to contact you unless we hear that you are not interested in participating.

**Confidentiality:** Only research staff who are involved in this project will know of your participation. This means that your responses to the questionnaires, along with the responses of other participants, will be accessed only by the primary researcher, Nicole Pugh, her supervisor, Dr. Hadjistavropoulos, the Online Therapy Coordinator, Marcie Nugent, or other research staff who work on this project

**Confidentiality and Internet Surveys:** There is a very small chance that your privacy may not be guaranteed by participating in this online study. Descriptions of the risks are listed below:

a. In order to protect the integrity of the study and to prevent multiple submissions from the same source, this survey will record your computer’s Internet address. All Internet Service Providers assign an identification number to every computer. This number will be temporarily stored in a file until the research is completed. After completion of the research, the primary researcher will delete the entire file. The researcher will not have access to this information, and it will not be used to identify individuals.

b. When submitting your survey answers via the Internet, there is a small possibility your information will be intercepted by unauthorized third parties using sophisticated tools. It should be noted that this rarely occurs and is a risk that can occur at anytime, not just with online surveys, when using a computer connected to the Internet.

c. Any computer connected to the Internet will store information about visited websites on the Internet browser’s history list and its disk cache. The responses to this survey are only temporarily store on your computer until you close down your browser window. In other words, after you complete and submit your survey, your computer will automatically delete this information. You may also delete the information by clearing your history list and disk cache.

d. After completion of your survey, the information will be sent directly to the survey software website. The information will then be sent to a private folder that is only accessible by the primary researcher. All responses will be downloaded at the completion of the study and then kept in a secure location by the researcher. The information will not be linked to your Internet address.

**Storage of Research Information:** Responses to questionnaires will be kept in a computer file. This file will not contain any identifying information. This file will be available to the research team; however, individual information is confidential.

• For research purposes, scores from any questionnaires you respond to will be summarized across all participants, so that individual responses will not be linked to a specific person in any publication of our results. Therefore, you as an individual will not be identifiable.

• Any details that could potentially reveal your identity will be excluded from discussions, study reports, and presentations.

• All information collected for this study will be kept in a locked office at the University of Regina and held for 5 years.

**Possible Benefits & Risks:** There are no anticipated risks associated with this online battery of questionnaires. The only cost to you will be the time required to complete the questionnaires and the mini-assessment by phone at the end of the study. This research may help women to address their postpartum depression. Further, if the treatment program is found to be effective, it may help other women who experience postpartum depression.

**Questions and Contact Information**: If you have any additional questions about the procedures of this research, please feel free to contact:

• Nicole Pugh at pugh200n@uregina.ca or call (306) 585-5369.

• Dr. Heather Hadjistavropoulos at [Heather.Hadjistavropoulos@uregina.ca](mailto:Heather.Hadjistavropoulos@uregina.ca) or call (306) 585-5133.

**Ethics Approval:** This research project has been approved on ethical grounds by the Research Ethics Boards (REBs) of the University of Regina, the University of Saskatchewan, and the Regina Qu'Appelle Health Region REBs. Any questions regarding your rights as a participant may be addressed to that committee through the University of Regina Ethics Board at (306) 585-4775 or email: research.ethics@uregina.ca. Out of town participants may call collect. Participants can also contact the University of Saskatchewan REB. Any questions regarding your rights as a participant may be addressed to that committee through the Research Ethics Office at [306-966-2975](tel:306-966-2975) or toll free at [888-966-2975](tel:888-966-2975). Participants can also contact the RQHR REB at 306-766-5451.

**Access to Study Results:** A summary of this study’s results will be posted on this website (www.onlinetherapyuser.ca) once all data have been collected and analyzed. This will likely take over a year. If you have any further questions about the research findings, please feel free to contact the Online Therapy Unit using the information listed below:

Nicole Pugh
Department of Psychology
University of Regina
3737 Wascana Parkway
Regina, SK S4S 0A2
Ph: (306) 585-5369
E-mail: pugh200n@uregina.ca

***Consent Form***

**Project Title:** A Randomized Controlled Trial of a Therapist-Assisted Online Therapy Program for Women with Postpartum Depression

I, the Participant, state the following:

1. I am 18 years of age or older.❒**Yes** ❒**No**
2. I have read the Information Page and have had any questions answered to my satisfaction. ❒**Yes** ❒**No**
3. I am aware that I can contact the research team through Nicole Pugh, at pugh200n@uregina.ca or (306) 585-5369. ❒**Yes** ❒**No**
4. This research project has been approved on ethical grounds by the Research Ethics Boards (REBs) of the University of Regina, the University of Saskatchewan, and the Regina Qu'Appelle Health Region (RQHR). I am aware that any questions regarding my rights as a participant may be addressed to that committee through the University of Regina Ethics Board at (306) 585-4775 or email: [research.ethics@uregina.ca](mailto:research.ethics@uregina.ca). Out of town participants may call collect. Participants can also contact the University of Saskatchewan REB. Any questions regarding your rights as a participant may be addressed to that committee through the Research Ethics Office at [306-966-2975](tel:306-966-2975) or toll free at [888-966-2975](tel:888-966-2975). Participants can also contact the RQHR REB at 306-766-5451.

❒Yes ❒No

1. I understand that my participation is voluntary and that I am free to withdraw at any time.

❒Yes ❒No

**Do you freely and voluntarily consent to take part in this research study? That is, do you consent to complete baseline and 10-week assessment questionnaires?**

❒ Yes ❒No


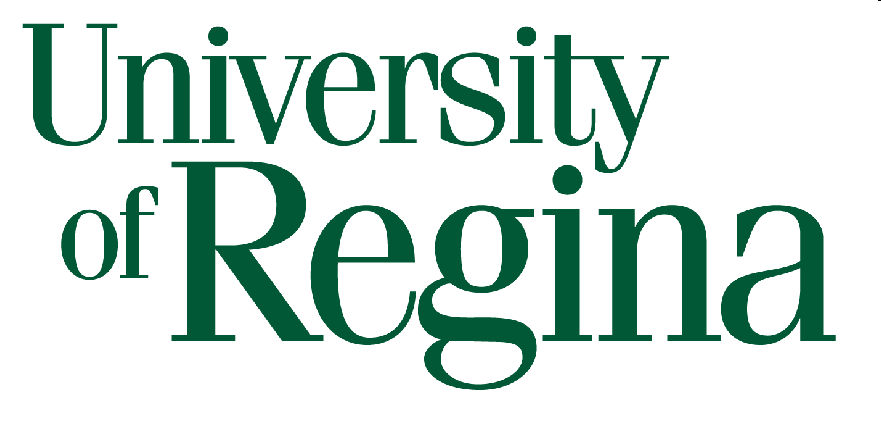
APPEDIX G. INFORMATION PAGE AND CONSENT FORM FOR THERAPIST ASSISTED ICBT (TAICBT) PARTICIPANTS


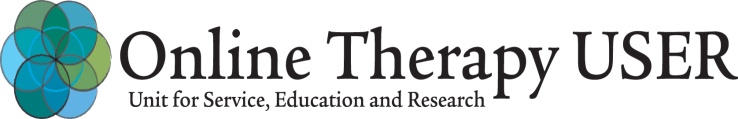


***Information Page***

Please take the time to carefully read the following information. This information includes a description of the Online Therapy USER program, the associated research project, as well as the terms and conditions of participation. If any of the presented information is unclear, please e-mail the Principal Researcher, Nicole Pugh, at pugh200n@uregina.ca, for clarification. Nicole is conducting this project as part of her dissertation research. You may also phone her at (306) 585-5369. If you understand and accept the terms and conditions of the Online Therapy USER program, your informed consent will be required before you can participate. The consent form is located at the end of this document. Please keep a copy of the consent form for your records.

**Version Date:** April 1^st^, 2012

**Project Title:** A Randomized Controlled Trial of a Therapist-Assisted Online Therapy Program for Women with Postpartum Depression

**Principal Researcher:** Nicole Pugh, M. A., Doctoral Graduate Student (Department of Psychology, University of Regina)

**Background of Study:** Previous research has shown that therapist-guided Online Cognitive Behaviour Therapy (online-CBT) can be used to effectively treat major depression and anxiety. Such services have not been consistently available in Canada to date. Further, research has not investigated treating postpartum depression through online-CBT. The Online Therapy Unit has adapted online-CBT depression programs that were developed and tested by a team in Australia for use in Saskatchewan. We have specifically made these programs appropriate for the treatment of postpartum depression. This study will test how effective this program is in treating women with postpartum depression. Because this service has not yet been offered in Saskatchewan or used with women with postpartum depression, the Online Therapy Unit will be examining the overall effectiveness of online-CBT when delivered in Saskatchewan to women with post-partum depression. The therapists in this study will be doctoral students in psychology, including Nicole Pugh, who will be under the supervision of Dr. Heather Hadjistavropoulos.

As you complete treatment, information will be collected from you for a research project aimed at understanding how many people complete the online-CBT program, who is satisfied with online-CBT program, and how effective the online-CBT program is at relieving symptoms of depression in the short- and long-term.

**Assessment Results**: Based on your responses to the telephone screening assessment, you have elevated levels of postpartum depression, and you are, therefore, eligible to take part in this online-CBT treatment. Despite meeting these eligibility criteria, you should be aware that the Online Therapy Unit assessment is not meant to take the place of best-practice, traditional, and clinically based assessments. If the information you were provided in the assessment was upsetting, or if you disagree with this information, you can call us to discuss these concerns and are also advised to contact your closest health or mental health care professional, such as your family doctor.

**Procedure:** To test this program, participants will be randomly assigned to one of two groups: Online-CBT or Pamphlet Waitlist. ***You have been randomly assigned to receive Online-CBT***. Participants assigned to this group will be asked to provide informed consent to participate in the online-CBT program and research project. If you do provide consent, you will be prompted to complete some initial questionnaires for the research project. After you have done so, you will be contacted by the researcher and provided with a Username and Password to the Online Therapy USER website. You can then begin the first module of Maternal Depression Online.

**Treatment**: Maternal Depression Online consists of **7 modules**, and it is recommended that you spend **1 week on each module**. Modules include CBT materials that are accessed and read online, materials for you to print and use offline, as well as online activities that are to be completed to help you apply the skills you are learning in daily life. This is a short-term support program, and you should be able to complete therapy in 7 weeks or less***. It is highly recommended that participants do not take any longer than 10 weeks to complete the program***. Online therapy is not intended for long-term support.

This online-CBT program is ***therapist-assisted***. When you receive your login information to this website, you will also be assigned a therapist. Your therapist will be a Doctoral Clinical Psychology student from the University of Regina (including the primary researcher Nicole Pugh) under the supervision of Dr. Heather Hadjistavropoulos, how is a registered doctoral psychologist. All student providers will receive clinical supervision from a registered professional. Prior to beginning a new module, you will be asked to send examples from the weekly exercises to your therapist through our secure website so that your therapist can review your progress and address any concerns. You may also e-mail your therapist to receive guidance and assistance with the modules and exercises. You are free to contact your therapist when it is convenient for you, and your therapist will respond to your messages once a week by the e-mail system that exists in this website over the 7 weeks of treatment. In general, therapists will respond to their clients **once a week**, however, this will vary from therapist to therapist, and yours will let you know when to expect a response. Some therapists may be able to respond to your e-mails up to twice a week depending on their availability.

You will receive the following Online-CBT program:

**Maternal Depression Online:**

| **Module 1:** | Introduction to the purpose and content of Maternal Depression Online; education about the nature of postpartum depression; learning to identify personal depression symptoms |
| --- | --- |
| **Module 2:** | Learning to monitor mood; planning enjoyable activities |
| **Module 3:** | Education about relaxation; learning specific relaxation techniques; practicing relaxation exercises |
| **Module 4 & 5:** | Education on how negative thinking contributes to depression; learning how to monitor unrealistic and unhelpful thoughts; learning how to challenge assumptions and beliefs |
| **Module 6:** | Understanding the usefulness of problem solving skills; learning how to engage in problem solving |
| **Modules 7:** | Prepare for setbacks, learning relapse prevention; Summary of Maternal Depression Online main messages, preparing to end the program |

**Outcome Assessments:** To track your progress for your own information, and also for the research study, we will be monitoring your symptoms of depression and other areas of functioning at two points:

1. Prior to starting the treatment (generally once you are done reading and filling out this consent form)
2. Ten weeks following starting the treatment.

At these two points, you will be asked to complete on-line questionnaires. Some of these questionnaires will be specific to your presenting concerns, whereas others are included to get an overall picture of your current functioning. Answering these questionnaires will take approximately ***30 minutes to complete*** each time. You are not obliged to answer any question which you find objectionable or which makes you uncomfortable. In reference to the questionnaires assessing your presenting concerns and overall functioning, you and your therapist will receive a summary of the results of these questionnaires when you fill them out during the course of treatment. The follow-up questionnaires, however, are only used for research purposes.

In addition to monitoring your progress with the online-CBT treatment, understanding your experiences with your therapist and the online-CBT program is important to us. To hear your perspective, we will be asking questions about your relationship with your therapist (e.g., how well you worked together) at the end of treatment. Open-ended questions concerning your satisfaction with the various treatment components and any areas you feel could be improved or changed will be asked when you have finished the online-CBT program.

Lastly, one month after completing the treatment, you will be contacted over the telephone by a researcher and asked about your depressive symptoms. This telephone call will take approximately 10-15 minutes to complete. You are not obliged to answer any question which you find objectionable or which makes you uncomfortable.

Your responses to the interview and questionnaires, along with the responses of other participants, will be examined by researchers to help us understand use of this online therapy program, the effectiveness of this program, and satisfaction with this program. When the researchers look at the responses they will be looking at all responses together and will not be linked to you personally

In general, your responses to the questionnaires and open-ended questions will be used to: (1) assist your therapist in providing you with better care, (2) evaluate any changes that occur as a result of your participation in online-CBT, and (3) improve online-CBT for future users.

**Possible Benefits & Challenges:** There are potential benefits and challenges associated with therapy delivered online.

| **Potential Benefits** | **Potential Challenges** |
| --- | --- |
| - You do not need to schedule an appointment with online-CBT - You avoid having to visit an office if things like transportation, stigma, or your own availability are a concern for you - You can have more control over the pace of therapy - You can access the online material from the location of your choice at your convenience for up to four weeks after the end of therapy. If you would like to continue referencing materials after four weeks, you can print off the pages - You can e-mail your therapist at any time through our secure website - You may feel more comfortable disclosing personal information online than in person - This service is provided free of charge | - Assessment and diagnosis may be more difficult when visual cues are not present - Online-CBT may require more self-motivation than other forms of therapy - Without non-verbal cues, there is a greater potential for misinterpretation of e-mail messages between you and your therapist - There is a risk for breaches of confidentiality (see below) - There is potential for technology failures that may result in messages not being received by either you or your therapist - Online-CBT is a newer form of treatment, so there has been less research conducted when compared to older forms of treatment - Online CBT is not meant to be a long- term form of therapy. - Online-CBT is not meant for use in the event of an emergency |

**Limitations of Online-CBT:** There is growing evidence that online-CBT is an effective and beneficial form of treatment for a range of mental health concerns. However, online-CBT programs are still in the early developmental phases, and as a result, there is currently less research available on its effectiveness when compared to more established treatments, such as face-to-face cognitive-behaviour therapy. It is acknowledged that there are limitations to the services provided on the Online Therapy USER website, and this form of therapy is not intended to replace face-to-face therapy. This form of therapy is also not intended for emergency services. If, during the course of therapy, you feel that the Online Therapy USER website does not meet or address your needs, or brings about other health concerns, you are advised to consult with your closest health or mental health professional.

**Alternatives to Online-CBT**: Before consenting to this type of treatment, you should consider the alternatives to online-CBT, including in-person treatment, confiding in friends or family, taking part in community programs that may be available to you, written self-help resources, visiting a family physician, or not seeking treatment at all. It is also possible that during the course of online-CBT, the therapist may determine that in-person therapy would be more suitable for you. Situations where online therapy is not appropriate include if you were to become involved in a crisis situation, if there are risks to personal safety present, if you require specialized medical treatment, and if you need support that is more long-term, interactive, or intensive. Online therapy may also not be suitable for you if you are unable to keep up with the suggested timeline of one week per module. If in-person therapy is more suitable for you, your therapist will assist you in finding appropriate in-person services in your area, with your consent.

**Pace of Treatment**: You are expected to work through the course of the program in 7 weeks or less. If you do not log onto the program for over 7 days, your therapist may call to check in with you. If you are consistently taking longer than a week per module, your therapist may speak with you about alternatives to online therapy. If you do not log into this website for 4 weeks, your participation in this program may be discontinued.

**Therapist Contact:** Your therapist will be logging into the website at least once a week. When your therapist logs in she/he will be able to review all of the messages that you send to the therapist as well as all of the forms that you submit to the therapist, such as the consent form, questionnaires, and check-in forms. Your therapist can also see the pages you have reviewed. By reviewing this information, your therapist is able to provide you with feedback, support and suggestions and also answer any questions you may have.

**Voluntary Participation & Ability to Withdraw:** Participation in online therapy is voluntary. Should you choose to not participate, or if you wish to withdraw from online therapy at any time after starting, you may do so without any consequences to your present or future health care. If you do decide to discontinue online therapy, please inform the therapist who has been assigned to you. It is important to let us know if you discontinue participation, otherwise we will continue to contact you to check in and offer you support.

**Limits of Confidentiality:** Although these circumstances are rare, there are certain limits to confidentiality that every participant must be aware of:

- If you pose an immediate threat to your life, or another individual’s life, confidentiality may be broken in order to prevent harm.
- If you disclose information suggesting that any child is at risk of abuse, the Ministry of Social Services will have to be notified.
- If you become involved in a legal case, the judge has the right to subpoena any information relevant to the legal problem.
- If you are concerned about your therapist’s professional conduct (or his/her supervisor’s), it may be necessary to release information from your file to evaluate and address this concern.
- If you request that information be released to another provider or your insurer, this request will be carried through.

**Supervision**: Students who are under supervision will need to discuss their cases with their supervisor. Students who are under supervision will need to discuss their cases with their supervisor. By using the Online Therapy User program, it is necessary for you to accept and consent to the disclosures about your case that occur between the therapist and their supervisor. In addition, your emails may be downloaded and printed by the student therapist for these purposes. Any document used for these purposes will mask your identifying data and will be shredded when no longer needed.

**Storage of Clinical Information:** When you seek services from the Online Therapy Unit, we create a file for you. This consists of both a paper file and an online file. The paper file consists of information collected from you in the screening interview (e.g., personal information, questions about depression and anxiety). The paper file will be retained by the Online Therapy Unit. The online file consists of the e-mails you exchange with your etherapist, notes your etherapists takes related to your case, and forms you complete online.

All information (whether paper or online) is kept securely for a period of seven years**,** which is consistent with standards of professional practice for psychologists in the province of Saskatchewan.

If you request access to your client file, you will be able to review the paper file and any therapy notes made on the website, as well as the emails that were exchanged between yourself and your therapist.

**Access to Client Files:** You have the right to access your client file. You may request to review or obtain your file either through verbal or written form.

Verbal requests may only be made by those who are currently obtaining Online Therapy Unit services, and you will be asked to view these records in-person. Written requests may be made by clients who are, and who are no longer, receiving services. These written communications are to be directed to the Primary Investigator, Nicole (with her contact information being included at the end of this consent form).

When you view your file, the Online Therapy Unit Coordinator will be with you in order to assist with psychological terminology and abbreviations, as well as to ensure that your record is not altered in any way. If the Coordinator is not able to answer questions, then your etherapist will be contacted to provide clarification.

If you disagree with information in the file, you can make a request in writing to add a note to your file.

**Storage of Research Information:** Responses to questionnaires will be kept in a computer file. This file will not contain any identifying information. This file will be available to the research team, however, individual information is confidential.

- For research purposes, scores from any questionnaires you respond to will be summarized across all participants, so that individual responses will not be linked to a specific person in any publication of our results. Therefore, you as an individual will not be identifiable.
- Any details that could potentially reveal your identity will be excluded from discussions, study reports, and presentations.
- All information collected for this study will be kept in a locked office at the University of Regina and held for 5 years

**Therapist Communications**: As a client, you agree not to share your therapist’s communications with anyone else unless your therapist’s written and informed consent is first obtained. You also agree not to give advice based on the therapist’s communications, or show therapist communications to others, out of context.

Copyright and Intellectual Property Material contained on the Online Therapy USER Program is copyright © University of Regina (except where otherwise indicated) or is used with permission or under license. You may download, print and reproduce this information in an unaltered form for your own personal use. All other rights are reserved. Requests for further permission to use this material should be directed to: Maureen Thompson, Research Coordinator.

**Possible Risks for Breaches of Confidentiality:** As an internet-based study/treatment, there are unique risks that may compromise your privacy that exist with any internet-based service. A description of these risks follows:

1. When submitting information to your therapist through the internet, including questionnaire answers, e-mail messages, exercises within the modules, and exercise check-ins at the beginning of modules, there is a possibility your information will be intercepted by unauthorized third parties using sophisticated tools. It should be noted that this rarely occurs, although it is a risk about which you should be advised. In order to limit this risk, the Online Therapy USER system utilizes encryption in the form of HTTPS to transmit the data both to and from yourself and your therapist. The data that is stored within the Online Therapy system, such as messages to your therapist, offline exercise examples, and responses to questionnaires, are encrypted with AES encryption. Furthermore, the system itself uses strict access controls whereby users of this system are only able to access their own information.
2. Any computer connected to the Internet will store information about visited websites on the Internet browser’s history list and its disk cache. The responses to the questionnaires are only temporarily stored on your computer until you close down your browser window. In other words, after you complete and submit your responses, your computer will automatically delete this information. You may also delete this information, as well as information about visiting the Online Therapy USER website, by clearing your history list and disk cache. Your email messages are stored in the database, and on the server, that host's the Online Therapy USER website. This sever is located in secure facilities at the University of Regina. The content of the messages are encrypted using AES encryption with 256bit key length. The Online Therapy system enforces strict access controls, and only your assigned therapist (and their supervisor if they are being supervised) can contact you, see your responses to exercises, and see your progress throughout therapy.
3. In regards to the questionnaires and questions about offline exercises that you will be filling out, after these are completed, the information you provide will be sent directly to the survey software website. The information will then be sent to a private folder that is only accessible by your therapist and researchers, however, your therapist will not have access to any questionnaires related to how you rate your therapist and the therapeutic process. All responses will be downloaded weekly and kept in a secure location by the researchers until completion of the study. The results will be stored on a secure file, and the information will not be linked to your Internet address.

**Methods Used to Protect Your Information**

In order to protect the privacy of your information while you are a user of the online CBT program, we have several precautions in place, however, you should be aware that it is not possible to safeguard against every possible risk. The precautions we use are as follows:

1. Your personal identifying information is not collected over the Internet (e.g., name, birthdate, address), and this information is not linked to your participation in the online program
2. Your login user name and password are specific to you
3. Messages exchanged within the Online Therapy program and are encrypted. This reduces the likelihood of unauthorized access to your communications.
4. The University of Regina, which hosts the Online Therapy USER website, has firewall protection to protect from external threats.
5. The access to the Online Therapy server is strictly controlled, and the server is housed in a secure environment within the University of Regina. This means that limits are in place for who has access to the server. The only people with access are the primary project developers, the server administrators, and the service administrator.

There are also various things that you can do to protect your information:

1. Use your home computer instead of a computer in a shared space, such as a library or office
2. Make sure the computer you are sending emails from is secure
3. Do not share your login information with anyone, and do not use a password that is easily guessed by others
4. Since your internet browser stores information in its memory, or disk cache, you can clean the cache after you use the computer.
   Certain browsers have "Privacy" modes that can be enabled. Once in this mode, the user's interactions are not saved to browser history and no data is stored in browser cache. Once the browser is closed or this mode is exited, there are no browser records of any of the interactions that occurred while in the "Privacy" mode. Firefox has this feature, and is, therefore, highly recommended for use with Online Therapy USER system. Browsers that do not have this mode, or users that do not use this feature, must manually purge their browser history and cache to prevent others from seeing their web interactions.
5. Enable either the firewall software that came with your operating system (e.g. Windows firewall), or install a reputable 3-rd party software, such as ZoneAlarm. Firewalls protect your computer and information from network attacks and threats.
6. Use anti-virus software to both prevent and recover from virus programs. While most anti-virus software is for purchase, there are free software options available to download. However, one must be cautious in order to avoid downloading and installing malicious software that appears to be legitimate.
7. Malware-detection software (such as Spybot: Search and Destroy) can be used to scan your computer for software and files that may be leaking your personal information to 3rd parties.

**For Your Safety**

1. We send a Physician Notification Form to your family physician or medical clinic so that your physician is aware of your participation in the Online Therapy USER program. We will also inform your physician when you complete or discontinue with the program.
2. In event of suicide risk, we will contact your family physician or medical clinic whose information you provided to us in the telephone screening interview.
3. Please inform us of any changes in your physical or mental health status that may have an impact on your ability to participate in the Online Therapy USER program.

**Emergency Situations:** In the event that the etherapist suspects you are at risk to harming yourself or others, they will contact you either by e-mail or telephone. The telephone call will be used to gain additional information about your situation. If the etherapist determines that you are at high risk, then confidentiality will need to be broken. The therapist will have to contact either: your family physician, a family member, or 911 depending on the situation.

**Multiple Therapist Roles:** It is the responsibility of the therapist to avoid holding multiple roles with clients (e.g., friend, business partner). This means that the etherapist is expected to establish and maintain a primarily professional relationship with their client. Likewise, the client is expected to respect this obligation, as well as the therapist’s ethical and professional boundaries. Due to the therapist’s limited amount of time, you may not receive an immediate e-mail response, or a response to every e-mail that you send. The etherapist will also be unable to meet requests through social networking websites (e.g., Facebook).

**Potential Therapist Unavailability:** In the event that your etherapist is unable to access their e-mail messages due to unforeseeable circumstances (e.g., sickness, injury), then Nicole Pugh or the Online Therapy USER coordinator will advise you of the situation and you will then be given options for how you would like to continue with online therapy. For example, depending upon circumstances, your therapist’s supervisor, or a replacement etherapist, may be assigned to you. If your etherapist has a planned temporary absence (e.g., holiday or work-related absence), you will be informed in advance by your etherapist and provided with options for how you would like to proceed during this time.

**Termination of Therapy:** You may withdraw from participation in the treatment at any time. Otherwise, therapy will be complete when you have completed 7 modules. Although therapy will be terminated, you will have access to the module materials, as well as to the therapist-client email system, for four weeks after therapy. If you would like to refer to the modules after these four weeks, you may do so by printing off the desired materials.

**Ethics Approval:** This research project has been approved on ethical grounds by the Research Ethics Boards (REBs) of the University of Regina, the University of Saskatchewan, and the Regina Qu'Appelle Health Region (RQHR). Any questions regarding your rights as a participant may be addressed to that committee through the University of Regina Ethics Board at (306) 585-4775 or email: [research.ethics@uregina.ca](mailto:research.ethics@uregina.ca). Out of town participants may call collect. Participants can also contact the University of Saskatchewan REB. Any questions regarding your rights as a participant may be addressed to that committee through the Research Ethics Office at 306-966-2975 or toll free at 888-966-2975. Participants can also contact the RQHR REB at 306-766-5451.

**Access to Study Results:** A summary of this study’s results will be posted on this website (**www.onlinetherapyuser.ca**) once all data have been collected and analyzed. This will likely take over a year. If you have any further questions about the research findings, please feel free to contact the Online Therapy Unit using the information listed below:

**Online Therapy Unit for Service, Education and Research
Department of Psychology**University of Regina
Regina, SK S4S 0A2
Ph: (306) 585-5369

**Technical Questions:** If you have any technical difficulty with the Online Therapy USER program, contact the Online Therapy USER coordinator, Marcie Nugent, at (306) 337-3331 who will then direct your call. You can also email her at [Marcie.Nugent@uregina.ca](mailto:Marcie.Nugent@uregina.ca).

***Consent Form***

**Project Title:** A Randomized Controlled Trial of a Therapist-Assisted Online Therapy Program for Women with Postpartum Depression

I, the Participant, state the following:

1. I am 18 years of age or older.❒**Yes** ❒**No**
2. I have read the Information Page and have had any questions answered to my satisfaction. ❒**Yes** ❒**No**
3. I am aware that I can contact the research team through Nicole Pugh, at pugh200n@uregina.ca or call (306) 585-5369. ❒**Yes** ❒**No**
4. This research project has been approved on ethical grounds by the Research Ethics Boards (REBs) of the University of Regina, the University of Saskatchewan, and the Regina Qu'Appelle Health Region (RQHR). I am aware that any questions regarding my rights as a participant may be addressed to that committee through the University of Regina Ethics Board at (306) 585-4775 or email: [research.ethics@uregina.ca](mailto:research.ethics@uregina.ca). Out of town participants may call collect. Participants can also contact the University of Saskatchewan REB. Any questions regarding your rights as a participant may be addressed to that committee through the Research Ethics Office at [306-966-2975](tel:306-966-2975) or toll free at [888-966-2975](tel:888-966-2975). Participants can also contact the RQHR REB at 306-766-5451.

❒Yes ❒No

1. I understand that my participation is voluntary and that I am free to withdraw at any time.

❒Yes ❒No

**Do you freely and voluntarily consent to take part in this research study? That is, do you consent to receive online-CBT and routinely complete outcome assessments pre-treatment and post-treatment?**

❒Yes ❒No

APPENDIX H. DEPRESSION ANXIETY STRESS SCALE (DASS-21)^[[3]](#footnote-3)^

| Please read each statement and circle a number 0, 1, 2 or 3 which indicates how much the statement applied to you *over the past week*. There are no right or wrong answers. Do not spend too much time on any statement. | | |
| --- | --- | --- |
| *The rating scale is as follows:*  0 Did not apply to me at all  1 Applied to me to some degree, or some of the time  2 Applied to me to a considerable degree, or a good part of time  3 Applied to me very much, or most of the time | | |
| 1 | I found it hard to wind down | 0 1 2 3 |
| 2 | I was aware of dryness of my mouth | 0 1 2 3 |
| 3 | I couldn't seem to experience any positive feeling at all | 0 1 2 3 |
| 4 | I experienced breathing difficulty (e.g., excessively rapid breathing, breathlessness in the absence of physical exertion) | 0 1 2 3 |
| 5 | I found it difficult to work up the initiative to do things | 0 1 2 3 |
| 6 | I tended to over-react to situations | 0 1 2 3 |
| 7 | I experienced trembling (e.g., in the hands) | 0 1 2 3 |
| 8 | I felt that I was using a lot of nervous energy | 0 1 2 3 |
| 9 | I was worried about situations in which I might panic and make a fool of myself | 0 1 2 3 |
| 10 | I felt that I had nothing to look forward to | 0 1 2 3 |
| 11 | I found myself getting agitated | 0 1 2 3 |
| 12 | I found it difficult to relax | 0 1 2 3 |
| 13 | I felt down-hearted and blue | 0 1 2 3 |
| 14 | I was intolerant of anything that kept me from getting on with what I was doing | 0 1 2 3 |
| 15 | I felt I was close to panic | 0 1 2 3 |
| 16 | I was unable to become enthusiastic about anything | 0 1 2 3 |
| 17 | I felt I wasn't worth much as a person | 0 1 2 3 |
| 18 | I felt that I was rather touchy | 0 1 2 3 |
| 19 | I was aware of the action of my heart in the absence of physical exertion (e.g., sense of heart rate increase, heart missing a beat) | 0 1 2 3 |
| 20 | I felt scared without any good reason | 0 1 2 3 |
| 21 | I felt that life was meaningless | 0 1 2 3 |

APPENDIX I. PARENTAL STRESS INDEX-SHORT FORM (PSI-SF)^[[4]](#footnote-4)^

**APPENDIX C. PARENTAL STRESS INDEX-SHORT FORM^[[5]](#footnote-5)^**

Instructions: This questionnaire contains 36 statements. Read each statement carefully. For each statement, please focus on the child you are most concerned about, and circle the response that best represents your opinion.

Select SA if you strongly agree with the statement.

Select A if you agree with the statement.

Select NS if you are not sure.

Select D if you disagree with the statement.

Select SD is you strongly disagree with the statement.

**SA = Strongly Agree A= Agree NS= Not Sure D = Disagree SD= Strongly Disagree**

| 1. I often have the feeling that I cannot handle things very well | SA | A | NS | D | SD |
| --- | --- | --- | --- | --- | --- |
| 2. I find myself giving up more of my life to meet my children’s needs than I ever expected | SA | A | NS | D | SD |
| 3. I feel trapped by my responsibilities as a parent. | SA | A | NS | D | SD |
| 4. Since having this child, I have been unable to do new and different things | SA | A | NS | D | SD |
| 5. Since having a child, I feel that I am almost never able to do things that I like to do | SA | A | NS | D | SD |
| 6. I am unhappy with the last purchase of clothing I made for myself | SA | A | NS | D | SD |
| 7. There are quite a few things that bother me about my life | SA | A | NS | D | SD |
| 8. Having a child has cause more problems than I expected in my relationship with my spouse (or male/female friend) | SA | A | NS | D | SD |
| 9. I feel alone and without any friends | SA | A | NS | D | SD |
| 10. When I go to a party, I usually expect not to enjoy myself | SA | A | NS | D | SD |
| 11. I am not as interested in people as I used to be | SA | A | NS | D | SD |
| 12. I don’t enjoy things as I used to | SA | A | NS | D | SD |
| 13. My child rarely does things for me that make me feel good | SA | A | NS | D | SD |
| 14. Sometimes I feel my child doesn’t like me and doesn’t want to be close to me | SA | A | NS | D | SD |
| 15. My child smiles at me much less than I expected | SA | A | NS | D | SD |
| 16. When I do things for my child, I get the feeling that my efforts are not appreciated very much | SA | A | NS | D | SD |
| 17. When playing, my child doesn’t often giggle or laugh | SA | A | NS | D | SD |
| 18. My child doesn’t seem to learn as quickly as most children | SA | A | NS | D | SD |
| 19. My child doesn’t seem to smile as much as most children | SA | A | NS | D | SD |
| 20. My child is not able to do as much as I expected | SA | A | NS | D | SD |
| 21. It takes a long time and it is very hard for my child to get used to new things. | SA | A | NS | D | SD |

| For the next statement, choose your response from the choices “1” to “5” below  22. I feel that I am:  1. Not very good at being a parent  2. A person who has some trouble being a parent  3. An average parent  4. A better than average parent  5. A very good parent |  |  |  |  |  |
| --- | --- | --- | --- | --- | --- |
| 23. I expected to have closer and warmer feelings for my child than I do and this bothers me | SA | A | NS | D | SD |
| 24. Sometimes my child does things that bother me just to be mean | SA | A | NS | D | SD |
| 25. My child seems to cry or fuss more often than most children | SA | A | NS | D | SD |
| 26. My child generally wakes up in a bad mood | SA | A | NS | D | SD |
| 27. I feel that my child is very moody and easily upset | SA | A | NS | D | SD |
| 28. My child does a few things which bother me a great deal | SA | A | NS | D | SD |
| 29. My child reacts very strongly when something happens that my child doesn’t like | SA | A | NS | D | SD |
| 30. My child get’s upset easily over the smallest thing | SA | A | NS | D | SD |
| 31. My child’s sleeping or eating schedule was much harder to establish than I expected | SA | A | NS | D | SD |
| For the next statement, choose your response from the choices “1” to “5” below  32. I have found that getting my child to do something or stop doing something is:   1. Much harder than I expected  2. Somewhat harder than I expected  3. About as hard as I expected  4. Somewhat easier than I expected  5. Much easier than I expected |  |  |  |  |  |
| For the next statement, choose your response form the choices “10+” to “1-3”  33. Think carefully and count the number of things which your child does that bother you  For example: dawdles, refuses to listen, overactive, cries interrupts, fights, whines etc. | 10+ | 8-9 | 6-7 | 4-5 | 1-3 |
| 34. There are some things my child does that really bother me a lot | SA | A | NS | D | SD |
| 35. My child makes more demands on me than most children | SA | A | NS | D | SD |
| 36. My child makes more demands on me than most children | SA | A | NS | D | SD |

APPENDIX J. WORLD HEALTH ORGANIZATION QUALITY OF LIFE ASSESSMENT BREF (WHOQOL-BREF)^[[6]](#footnote-6)^

**WHOQOL-BREF**

The following questions ask how you feel about your quality of life, health, or other areas of your life. I will read out each question to you, along with the response options. **Please choose the answer that appears most appropriate.** If you are unsure about which response to give to a question, the first response you think of is often the best one.

Please keep in mind your standards, hopes, pleasures and concerns. We ask that you think about your life **in the last four weeks.**

|  |  | Very poor | Poor | Neither poor nor good | Good | Very good |
| --- | --- | --- | --- | --- | --- | --- |
| 1. | How would you rate your quality of life? | 1 | 2 | 3 | 4 | 5 |

|  |  | Very dissatisfied | Dissatisfied | Neither satisfied nor dissatisfied | Satisfied | Very satisfied |
| --- | --- | --- | --- | --- | --- | --- |
| 2. | How satisfied are you with your health? | 1 | 2 | 3 | 4 | 5 |

The following questions ask about **how much** you have experienced certain things in the last four weeks.

|  |  | | Not at all | | A little | | | A moderate amount | | Very much | An extreme amount | |  |
| --- | --- | --- | --- | --- | --- | --- | --- | --- | --- | --- | --- | --- | --- |
| 3. | To what extent do you feel that physical pain prevents you from doing what you need to do? | | 5 | | 4 | | | 3 | | 2 | 1 | |  |
| 4. | How much do you need any medical treatment to function in your daily life? | | 5 | | 4 | | | 3 | | 2 | 1 | |  |
| 5. | How much do you enjoy life? | | 1 | | 2 | | | 3 | | 4 | 5 | |  |
| 6. | To what extent do you feel your life to be meaningful? | | 1 | | 2 | | | 3 | | 4 | 5 | |  |
|  | |  | | Not at all | | A little | A moderate amount | | Very much | | | Extremely | |
| 7. | | How well are you able to concentrate? | | 1 | | 2 | 3 | | 4 | | | 5 | |
| 8. | | How safe do you feel in your daily life? | | 1 | | 2 | 3 | | 4 | | | 5 | |
| 9. | | How healthy is your physical environment? | | 1 | | 2 | 3 | | 4 | | | 5 | |

The following questions ask about how completely you experience or were able to do certain things in the **last four weeks**.

|  |  | Not at all | A little | Moderately | Mostly | Completely |
| --- | --- | --- | --- | --- | --- | --- |
| 10. | Do you have enough energy for everyday life? | 1 | 2 | 3 | 4 | 5 |
| 11. | Are you able to accept your bodily appearance? | 1 | 2 | 3 | 4 | 5 |
| 12. | Have you enough money to meet your needs? | 1 | 2 | 3 | 4 | 5 |
| 13. | How available to you is the information that you need in your day-to-day life? | 1 | 2 | 3 | 4 | 5 |
| 14. | To what extent do you have the opportunity for leisure activities? | 1 | 2 | 3 | 4 | 5 |

|  |  | Very poor | Poor | Neither poor nor good | Good | Very good |
| --- | --- | --- | --- | --- | --- | --- |
| 15. | How well are you able to get around? | 1 | 2 | 3 | 4 | 5 |

|  |  | Very dissatisfied | Dissatisfied | Neither satisfied nor dissatisfied | Satisfied | Very satisfied |
| --- | --- | --- | --- | --- | --- | --- |
| 16. | How satisfied are you with your sleep? | 1 | 2 | 3 | 4 | 5 |
| 17. | How satisfied are you with your ability to perform your daily living activities? | 1 | 2 | 3 | 4 | 5 |
| 18. | How satisfied are you with your capacity for work? | 1 | 2 | 3 | 4 | 5 |
| 19. | How satisfied are you with yourself? | 1 | 2 | 3 | 4 | 5 |
| 20. | How satisfied are you with your personal relationships? | 1 | 2 | 3 | 4 | 5 |
| 21. | How satisfied are you with your sex life? | 1 | 2 | 3 | 4 | 5 |
| 22. | How satisfied are you with the support you get from your friends? | 1 | 2 | 3 | 4 | 5 |
| 23. | How satisfied are you with the conditions of your living place? | 1 | 2 | 3 | 4 | 5 |
| 24. | How satisfied are you with your access to health services? | 1 | 2 | 3 | 4 | 5 |
| 25. | How satisfied are you with your transport? | 1 | 2 | 3 | 4 | 5 |

The following question refers to how often you have felt or experienced certain things in the last four weeks.

|  |  | Never | Seldom | Quite often | Very often | Always |
| --- | --- | --- | --- | --- | --- | --- |
| 26. | How often do you have negative feelings such as blue mood, despair, anxiety, depression? | 5 | 4 | 3 | 2 | 1 |

APPENDIX K. THE THERAPEUTIC ALLIANCE QUESTIONNAIRE (TAQ)^[[7]](#footnote-7)^

There are ways that a person may feel or react in relation to another person. Consider carefully your relationship with your therapist, and then place a score in the rating column (based on the scale below), according to how strongly you agree or disagree. Please score every one.

| Strongly disagree | Disagree | Slightly Disagree | Slightly Agree | Agree | Strongly Agree |
| --- | --- | --- | --- | --- | --- |
| 1 | 2 | 3 | 4 | 5 | 6 |

|  | *Question* | Rating (0-6) |
| --- | --- | --- |
| 1 | I found I was able to rely on my therapist. | _______ |
| 2 | I felt my therapist understood those concerns that were important to me. | _______ |
| 3 | I felt my therapist was helping me to achieve my goals. | _______ |
| 4 | My therapist and I worked well together throughout the treatment program. | _______ |
| 5 | I believe that my therapist and I viewed my concerns in a similar way. | _______ |
| 6 | I felt comfortable with my therapist’s ability to guide me through the program. | _______ |
| 7 | I believe that the techniques used in the program were beneficial. | _______ |
| 8 | I developed a respect for my therapist. | _______ |
| 9 | I felt that my therapist and I were able to communicate effectively. | _______ |
| 10 | Our communications seemed to slow my treatment progress. | _______ |
| 11 | I regarded my therapist’s view about me. | _______ |
| 12 | I felt that I developed a good relationship with my therapist. | _______ |
| 13 | My therapist appeared to be competent in helping people. | _______ |
| 14 | I had meaningful communications with my therapist. | _______ |
| 15 | At times, I had fruitless exchanges with my therapist. | _______ |
| 16 | I believed the therapist respected me. | _______ |
| 17 | At times, I felt that my therapist appeared distant. | _______ |

APPENDIX L. THE TREATMENT SATISFACTION QUESTIONNAIRE (TSQ)^[[8]](#footnote-8)^

**PART A:**

Instructions: Using the scale shown in the box below, please rate ‘*how useful’* you found the various treatment components to be.

0 1 2 3 4 5 6 7

**Not at A little Somewhat Quite A lot Very much
 all so**

**Rating (0-7)**

| 1. How useful did you find the information on postpartum depression? | __________ |
| --- | --- |
| 1. How useful did you find the information regarding the available medical and psychological treatments for postpartum depression? | __________ |
| 1. How useful did you find learning about the causes of postpartum depression? | __________ |
| 1. How useful did you find developing your depression profile? | __________ |
| 1. How useful did you find the activity planning exercises? | __________ |
| 1. How useful did you find the problem solving exercises? | __________ |
| 1. How useful did you find the relaxation exercises (e.g., mindful breathing, visualization)? | __________ |
| 1. How useful did you find Cognitive Behaviour Therapy (CBT) for learning to change the way you think and feel? | __________ |
| 1. How useful did you find the thought diary for identifying unhelpful thinking styles? | __________ |
| 1. How useful was the five steps for problem solving (i.e., SOLVE)? | __________ |

**PART B:**

Instructions: Using the scale shown in the box below, please rate ‘*how much you personally liked’* the various treatment components.

0 1 2 3 4 5 6 7

**Not at A little Somewhat Quite A lot Very much**

**All So**

**Rating (0-7)**

| 1. How much did you personally like the information postpartum depression? | __________ |
| --- | --- |
| 1. How much did you personally like learning about the available medical and psychological treatments for postpartum depression? | __________ |
| 1. How much did you personally like learning about the causes of postpartum depression? | __________ |
| 1. How much did you personally like developing your depression profile? | __________ |
| 1. How much did you personally like the mood monitoring exercise? | __________ |
| 1. How much did you personally like the activity planning exercises? | __________ |
| 1. How much did you personally like the problem solving exercises? | __________ |
| 1. How much did you personally like the relaxation exercises (e.g., mindful breathing, visualization)? | __________ |
| 1. How much did you personally like Cognitive Behaviour Therapy (CBT) for learning to change the way you think and feel? | __________ |
| 1. How much did you personally like the thought diary for identifying unhelpful thinking styles? | __________ |
| 1. How much did you personally like using the five steps for problem solving (i.e., SOLVE)? | __________ |
| 1. How much did you personally like planning and engaging in social activities? | __________ |

**PART C:**

Instructions: Using the scale shown in the box below, please rate ‘*how much you think you improved’* with respect to your depression symptoms.

0 1 2 3 4 5 6 7

**Not at A little Somewhat Quite A lot Very much**

**all so**

**Rating (0-7)**

| 1. How much do you think you improved with respect to depression symptoms? | __________ |
| --- | --- |
| 1. How much do you think you improved with respect to the frequency of your depressed mood symptoms? | __________ |
| 1. How much do you think you improved with respect to the severity of your depressed mood? | __________ |
| 1. How much do you think you improved with respect to identifying your negative thoughts and feelings? | __________ |
| 1. How much do you think you improved with respect to your overall mood? | __________ |

**Part D:**

Instructions: Using the scale shown in the box below, please rate how *‘your life has changed’* in the various areas described below.

0 1 2 3 4 5 6 7

**Not at A little Somewhat Quite A lot Very much
 all so**

**Rating (0-7)**

| 1. How do you think your life has improved with respect to your participation in leisure activities? | __________ |
| --- | --- |
| 1. How do you think your life has improved with respect to your participation in family activities? | __________ |
| 1. How do you think your life has improved with respect to your participation in social activities? | __________ |
| 1. How do you think your life has improved with respect to your ability to be alone? | __________ |

**Part E:**

Instructions: Using the scale provided, please rate the following treatment evaluation questions.

0 1 2 3 4 5 6 7

**Not at A little Somewhat Quite A lot Very much
 all so**

**Rating (0-7)**

1. How much did you like the treatment program? ___________
2. How much did you enjoy communicating with your therapist? ___________

**General Comments:**

1. What was the best part of the program? ________________________________________
2. What was the worst part of the program? ________________________________________
3. How could the program be improved? __________________________________________

Overall, how much improvement do you believe occurred, after completing the treatment program? (Please Circle)

| 0 1 2 3 4 5 6 7 8 9 10  **No Improvement Most Improvement**  **at All Possible** |
| --- |

1. How many hours did you spend in total reading and re-reading the content in the Maternal Depression Online Program?

Please circle one

1-5 hours 6-11 hours 12-17 hours 17-23 hours 24-29 hours 30 hours or Over

APPENDIX M. CREDIBILITY/EXPECTANCY QUESTIONNAIRE (CEQ)^[[9]](#footnote-9)^

We would like you to indicate below how much you believe, *right now*, that the therapy you are receiving will help to reduce your anxiety. Belief usually has two aspects to it: (1) what one *thinks* will happen and (2) what one *feels* will happen. Sometimes these are similar; sometimes they are different. Please answer the questions below. In the first set, answer in terms of what you *think*. In the second set answer in terms of what you really and truly *feel*. Your therapist will not see these ratings.

**Set I**

1. At this point, how logical does the therapy offered to you seem?

| 1 | 2 | 3 | 4 | 5 | 6 | 7 | 8 | 9 |
| --- | --- | --- | --- | --- | --- | --- | --- | --- |

not at all logical somewhat logical very logical

1. At this point, how successfully do you think this treatment will be in reducing your depressive symptoms?

| 1 | 2 | 3 | 4 | 5 | 6 | 7 | 8 | 9 |
| --- | --- | --- | --- | --- | --- | --- | --- | --- |

not at all useful somewhat useful very useful

1. How confident would you be in recommending this treatment to a friend who experiences similar problems?

| 1 | 2 | 3 | 4 | 5 | 6 | 7 | 8 | 9 |
| --- | --- | --- | --- | --- | --- | --- | --- | --- |

not at all confident somewhat confident very confident

4. By the end of the therapy period, how much improvement in your depressive symptoms do you think will occur?

| 0% | 10% | 20% | 30% | 40% | 50% | 60% | 70% | 80% | 90% | 100% |
| --- | --- | --- | --- | --- | --- | --- | --- | --- | --- | --- |

**Set II**

For this set, close your eyes for a few moments, and try to identify what you really *feel* about the therapy and its likely success. Then answer the following questions.

1. At this point, how much do you really *feel* that therapy will help you to reduce your depressive symptoms?

| 1 | 2 | 3 | 4 | 5 | 6 | 7 | 8 | 9 |
| --- | --- | --- | --- | --- | --- | --- | --- | --- |

not at all somewhat very much

1. By the end of the therapy period, how much improvement in your depressive symptoms do you really *feel* will occur

| 0% | 10% | 20% | 30% | 40% | 50% | 60% | 70% | 80% | 90% | 100% |
| --- | --- | --- | --- | --- | --- | --- | --- | --- | --- | --- |

Appendix N.Physician Notification Form

**Online Therapy USER (Unit for Service Education and Research)**
Nicole Pugh, M.A.
Department of Psychology
University of Regina
3737 Wascana Parkway
Regina, SK S4S 0A2

Date: ___________________________________

Doctor Name: ___________________________________
Clinic Name: ___________________________________
Address: ___________________________________
 ___________________________________
 ___________________________________

RE: Notification of client participation in the Online Therapy USER Program

Dear Dr. ______________________________:

I am writing to inform you that your patient _____________________(health card #____________________ )
(DOB mm/dd/yyyy: _________________________) is a participant in the Online Therapy USER Program and has provided your name as a contact in case of emergency.

The Online Therapy Unit is a new online mental health unit that allows trained therapists to provide Online Cognitive Behavior Therapy (Online-CBT) to residents of Southern Saskatchewan who have difficulties with postpartum depression, major depression, generalized anxiety, and/or panic. Online-CBT involves helping clients learn how to identify and make changes to problematic thoughts (cognitions) and actions (behaviours). Clients who are screened to be appropriate for this service review educational material online and correspond with their therapist over a secure e-mail system. Online-CBT has been found to be effective and has many advantages including being more available, convenient and efficient than CBT delivered in person. If you are interested you can review our website at [www.onlinetherapyuser.ca](http://www.onlinetherapyuser.ca)

If you have any concerns about this patient participating in treatment, please call us. Reasons for non-participation might include:

1) the patient is not 18 years of age
2) the patient is at risk of suicide
3) the patient suffers from problems with alcohol or drugs
4) the patient suffers from psychotic symptoms

If you have any questions, please call the Online Therapy Unit at (306) 337-3331 or contact Dr. Heather Hadjistavropoulos at (306) 585-5133.

Sincerely,

Nicole Pugh, M. A.

Appendix O. WEEKLY MOOD MONITOR

Please rate your **average** level of anxiety, depression and fatigue. Put a number in the box below to indicate your experience. Pick the number that best corresponds to how you have been feeling over the previous week. You will continue to fill this out in the following weeks. After you submit your responses at the bottom of the page, you will be redirected to a graph. This graph will allow you to follow your progress over the course of the program.

--0--------1--------2--------3--------4--------5--------6--------7--------8--------9--------10--

| None | Mild | Moderate | Severe | Extremely Severe |
| --- | --- | --- | --- | --- |

Anxiety

Depression

Fatigue

APPENDIX P. FOLLOW-UP QUESTIONS

1. What did you find most helpful with Maternal Depression Online?

2. What did you find unhelpful with Maternal Depression Online?

3. What specific aspect of the program did you like the most?

4. What particular aspect of the program did you dislike the most?

5. How did you find the support you received once a week from your etherapist?

6. Were there factors that made it difficult to complete the Maternal Depression Online Program?

7. What information do you feel should be expanded on or added to the Maternal Depression Online program to improve the program?

8. Do you feel the program could be improved with a forum of other mothers that you could correspond with online through the program?

9. Do you think the program was as effective as in-person therapy? If yes, why?

10. Would you recommend this program to a friend struggling with postpartum depression?

11. How did participation in the program impact your ability to parent your infant?

APPENDIX Q. MOTHERFIRST AND CONTROL CONDITION PAMPHLET^[[10]](#footnote-10)^

**APPENDIX R. 10-WEEK TREATMENT QUESTIONS**
*During the 10-week period, did you seek any of the following services?*

1. Treatment from a medical doctor (e.g., general practitioner)?  Yes  No

- If yes, approximately how many appointments? _____
- Were the appointments specifically related to Postpartum Depression?  Yes  No

2. Treatment from a psychologist or mental health care worker?  Yes  No

- If yes, how many sessions did you receive? ____
- Were the sessions private or publically covered? Private  Public

3. Treatment from a support group?  Yes  No

- If yes, what was the support group for (Postpartum Depression, Mother Support group)? ______________

4. Begin a psychotropic medication (e.g., antidepressant, antianxiety)?  Yes  No

- If yes, what medication did you take?_____________
- What was the dosage of this medication?__________
- For how long did you take the medication? _________(months)

5. Begin taking naturopathic medicine (e.g., vitamins)?  Yes  No

6. Receive naturopathic or homeopathic procedures (e.g., acupuncture)?
 Yes  No

7. Access other treatments for Postpartum Depression?  Yes  No

- If yes, please explain:____________________________

8. Access the Saskatchewan Maternal Mental Health website? Yes  No

**APPENDIX S. VOLUNTEER CONFIDENTIALITY FORM**

*Oath of Confidentiality*

DECLARATION OF CONFIDENTIALITY AND NON-DISCLOSURE

I acknowledge that, in my capacity as a volunteer with the Online Therapy User Program, I will have access to certain confidential information. This information includes, but is not limited to the following: client records, research files, data books, diagrams, reports, draft publications, interviews, surveys, computer programs, and statistical information. Confidential information may be oral, written, or electronic.

I understand that all Online Therapy User volunteers, must sign a Declaration of Confidentiality and Non-Disclosure when they commence their association with the Online Therapy User program.

Under this declaration,

- I consent to keep all matters to which I am privy related to all services and projects being conducted at the agency confidential.
- I agree that I shall not disclose to any other person, firm or corporation, any confidential information relating to any clients, other than for the specific purposes required by my duties within the agency, without prior written consent from the Director or Coordinator of the Online Therapy User program.
- I also understand that I am required to notify the Coordinator immediately of any breach of my obligations or conflict of interest under this agreement which comes to my attention.

By signing this document, I confirm my understanding and acceptance of the above clause and will comply with these clauses. I also agree that my obligation to comply with the above will survive my termination of association.

Signed: ______________________________

Name (printed): ______________________________

Witness: ______________________________

Date: ____________


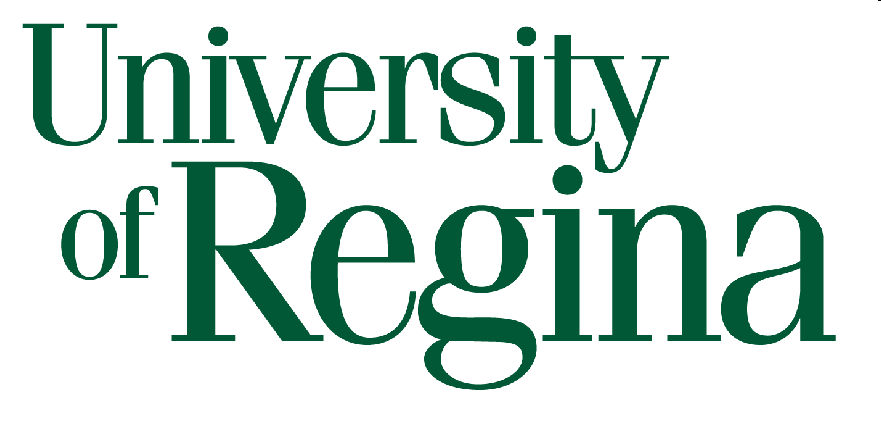
APPEDIX T. INFORMATION PAGE FOR THERAPIST ASSISTED ICBT (TAICBT) PARTICIPANTS, TIME 2


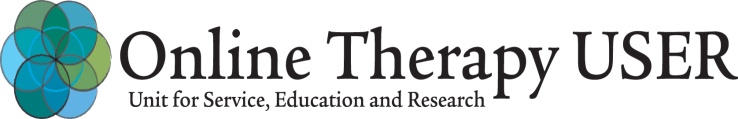


**Information Page**

**Project Title:** A  Randomized Controlled Trial of a Therapist-Assisted Online Therapy Program for Women with Postpartum Depression

**Researcher**: Nicole Pugh, M.A. PhD. Candidate in Clinical Psychology
Department of Psychology, University of Regina
Office:  (306) 585-5369
E-mail:  pugh200n@uregina.ca

**Supervisor:** Heather Hadjistavropoulos, Ph.D., R. Psych.
Department of Psychology, University of Regina
Office:  (306) 585-5133
E-mail: Heather.Hadjistavropoulos@uregina.ca

**Instructions**: It has now been approximately 10 weeks since you first completed the baseline questionnaires that assessed your symptoms of depression, stress and quality of life.  We are now asking you to complete these questionnaires for a second time. Your responses will help us to assess the effectiveness of Maternal Depression Online.

There are also several new questionnaires that will ask about your satisfaction with the program and how you found your relationship with your online therapist. Because these questions pertain directly to your therapist, she will not have access to your answers. Your name and other identifying information will not be associated with your answers during data analysis.

At the end of the survey, you will also be provided with the opportunity to provide open-ended feedback regarding your experience with Online-CBT. A number of questions are listed that you may answer. If there is anything you would like to share that is not covered in these questions, please feel free to provide it in the open comments section.

***Thank you very much for taking the time to complete these questions!***

**APPENDIX U: LIST OF ALTERNATIVE RESOURCES**

| Online Services Maternal Depression | Description |
| --- | --- |
| Postpartum Depression Support Group http://www.ppdsupportpage.com/ | A website that includes online support groups, discussion forums, and email support. |
| Postpartum Support International  http://postpartum.net/ | A website that includes information on postpartum depression, helpful resources, and free live phone sessions every week. |

| Online Services for Anxiety and Major Depression | Description |
| --- | --- |
| The Depression Center  http://www.depressioncenter.net/ | Offers tools to overcome depression as well as an online support group |
| [e-couch](http://www.ecouch.anu.edu.au/welcome)  http://www.ecouch.anu.edu.au/welcome | Provides free evidence-based information about emotional problems and teaches users strategies for preventing them |
| [Living Life to the Full](http://www.livinglifetothefull.com/index.php?section=page&page_seq=8) http://www.llttf.com/index.php?section=page&page_seq=8 | A cognitive-behavioral life skills course that aims to provide access to high quality, practical and user-friendly training in life skills |
|  |  |
| [Here toHelp](http://www.heretohelp.bc.ca/) http://www.heretohelp.bc.ca/ | A BC-based website that includes tool kits, fact sheets, and discussion forums for depression and anxiety |
| [The STEPS website](http://glasgowsteps.com/) http://glasgowsteps.com/home.php | A site that tells you all about common stress problems and gives you some ideas on how best to tackle them using CBT strategies |
|  |  |
| [Get Self Help](http://www.getselfhelp.co.uk/cbtstep1.htm) http://www.getselfhelp.co.uk/cbtstep1.htm | A mini 7-step self-help course based on CBT techniques that can be applied to many mental health problems |

| National Websites |  |
| --- | --- |
| [Canadian Mental Health Association](http://www.cmha.ca/bins/index.asp) http://www.cmha.ca/bins/index.asp | Government of Canada website with useful resources on a variety of mental health topics |
| [Centre for Addiction and Mental Health](http://www.camh.net/education/index.html)  http://www.camh.net/education/index.html | Canadian information and resources page for the treatment and management of various mental health conditions |
| [CAMIMH - Faces of mental illness](http://letstalk.bell.ca/en/perspective.php#faces)  http://letstalk.bell.ca/en/perspective.php#facesadian | Alliance of Mental Illness and Mental Health (CAMIMH) nominates five Canadians who are prepared to share their personal struggle with mental illness. |

**Mental Health Services in Southern Saskatchewan**

| **Saskatchewan HealthLine** 1-877-800-0002 is a 24-hour confidential health information and advice from a registered nurse; for the hearing impaired, call 1-888-425-4444 (TTY) | |
| --- | --- |
|  |  |
|  |  |
| The **SaskHealthline** also has a Mental Health & Addictions Program. This is staffed by Mental Health Clinicians, 24 hours a day, 7 days a week. When people call in they have the option of pressing "3" to speak with a clinician.  **Saskatchewan Health Online**  <http://www.health.gov.sk.ca/healthline-online>  An information resource on many general health topics provided as a public service for residents of Saskatchewan  **Maternal Mental Health SK Online** Information resources on topics related to maternal mental health for residents of Saskatchewan.  www.skmaternalmentalhealth.ca/ | |

**Mental Health Clinics and Alcohol and Drug Treatment services by Health Region**

| **Cypress Health Region** | |  |  |  |
| --- | --- | --- | --- | --- |
|  | |  |  |  |
|  | |  |  |  |
| The **Mental Health and Addictions** staff provides a safe, supportive and confidential environment. For more information, please call **306-778-5280** | | | |  |
|  | |  | |  |
|  | |  | |  |
| Communities Served: Cabri, Climax, Eastend, Gull Lake, Herbert, Hodgeville, Leader, Mankota, Maple Creek, Ponteix, Shaunavon, Swift Current, & Vanguard | | | |  |
|  | |  | |  |
|  | |  | |  |
| **Alcohol and Drug Treatment Services** | |  | |  |
|  | |  | |  |
|  | |  | |  |
| Addiction Services - Adult Program | | Addiction Services | |  |
| 350 Cheadle Street West | | MAPLE CREEK | |  |
| SWIFT CURRENT | | Phone: 306-662-5340 | |  |
| Phone: 306-778-5280 | |  | |  |
| **Five Hills Health Region** | | |  |  |
| **Mental Health & Addictions Services** | | |  |  |
| 455 Fairford Street East, Moose Jaw | | |  |  |
| **Main Phone:** 306-691-6464  **Parent Mentoring Program Moose Jaw**  Main Phone: 306- 692-0579 | | |  |  |
|  | | |  |  |
|  | | |  |  |
| Communities Served: Assiniboia, Central Butte, Craik, Gravelbourg, Lafleche, Moose Jaw, Mossbank, Rockglen, & Willow Bunch | | | | |
|  | | |  | |
|  | | |  | |
| **Alcohol and Drug Treatment Services** | | |  | |
|  | | |  | |
|  | | |  | |
| Mental Health and Addiction Services | | | Addiction Services | |
| 4th Floor – 455 Fairford Street East | | | Assiniboia Union Hospital | |
| MOOSE JAW | | | ASSINIBOIA | |
| Phone: 306-691-7651 | | | Phone: 306-642-9425 | |

**Sunrise Health Region**

**Part-time mental health clinics are available in some of the following facilities**

- Canora: **Canora Hospital** 306-563-5621
- Esterhazy: **St. Anthony’s Hospital** 306-745-3973 (ER; not necessarily mental health)
- Foam Lake: **Foam Lake Health Centre** 306-272-3325
- Invermay: **Invermay Health Centre** 306-593-2133
- Kamsack: **Kamsack Hospital** 306-542-2635 or **Kamsack Public Health Office** 306-542-4295 ext: 202 (Public Health Nurse)
- Melville: **St. Peter’s Hospital** 306-728-5407
- Norquay: **Norquay Health Centre** 306-594-2133
- Preeceville: **Preeceville Hospital** 306-547-2102
- Yorkton: **Yorkton Mental Health Centre**, 270 Bradbrooke Drive, 306-786-0558

| **Alcohol and Drug Treatment Services** |  |
| --- | --- |
|  |  |
|  |  |
| Addiction Services | Saul Cohen Centre |
| 345 Broadway Street West | 200 Heritage Drive |
| YORKTON | MELVILLE |
| Phone: 306-786-0520 | Phone: 728–7320 |
| Toll-free: 1-888-989-8444 |  |

**Sun Country Health Region** 

**Main Intake Office can be reached at 306-842-8665 or toll free at 1-800-216-7689**

- Arcola: Arcola Mental Health, **Arcola Health Centre** 306-455-2159
- Estevan: Estevan Mental Health Services; **St. Joseph’s Hospital** 306-637-3610
  - Estevan Family Resource Centre (306)634-7233
  - Parenting Skills Education Program, Estevan (306) 637-3620
- Kipling: Kipling Mental Health, **Kipling Memorial Health Centre** 306-736-2638
  - Public Health Nurse:  (306)736-2522
- Weyburn: **Weyburn Mental Health; Community Health Services** 306-842-8665 or Toll Free: 1-800-216-7689
  - Public Health Nurse: 306-8428618

| **Alcohol and Drug Treatment Services** |  |
| --- | --- |
|  |  |
|  |  |
| Addiction Services | Addiction Services |
| Box 420 | St. Joseph's Hospital |
| KIPLING | ESTEVAN |
| Phone: 306-736-2363 | Phone: 637-2422 or 637-2420 |
|  |  |
|  |  |
| SCHR Addiction Services |  |
| Community Supports Program |  |
| ESTEVAN |  |
| Phone: 306-637-2757 |  |

**Regina Qu’Appelle Health Region**

- Grenfell, 721 Stella Street: 306-697-4022 OR 306-697-4023
- Fort Qu’Appelle, 178 Boundary Avenue: Toll Free 1-866-367-8743
- Moosomin: 306-435-6277
- Regina Mental Health Services, 2110 Hamilton Street: 306-766-7800
- Catholic Family Services, 974 Albert Street: 306-525-0521
- Maternal Visiting (306) 765-6034
- Family Futures (306) 763-0760
- Sally Elliott YMCA Support Group (306) 757-9622 ext.242
- Regina Crisis Suicide Line: 306-525-5333
- Mobile Crisis: 306-757-0127

| **Alcohol and Drug Treatment Services** |  |
| --- | --- |
|  |  |
|  |  |
| Addiction Services - Main Office | Additions Treatment and Detox Centre |
| 2110 Hamilton Street | 1640 Victoria Street |
| REGINA | REGINA |
| Phone: 306-766-7910 | Phone: 306-766-6600 |
|  |  |
|  |  |
| Problem Gambling Services | Methadone Clinic |
| 2110 Hamilton Street | 1048 Albert Street |
| REGINA | REGINA |
| Phone: 306-766-6350 | Phone: 306-766-6350 |
|  |  |
|  |  |
| Rural Addiction Services | Rural Addiction Services |
| 721 Stella Street - Box 970 | 178 Boundary Avenue - Box 1819 |
| GRENFELL | FORT QU'APPELLE |
| Phone: 306-697-4032 | Phone: 306-332-3308 |
|  | Toll-free 1-866-367-8743 |
|  |  |
|  |  |
| Inpatient Service |  |
| Pine Lodge Treatment Centre |  |
| INDIAN HEAD |  |
| Phone: 306-695-2251 |  |

**Saskatoon Health Region**

- Humboldt Community Services, 231 Main Street: 306-682-5333
- Lanigan, Nokomis, Strasbourg, Watrous and Wynyard areas: 306-365-2099
- Rosthern Community Services, 2014 6th Street: 306-232-6001
- Wadena Integrated Hospital, 433 5th Street NE: 306-338-9950
- Wakaw Hospital, 301 1st St. N

Saskatoon:

- - Adult Services, Suite 145, 122 3rd Ave. N: 306-655-4100
  - Child and Youth Services, 715 Queen Street: 306-655-7950
  - Saskatoon Crisis Intervention Services: 306-933-6200
  - Saskatoon Postpartum Depression Support Group (306) 221-6806
  - Maternal Mental Health Program (306) 655-4250 / 966-8229

| **Alcohol and Drug Treatment Services** |  |
| --- | --- |
|  |  |
|  |  |
| Adult Residential Treatment (Calder Centre) | Brief and Social Detox |
| 2003 Arlington Ave | 201 Avenue O South |
| Saskatoon | Saskatoon |
| Phone: 306-655-4500 | Phone: 306-655-4195 |
|  |  |
|  |  |
| Community Addiction Services | Humboldt Addiction Services |
| Suite 156 Sturdy Stone Building | 640B 10th Street |
| Saskatoon | Humboldt |
| Phone: 306-655-4100 | Phone: 306-682-3249 |
|  |  |
|  |  |
| Methadone Assisted Recovery Services | McKerracher Centre |
| 345 4th Ave. South | 2302 Arlington Ave |
| Saskatoon | Saskatoon |
| Phone: 306-655-0480 | Phone: 306-655-4590 |
|  |  |
| Rehabilitation Services |  |
| 345 4th Avenue South |  |
| Saskatoon |  |
| Phone: 306-655-0440 |  |

APPENDIX V. INFORMATION PAGE AND CONSENT FORM FOR WAITLISTED PARTICIPANTS


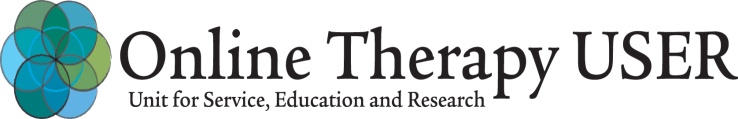


***Information Page***

Please take the time to carefully read the following information. This information includes a description of the Online Therapy USER program, the associated research project, as well as the terms and conditions of participation. If any of the presented information is unclear, please e-mail Nicole Pugh, at pugh200n@uregina.ca, for clarification. Nicole is conducting this project as part of her dissertation research. You may also phone her at (306) 585-5369. If you understand and accept the terms and conditions of the Online Therapy USER program, your informed consent will be required before you can participate. The consent form is located at the end of this document. Please keep a copy of the consent form for your records.

**Version Date:** April 1^st^, 2012

**Project Title:** A Randomized Controlled Trial of a Therapist-Assisted Online Therapy Program for Women with Postpartum Depression

**Principal Researcher:** Nicole Pugh, M. A., Doctoral Graduate Student (Department of Psychology, University of Regina)

**Background of Study:** Previous research has shown that therapist-guided Online Cognitive Behaviour Therapy (online-CBT) can be used to effectively treat major depression and anxiety. Such services have not been consistently available in Canada to date. Further, research has not investigated treating postpartum depression through online-CBT. The Online Therapy Unit has adapted online-CBT depression programs that were developed and tested by a team in Australia for use in Saskatchewan. We have specifically made these programs appropriate for the treatment of postpartum depression. This study will test how effective this program is in treating women with postpartum depression. Because this service has not yet been offered in Saskatchewan or used with women with postpartum depression, the Online Therapy Unit will be examining the overall effectiveness of online-CBT when delivered in Saskatchewan to women with post-partum depression. The therapists in this study will be doctoral students in psychology, including Nicole Pugh, who will be under the supervision of Dr. Heather Hadjistavropoulos.

As you complete treatment, information will be collected from you for a research project aimed at understanding how many people complete the online-CBT program, who is satisfied with online-CBT program, and how effective the online-CBT program is at relieving symptoms of depression in the short- and long-term.

**Assessment Results**: Based on your responses to the telephone screening assessment, you have elevated levels of postpartum depression, and you are, therefore, eligible to take part in this online-CBT treatment. Despite meeting these eligibility criteria, you should be aware that the Online Therapy Unit assessment is not meant to take the place of best-practice, traditional, and clinically based assessments. If the information you were provided in the assessment was upsetting, or if you disagree with this information, you can call us to discuss these concerns and are also advised to contact your closest health or mental health care professional, such as your family doctor.

**Procedure:** ***You were randomly assigned*** ***to the waitlist condition and are now able to receive Online-CBT***. You will be asked to provide informed consent to participate in the online-CBT program and research project. If you do provide consent, you will be contacted by the researcher and provided with a Username and Password to the Online Therapy USER website. You can then begin the first module of Maternal Depression Online.

**Treatment**: Maternal Depression Online consists of **7 modules**, and it is recommended that you spend **1 week on each module**. Modules include CBT materials that are accessed and read online, materials for you to print and use offline, as well as online activities that are to be completed to help you apply the skills you are learning in daily life. This is a short-term support program, and you should be able to complete therapy in 7 weeks or less***. It is highly recommended that participants do not take any longer than 10 weeks to complete the program***. Online therapy is not intended for long-term support.

This online-CBT program is ***therapist-assisted***. When you receive your login information to this website, you will also be assigned a therapist. Your therapist will be a Doctoral Clinical Psychology student from the University of Regina (including the primary researcher Nicole Pugh) under the supervision of Dr. Heather Hadjistavropoulos, how is a registered doctoral psychologist. All student providers will receive clinical supervision from a registered professional. Prior to beginning a new module, you will be asked to send examples from the weekly exercises to your therapist through our secure website so that your therapist can review your progress and address any concerns. You may also e-mail your therapist to receive guidance and assistance with the modules and exercises. You are free to contact your therapist when it is convenient for you, and your therapist will respond to your messages once a week by the e-mail system that exists in this website over the 7 weeks of treatment. In general, therapists will respond to their clients **once a week**, however, this will vary from therapist to therapist, and yours will let you know when to expect a response. Some therapists may be able to respond to your e-mails up to twice a week depending on their availability.

You will receive the following Online-CBT program:

**Maternal Depression Online:**

| **Module 1:** | Introduction to the purpose and content of Maternal Depression Online; education about the nature of postpartum depression; learning to identify personal depression symptoms |
| --- | --- |
| **Module 2:** | Learning to monitor mood; planning enjoyable activities |
| **Module 3:** | Education about relaxation; learning specific relaxation techniques; practicing relaxation exercises |
| **Module 4 & 5:** | Education on how negative thinking contributes to depression; learning how to monitor unrealistic and unhelpful thoughts; learning how to challenge assumptions and beliefs |
| **Module 6:** | Understanding the usefulness of problem solving skills; learning how to engage in problem solving |
| **Modules 7:** | Prepare for setbacks, learning relapse prevention; Summary of Maternal Depression Online main messages, preparing to end the program |

**Outcome Assessments:** To track your progress for your own information, and also for the research study, we will be monitoring your symptoms of depression and other areas of functioning at two points:

1. Prior to starting the treatment (generally once you are done reading and filling out this consent form)
2. Seven to ten weeks following starting the treatment.

At these two points, you will be asked to complete on-line questionnaires. Some of these questionnaires will be specific to your presenting concerns, whereas others are included to get an overall picture of your current functioning. Answering these questionnaires will take approximately ***30 minutes to complete*** each time. You are not obliged to answer any question which you find objectionable or which makes you uncomfortable. In reference to the questionnaires assessing your presenting concerns and overall functioning, you and your therapist will receive a summary of the results of these questionnaires when you fill them out during the course of treatment. The follow-up questionnaires, however, are only used for research purposes.

In addition to monitoring your progress with the online-CBT treatment, understanding your experiences with your therapist and the online-CBT program is important to us. To hear your perspective, we will be asking questions about your relationship with your therapist (e.g., how well you worked together) at the end of treatment. Open-ended questions concerning your satisfaction with the various treatment components and any areas you feel could be improved or changed will be asked when you have finished the online-CBT program.

Your responses to the interview and questionnaires, along with the responses of other participants, will be examined by researchers to help us understand use of this online therapy program, the effectiveness of this program, and satisfaction with this program. When the researchers look at the responses they will be looking at all responses together and will not be linked to you personally

In general, your responses to the questionnaires and open-ended questions will be used to: (1) assist your therapist in providing you with better care, (2) evaluate any changes that occur as a result of your participation in online-CBT, and (3) improve online-CBT for future users.

**Possible Benefits & Challenges:** There are potential benefits and challenges associated with therapy delivered online.

| **Potential Benefits** | **Potential Challenges** |
| --- | --- |
| - You do not need to schedule an appointment with online-CBT - You avoid having to visit an office if things like transportation, stigma, or your own availability are a concern for you - You can have more control over the pace of therapy - You can access the online material from the location of your choice at your convenience for up to four weeks after the end of therapy. If you would like to continue referencing materials after four weeks, you can print off the pages - You can e-mail your therapist at any time through our secure website - You may feel more comfortable disclosing personal information online than in person - This service is provided free of charge | - Assessment and diagnosis may be more difficult when visual cues are not present - Online-CBT may require more self-motivation than other forms of therapy - Without non-verbal cues, there is a greater potential for misinterpretation of e-mail messages between you and your therapist - There is a risk for breaches of confidentiality (see below) - There is potential for technology failures that may result in messages not being received by either you or your therapist - Online-CBT is a newer form of treatment, so there has been less research conducted when compared to older forms of treatment - Online CBT is not meant to be a long- term form of therapy. - Online-CBT is not meant for use in the event of an emergency |

**Limitations of Online-CBT:** There is growing evidence that online-CBT is an effective and beneficial form of treatment for a range of mental health concerns. However, online-CBT programs are still in the early developmental phases, and as a result, there is currently less research available on its effectiveness when compared to more established treatments, such as face-to-face cognitive-behaviour therapy. It is acknowledged that there are limitations to the services provided on the Online Therapy USER website, and this form of therapy is not intended to replace face-to-face therapy. This form of therapy is also not intended for emergency services. If, during the course of therapy, you feel that the Online Therapy USER website does not meet or address your needs, or brings about other health concerns, you are advised to consult with your closest health or mental health professional.

**Alternatives to Online-CBT**: Before consenting to this type of treatment, you should consider the alternatives to online-CBT, including in-person treatment, confiding in friends or family, taking part in community programs that may be available to you, written self-help resources, visiting a family physician, or not seeking treatment at all. It is also possible that during the course of online-CBT, the therapist may determine that in-person therapy would be more suitable for you. Situations where online therapy is not appropriate include if you were to become involved in a crisis situation, if there are risks to personal safety present, if you require specialized medical treatment, and if you need support that is more long-term, interactive, or intensive. Online therapy may also not be suitable for you if you are unable to keep up with the suggested timeline of one week per module. If in-person therapy is more suitable for you, your therapist will assist you in finding appropriate in-person services in your area, with your consent.

**Pace of Treatment**: You are expected to work through the course of the program in 7 weeks or less. If you do not log onto the program for over 7 days, your therapist may call to check in with you. If you are consistently taking longer than a week per module, your therapist may speak with you about alternatives to online therapy. If you do not log into this website for 4 weeks, your participation in this program may be discontinued.

**Therapist Contact:** Your therapist will be logging into the website at least once a week. When your therapist logs in she/he will be able to review all of the messages that you send to the therapist as well as all of the forms that you submit to the therapist, such as the consent form, questionnaires, and check-in forms. Your therapist can also see the pages you have reviewed. By reviewing this information, your therapist is able to provide you with feedback, support and suggestions and also answer any questions you may have.

**Voluntary Participation & Ability to Withdraw:** Participation in online therapy is voluntary. Should you choose to not participate, or if you wish to withdraw from online therapy at any time after starting, you may do so without any consequences to your present or future health care. If you do decide to discontinue online therapy, please inform the therapist who has been assigned to you. It is important to let us know if you discontinue participation, otherwise we will continue to contact you to check in and offer you support.

**Limits of Confidentiality:** Although these circumstances are rare, there are certain limits to confidentiality that every participant must be aware of:

- If you pose an immediate threat to your life, or another individual’s life, confidentiality may be broken in order to prevent harm.
- If you disclose information suggesting that any child is at risk of abuse, the Ministry of Social Services will have to be notified.
- If you become involved in a legal case, the judge has the right to subpoena any information relevant to the legal problem.
- If you are concerned about your therapist’s professional conduct (or his/her supervisor’s), it may be necessary to release information from your file to evaluate and address this concern.
- If you request that information be released to another provider or your insurer, this request will be carried through.

**Supervision**: Students who are under supervision will need to discuss their cases with their supervisor. Students who are under supervision will need to discuss their cases with their supervisor. By using the Online Therapy User program, it is necessary for you to accept and consent to the disclosures about your case that occur between the therapist and their supervisor. In addition, your emails may be downloaded and printed by the student therapist for these purposes. Any document used for these purposes will mask your identifying data and will be shredded when no longer needed.

**Storage of Clinical Information:** When you seek services from the Online Therapy Unit, we create a file for you. This consists of both a paper file and an online file. The paper file consists of information collected from you in the screening interview (e.g., personal information, questions about depression and anxiety). The paper file will be retained by the Online Therapy Unit. The online file consists of the e-mails you exchange with your etherapist, notes your etherapists takes related to your case, and forms you complete online.

All information (whether paper or online) is kept securely for a period of seven years**,** which is consistent with standards of professional practice for psychologists in the province of Saskatchewan.

If you request access to your client file, you will be able to review the paper file and any therapy notes made on the website, as well as the emails that were exchanged between yourself and your therapist.

**Access to Client Files:** You have the right to access your client file. You may request to review or obtain your file either through verbal or written form.

Verbal requests may only be made by those who are currently obtaining Online Therapy Unit services, and you will be asked to view these records in-person. Written requests may be made by clients who are, and who are no longer, receiving services. These written communications are to be directed to the Primary Investigator, Nicole (with her contact information being included at the end of this consent form).

When you view your file, the Online Therapy Unit Coordinator will be with you in order to assist with psychological terminology and abbreviations, as well as to ensure that your record is not altered in any way. If the Coordinator is not able to answer questions, then your etherapist will be contacted to provide clarification.

If you disagree with information in the file, you can make a request in writing to add a note to your file.

**Storage of Research Information:** Responses to questionnaires will be kept in a computer file. This file will not contain any identifying information. This file will be available to the research team, however, individual information is confidential.

- For research purposes, scores from any questionnaires you respond to will be summarized across all participants, so that individual responses will not be linked to a specific person in any publication of our results. Therefore, you as an individual will not be identifiable.
- Any details that could potentially reveal your identity will be excluded from discussions, study reports, and presentations.
- All information collected for this study will be kept in a locked office at the University of Regina and held for 5 years

**Therapist Communications**: As a client, you agree not to share your therapist’s communications with anyone else unless your therapist’s written and informed consent is first obtained. You also agree not to give advice based on the therapist’s communications, or show therapist communications to others, out of context.

Copyright and Intellectual Property Material contained on the Online Therapy USER Program is copyright © University of Regina (except where otherwise indicated) or is used with permission or under license. You may download, print and reproduce this information in an unaltered form for your own personal use. All other rights are reserved. Requests for further permission to use this material should be directed to: Maureen Thompson, Research Coordinator.

**Possible Risks for Breaches of Confidentiality:** As an internet-based study/treatment, there are unique risks that may compromise your privacy that exist with any internet-based service. A description of these risks follows:

1. When submitting information to your therapist through the internet, including questionnaire answers, e-mail messages, exercises within the modules, and exercise check-ins at the beginning of modules, there is a possibility your information will be intercepted by unauthorized third parties using sophisticated tools. It should be noted that this rarely occurs, although it is a risk about which you should be advised. In order to limit this risk, the Online Therapy USER system utilizes encryption in the form of HTTPS to transmit the data both to and from yourself and your therapist. The data that is stored within the Online Therapy system, such as messages to your therapist, offline exercise examples, and responses to questionnaires, are encrypted with AES encryption. Furthermore, the system itself uses strict access controls whereby users of this system are only able to access their own information.
2. Any computer connected to the Internet will store information about visited websites on the Internet browser’s history list and its disk cache. The responses to the questionnaires are only temporarily stored on your computer until you close down your browser window. In other words, after you complete and submit your responses, your computer will automatically delete this information. You may also delete this information, as well as information about visiting the Online Therapy USER website, by clearing your history list and disk cache. Your email messages are stored in the database, and on the server, that host's the Online Therapy USER website. This sever is located in secure facilities at the University of Regina. The content of the messages are encrypted using AES encryption with 256bit key length. The Online Therapy system enforces strict access controls, and only your assigned therapist (and their supervisor if they are being supervised) can contact you, see your responses to exercises, and see your progress throughout therapy.
3. In regards to the questionnaires and questions about offline exercises that you will be filling out, after these are completed, the information you provide will be sent directly to the survey software website. The information will then be sent to a private folder that is only accessible by your therapist and researchers, however, your therapist will not have access to any questionnaires related to how you rate your therapist and the therapeutic process. All responses will be downloaded weekly and kept in a secure location by the researchers until completion of the study. The results will be stored on a secure file, and the information will not be linked to your Internet address.

**Methods Used to Protect Your Information**

In order to protect the privacy of your information while you are a user of the online CBT program, we have several precautions in place, however, you should be aware that it is not possible to safeguard against every possible risk. The precautions we use are as follows:

1. Your personal identifying information is not collected over the Internet (e.g., name, birthdate, address), and this information is not linked to your participation in the online program
2. Your login user name and password are specific to you
3. Messages exchanged within the Online Therapy program and are encrypted. This reduces the likelihood of unauthorized access to your communications.
4. The University of Regina, which hosts the Online Therapy USER website, has firewall protection to protect from external threats.
5. The access to the Online Therapy server is strictly controlled, and the server is housed in a secure environment within the University of Regina. This means that limits are in place for who has access to the server. The only people with access are the primary project developers, the server administrators, and the service administrator.

There are also various things that you can do to protect your information:

1. Use your home computer instead of a computer in a shared space, such as a library or office
2. Make sure the computer you are sending emails from is secure
3. Do not share your login information with anyone, and do not use a password that is easily guessed by others
4. Since your internet browser stores information in its memory, or disk cache, you can clean the cache after you use the computer.
   Certain browsers have "Privacy" modes that can be enabled. Once in this mode, the user's interactions are not saved to browser history and no data is stored in browser cache. Once the browser is closed or this mode is exited, there are no browser records of any of the interactions that occurred while in the "Privacy" mode. Firefox has this feature, and is, therefore, highly recommended for use with Online Therapy USER system. Browsers that do not have this mode, or users that do not use this feature, must manually purge their browser history and cache to prevent others from seeing their web interactions.
5. Enable either the firewall software that came with your operating system (e.g. Windows firewall), or install a reputable 3-rd party software, such as ZoneAlarm. Firewalls protect your computer and information from network attacks and threats.
6. Use anti-virus software to both prevent and recover from virus programs. While most anti-virus software is for purchase, there are free software options available to download. However, one must be cautious in order to avoid downloading and installing malicious software that appears to be legitimate.
7. Malware-detection software (such as Spybot: Search and Destroy) can be used to scan your computer for software and files that may be leaking your personal information to 3rd parties.

**For Your Safety**

1. We send a Physician Notification Form to your family physician or medical clinic so that your physician is aware of your participation in the Online Therapy USER program. We will also inform your physician when you complete or discontinue with the program.
2. In event of suicide risk, we will contact your family physician or medical clinic whose information you provided to us in the telephone screening interview.
3. Please inform us of any changes in your physical or mental health status that may have an impact on your ability to participate in the Online Therapy USER program.

**Emergency Situations:** In the event that the etherapist suspects you are at risk to harming yourself or others, they will contact you either by e-mail or telephone. The telephone call will be used to gain additional information about your situation. If the etherapist determines that you are at high risk, then confidentiality will need to be broken. The therapist will have to contact either: your family physician, a family member, or 911 depending on the situation.

**Multiple Therapist Roles:** It is the responsibility of the therapist to avoid holding multiple roles with clients (e.g., friend, business partner). This means that the etherapist is expected to establish and maintain a primarily professional relationship with their client. Likewise, the client is expected to respect this obligation, as well as the therapist’s ethical and professional boundaries. Due to the therapist’s limited amount of time, you may not receive an immediate e-mail response, or a response to every e-mail that you send. The etherapist will also be unable to meet requests through social networking websites (e.g., Facebook).

**Potential Therapist Unavailability:** In the event that your etherapist is unable to access their e-mail messages due to unforeseeable circumstances (e.g., sickness, injury), then Nicole Pugh or the Online Therapy USER coordinator will advise you of the situation and you will then be given options for how you would like to continue with online therapy. For example, depending upon circumstances, your therapist’s supervisor, or a replacement etherapist, may be assigned to you. If your etherapist has a planned temporary absence (e.g., holiday or work-related absence), you will be informed in advance by your etherapist and provided with options for how you would like to proceed during this time.

**Termination of Therapy:** You may withdraw from participation in the treatment at any time. Otherwise, therapy will be complete when you have completed 7 modules. Although therapy will be terminated, you will have access to the module materials, as well as to the therapist-client email system, for four weeks after therapy. If you would like to refer to the modules after these four weeks, you may do so by printing off the desired materials.

**Ethics Approval:** This research project has been approved on ethical grounds by the Research Ethics Boards (REBs) of the University of Regina, the University of Saskatchewan, and the Regina Qu'Appelle Health Region (RQHR). Any questions regarding your rights as a participant may be addressed to that committee through the University of Regina Ethics Board at (306) 585-4775 or email: [research.ethics@uregina.ca](mailto:research.ethics@uregina.ca). Out of town participants may call collect. Participants can also contact the University of Saskatchewan REB. Any questions regarding your rights as a participant may be addressed to that committee through the Research Ethics Office at 306-966-2975 or toll free at 888-966-2975. Participants can also contact the RQHR REB at 306-766-5451.

**Access to Study Results:** A summary of this study’s results will be posted on this website (**www.onlinetherapyuser.ca**) once all data have been collected and analyzed. This will likely take over a year. If you have any further questions about the research findings, please feel free to contact the Online Therapy Unit using the information listed below:

**Online Therapy Unit for Service, Education and Research
Department of Psychology**University of Regina
Regina, SK S4S 0A2
Ph: (306) 585-5369

**Technical Questions:** If you have any technical difficulty with the Online Therapy USER program, contact the Online Therapy USER coordinator, Marcie Nugent, at (306) 337-3331 who will then direct your call. You can also email her at [Marcie.Nugent@uregina.ca](mailto:Marcie.Nugent@uregina.ca).

***Consent Form***

**Project Title:** A Randomized Controlled Trial of a Therapist-Assisted Online Therapy Program for Women with Postpartum Depression

I, the Participant, state the following:

1. I am 18 years of age or older.❒**Yes** ❒**No**
2. I have read the Information Page and have had any questions answered to my satisfaction. ❒**Yes** ❒**No**
3. I am aware that I can contact the research team through Nicole Pugh, at pugh200n@uregina.ca or call (306) 585-5369. ❒**Yes** ❒**No**
4. This research project has been approved on ethical grounds by the Research Ethics Boards (REBs) of the University of Regina, the University of Saskatchewan, and the Regina Qu'Appelle Health Region (RQHR). I am aware that any questions regarding my rights as a participant may be addressed to that committee through the University of Regina Ethics Board at (306) 585-4775 or email: [research.ethics@uregina.ca](mailto:research.ethics@uregina.ca). Out of town participants may call collect. Participants can also contact the University of Saskatchewan REB. Any questions regarding your rights as a participant may be addressed to that committee through the Research Ethics Office at [306-966-2975](tel:306-966-2975) or toll free at [888-966-2975](tel:888-966-2975). Participants can also contact the RQHR REB at 306-766-5451.

❒Yes ❒No

1. I understand that my participation is voluntary and that I am free to withdraw at any time.

❒Yes ❒No

**Do you freely and voluntarily consent to take part in this research study? That is, do you consent to receive online-CBT and routinely complete outcome assessments pre-treatment and post-treatment?**

❒Yes ❒No

**APPDENDIX W. PROTOCOL TO ADDRESS A CLIENT WHO IS AT RISK OF SUICIDE**

During the screening process, potential clients are asked a series of suicidality questions. If an individual being screened endorses items that suggest suicidal ideation, the screener must adhere to the following steps:

1) Establish rapport with the client and communicate to her willingness to help. Listen to the client in a concerned and empathetic manner, while also assessing their suicidal potential. The questions to obtain the individual’s suicidal potential are located within the Full Screen Interview under the *Suicidality* section in the M.I.N.I. (see Appendix E, Module B). The screener MUST ask all of these questions to adequately assess the individual’s suicide risk. These questions include information on the frequency, intensity, and duration of these thoughts, the specifics of a potential suicide plan, and the individual’s history around suicidality.

2) If the individual receiving screening does have a suicide plan, the screener will ensure that they have all the information about this plan using the SLAP acronym, which includes information regarding the following factors:

- Specificity of the plan: The more specific the plan, the greater the risk of suicide.
- Lethality of the method: The greater the lethality, the higher the risk of suicide.
- Inquire about the general method and how method will be employed.
- Availability of the method.
- Proximity of social or helping resources: The further the client is from support the greater the risk.

Once the screener has assessed the potential client’s suicide risk, they will decide on an appropriate course of action based on the client’s risk of suicide. They should seek supervision as soon as possible for this situation. They must also document the screening, as well as the actions they took.

If the client is at ***high risk*** for suicide (e.g., specific plan, lethal and available method, resources are limited), then the screener should encourage this person to seek immediate medical attention from their physician. If their physician is unavailable, then they should be directed to the emergency room of the nearest hospital. This potential client should be asked to contact someone they feel comfortable speaking with to assist them in going to the doctor or to the hospital.

If the plan appears immediate, the screener is to call 911. The screener should also let the potential client know that they will need to contact their physician to ensure that he or she is aware of their client’s situation, and so that they can monitor the client’s status.

In regards to ***medium suicide risk***, the screener will encourage the client to seek assistance from their local mental health clinic. The screener must also provide the potential client with the mental health clinic’s contact information (i.e., phone number, address). They may also consider contacting the family physician to make him or her aware of the client’s difficulties. If the screener is going to contact the physician, they must let this potential client know.

In the case of ***low risk*** suicidality, the screener should seek a supervisor out in order to determine if the risk of suicide is sufficiently low for the client to participate in online therapy, or if the client should be encouraged to seek in person services. Actions will be taken based on this decision.

**APPENDIX X. TELEPHONE SCRIPT (USED BEFORE PRE-SCREENING INTERVIEW)**

**Maternal Depression Online**

1. Before we start the screening process, I will first provide you with some background to Maternal Depression Online and this research project.
2. Maternal Depression Online is based on **Cognitive Behaviour Therapy (CBT)** which is a structured psychological therapy. CBT has been found to be effective for treating general depression, anxiety, and other mental health concerns.
3. Online CBT gives the **same** information that you would receive in **in-person therapy** but is **modified** to be provided in an **online format**.
4. It is **flexible**, in that you can review the material at a time that works for you without having to make an appointment with your therapist.
5. Online CBT is **Therapist Assisted**, in that a therapist checks in with you **once a week** on a set day to give **feedback through email** over our website.
6. We are offering one particular program for women who experience either minor or major depression and who have a child under the age of one. In this program, there are **7 modules** that include information about maternal depression, reviews activity planning, relaxation techniques, how to monitor thoughts and challenge them, problem solving skills, and other coping strategies. Typically, clients **complete one module per week** – so that the program lasts **7 weeks**.
7. Although research has found online CBT effective for the treatment of depression in women who are not new mothers, this particular therapy program **has never been tested for women who are new mothers (maternal depression).** To see if it is effective, I am studying this therapy for my dissertation research. Women who are interested and eligible in participating will be **randomly assigned** to either receive **online therapy right away** or will be placed on a **10-week waitlist** and be offered treatment at that time. We will randomly assign you after you complete a telephone pre-screening and assessment that will determine if you’re eligible to take part.
8. Both groups will complete **questionnaires** **before and after 10 weeks** to see if the therapy helps to improve their symptoms. We’re also interested in understanding participants’ experiences with the therapy, so at the end there will some questions that you can respond to that will help us to improve this service for other seniors who use this therapy in the future.
9. There are **several steps** involved in this study.
   1. First, we would have you complete a **pre-screening telephone interview.** This should take approximately **15 minutes**.
   2. Second, there is a **full-screening telephone interview**. This is designed to help us make sure that the therapy program we are providing is appropriate for your concerns. This interview can take anywhere from **30-60 minutes**.
   3. Once we determine that you are able to participate, treatment will take between 7 and 10 weeks, or the waitlist will take 10 weeks.
   4. We will also contact you four weeks after you complete the treatment to see how you are doing.

***So now that you know a bit about the formatting of the program and the research involved, does this still sound like something that you would be interested in?***

**APPDENDIX Y. PROTOCOL TO ADDRESS A CLIENT WHO INDICATES PARTNER ABUSE**

During the screening process, potential clients are asked if they have been or are currently the victim of abuse. If an individual indicates that she is currently the victim of abuse, the screener must adhere to the following steps:

1) Establish rapport with the client and communicate to her willingness to help. Listen to the client in a concerned and empathetic manner, while also assessing the nature of abuse. The screener should inquire about the frequency, intensity, and duration of the abuse and if she is at an immediate risk of harm. If the client indicates that her safety is an immediate concern, the screener should ask the following questions^[[11]](#footnote-11)^:

- Are you safe right now?
- Do you want me to call the police for you?
- If we get disconnected, I’m going to call the police, okay?
- Do you have a personal protection order in place?
- Where can you go that you would feel safer?
- Can I give you the number of the local domestic violence shelter program? (see Appendix Z).

2) The screener should also inquire if a child is being abused or neglected. If a client indicates that a child is being abused and/or neglected, the screener must inform the participant that confidentiality will be broken. The screener will report the abuse/neglect to local authorities.

If child abuse or neglect is reported, the screener should report this information by to a local Ministry of Social Services, the First Nations Child and Family Services Agency, a Child Protection Worker, a Police Officer, or contact 9-11.

When reporting a case of child abuse or neglect, the screener should include^[[12]](#footnote-12)^:

- - Their name and telephone number
  - Any immediate concerns about the child’s safety
  - The location of the child
  - The child’s name and age
  - Information regarding the situation of abuse and/or neglect
  - Any other relevant information

If the screener has any reason to believe that a child is being abused or neglected, they have a legal duty to report this information. The screener is not expected to determine if a child is being abused or neglected, rather, a trained social worker will make that determination. Members of the public are simply obligated by the law to report suspected abuse or neglect. If an individual does not report a suspicion of abuse or neglect, that person could be fined up to $25,000, receive a jail term of up to 24 months, or acquire both a fine and a jail term^[[13]](#footnote-13)^.

**APPENDIX Z: LIST OF SHELTERS IN SASKACHEWAN**

Estevan:

- Envision Counselling and Support Centre (306) 637-4004

Fort Qu'Appelle

- Qu'Appelle Haven Safe Shelter (306) 332-6881

Hudson Bay

- Hudson Bay and District Crisis Centre (306) 865-3391

Kindersley

- West Central Crisis and Family Support Centre (306) 463-6655

La Ronge

- Piwapan Women's Centre (306) 425-3900

Lloydminster

- Lloydminster Interval Home (780) 875-0966
- Dol-Mar Manor (2nd Stage) (780) 808-5282

Meadow Lake

- Waskoosis Safe Shelter (306) 236-5570

Melfort

- North East Crisis Intervention Centre (306) 752-9464

Moose Jaw

- Moose Jaw Transition House (306) 693-6511

North Battleford

- Battlefords Interval House (306) 445-2750

Prince Albert

- Children's Haven (306) 922-4454
- PA Safe Shelter for Women (306) 764-7233
- PA Safe Shelter - 2nd Stage Housing 306-764-7233

Regina

- Isabel Johnson Shelter, YWCA of Regina (306) 525-2141
- Regina Transition House (306) 757-2096
- Sofia House - 2nd Stage Housing (306) 565-2537
- Wichihik Iskwewak Safe House (306) 543-0493

Saskatoon

- Saskatoon Interval House (306) 244-0185
- YWCA of Saskatoon (306) 244-2844
- Adelle House - 2nd Stage Housing (306) 668-2761
- Crisis Nursery (306) 242-2433
- Family Healing Circle Lodge (306) 653-3900

Swift Current

- Southwest Safe Shelter (306) 778-3386
- Genesis House - 2nd Stage Housing scroll down (306) 778-3386

Weyburn

- Envision Counselling And Support Centre (306) 842-8821

Yorkton

- Project Safe Haven (306) 782-0676
- Shelwin House (306) 783-7233

1. From “Detection of Postnatal Depression: Development of the 10-Item Edinburgh Postnatal Depression Scale,” by J. L. Cox, J. M. Holden, & R. Sagovsky, 1987. In *The British Journal of Psychiatry, 150,* 782-786. [↑](#footnote-ref-1)
2. From “The Mini-International Neuropsychiatric Interview (M.I.N.I): The Development and Validation of a Structured Diagnositc Psychiatric Interview for DSM-IV and ICD-10”, by Sheehan, D. V., Lecrubier, Y., Sheehan, H., Amorim, P., Janavs, J., Weiller, E.,…Dunbar, G. C. , 1998. In *Journal of Clinical Psychiatry, 59,* 22-33. [↑](#footnote-ref-2)
3. From “The Structure of Negative Emotional States: Comparison of the Depression Anxiety Stress Scales (DASS) with the Beck Depression and Anxiety Inventories,” by, P. F. Lovibond, & S. H. Lovibond, 1995. In *Behaviour Research and Therapy, 33*, 335-343. [↑](#footnote-ref-3)
4. *The Parenting Stress Index: Short Form (PSI/SF).* Available from http://www4.parinc.com/Products/Product.aspx?ProductID=PSI-SF [↑](#footnote-ref-4)
5. *The Parenting Stress Index: Short Form (PSI/SF).* Available from <http://www4.parinc.com/Products/Product.aspx?ProductID=PSI-SF> [↑](#footnote-ref-5)
6. From “Development of the World Health Organization WHOQOL-BREF Quality of Life Assessment,” by The WHOQOL Group, 1998.*PsychologicalMedicine, 28,*551-558. [↑](#footnote-ref-6)
7. From “The Revised Helping Alliance Questionnaire (HAq-II),” by L. Luborsky, J. P. Barber, L.Siqueland, S. Johnstone, L. M.Najavits, A. Frank, & D. Daley, 1996. *Journal of Psychotherapy Practice and Research, 5,* 260-271. [↑](#footnote-ref-7)
8. Adapted from “Patient Satisfaction with Behavioural Treatments for Panic Disorder with Agoraphobia”, by B. J.Cox, K. D. Fergus, & R. P. Swinson, 1994. *Journal of Anxiety Disorders, 8*, 193-206. [↑](#footnote-ref-8)
9. From “Psychometric Properties of the Credibility/Expectancy Questionnaire,”by G.J. Devilly& T. Borkovec, 2000.In *Journal of Behavior Therapy and Experimental Psychiatry, 31*, 73-86. [↑](#footnote-ref-9)
10. From *Maternal Mental Health Strategy: Building Capacity in Saskatchewan,* by MotherFirst Working Group, 2010*.*Copyright 2010 by MotherFirst Working Group. Reprinted with permission. Retrieved from <https://sites.google.com/site/maternalmentalhealthsk/> [↑](#footnote-ref-10)
11. Questions revised from Suillivan, C. M., & Cain, D. (2002). Ethical and safety considerations when obtaining information from or about battered women for research purposes. *Journal of Interpersonal Violence, 19,* 603-620. doi: 10.1177/0886260504263249 [↑](#footnote-ref-11)
12. List revised from The Interdepartmental Child Abuse Committee (2011). *Provincial Child Abuse Protocol 2006*. Retrieved from <http://www.socialservices.gov.sk.ca/child-abuse-protocol.pdf> [↑](#footnote-ref-12)
13. Government of Saskatchewan (2012). Child Protection. Retrieved from <http://www.socialservices.gov.sk.ca/child-protection/> [↑](#footnote-ref-13)
