# Supplementary material for: A Randomised Controlled Trial of Therapist-Assisted, Internet-Delivered Cognitive Behavior Therapy for Women with Maternal Depression
Source: PLoS One. 2016 Mar 1;11(3):e0149186. doi: 10.1371/journal.pone.0149186 (PMC4773121; doi:10.1371/journal.pone.0149186)
Supplement: S1 Table — (DOC) [file pone.0149186.s005.doc]

**S1 Table Demographic Characteristics by Group**

| Characteristic | TA-ICBT (*N* = 24) Number (%) | WLC (*N* = 23) Number (%) |
| --- | --- | --- |
|  |  |  |
| Ethnicity |  |  |
| Caucasian | 22 (92) | 23 (100) |
| Other | 2 (8) | - |
| Relationship Status |  |  |
| Married/Common-Law | 22 (92) | 18 (78) |
| Engaged | 1 (4) | - |
| Dating | - | 1 (4) |
| Single | 1 (4) | 4 (17) |
| Education |  |  |
| < Grade 12 | 2 (8) | 1 (4) |
| High School Diploma; GED | 1 (4) | 4 (17) |
| College/ Some University | 7 (29) | 2 (9) |
| Undergraduate Degree | 9 (38) | 13 (57) |
| Graduate Degree(s) | 5 (21) | 3 (13) |
| Psychological Medication | 9 (38) | 5 (22) |
| Current Tobacco Use | 6 (25) | 2 (9) |
| Current Alcohol Use | 8 (33) | 15 (65) |
| Breast Feed | 19 (79) | 23 (100) |
| Vaginal Delivery | 20 (83) | 20 (87) |
| Twin Births | 1 (4) | 1 (4) |
| Complications with Delivery | 12 (50) | 9 (39) |
| Parity |  |  |
| One | 11 (46) | 11 (48) |
| Two | 8 (33) | 11 (48) |
| Three | 5 (21) | 1 (4) |
| Four | 1 (4) | - |
| Childcare Services | 10 (42) | 8 (32) |

*Note*. TA-ICBT = Therapist-Assisted Internet-Delivered Cognitive-Behavior Therapy; WLC= Waitlist Control; Parity = the number of times given birth
