# Supplementary material for: A Randomised Controlled Trial of Therapist-Assisted, Internet-Delivered Cognitive Behavior Therapy for Women with Maternal Depression
Source: PLoS One. 2016 Mar 1;11(3):e0149186. doi: 10.1371/journal.pone.0149186 (PMC4773121; doi:10.1371/journal.pone.0149186)
Supplement: S2 Table — (DOC) [file pone.0149186.s006.doc]

| **S2 Table Means and Standard Deviations for Primary and Secondary Outcomes** | | | | | | | | | |
| --- | --- | --- | --- | --- | --- | --- | --- | --- | --- |
|  | TA-ICBT | | | |  | WLC | | | |
|  | *M* (*SD*) | | | |  | *M* (*SD*) | | | |
| Outcome | Screening | T1 | T2 | T3 |  | Screening | T1 | T2 | T3 |
|  | *N* = 25 | *N* = 25 | *N* = 19 | *N* = 15 |  | *N* = 25 | *N* = 24 | *N* = 21 | *N* = - |
| EPDS | 15.68 (4.23) | 14.92 (4.32) | 8.68 (3.80) | 5.60 (2.35) |  | 16.24 (3.54) | 15.13 (4.06) | 12.71 (3.70) | - |
|  | T1 | | T2 | |  | T1 | | T2 | |
|  | *N* = 23-25 | | *N* = 19 | |  | *N* = 22-25 | | *N* = 20-21 | |
| DASS-Depression | 15.2 (7.14) | | 5.05 (5.67) | |  | 15.39 (9.07) | | 11.52 (8.39) | |
| DASS-Anxiety | 13.04 (8.49) | | 6.10 (6.16) | |  | 9.04 (8.22) | | 7.62 (6.74) | |
| DASS-Stress | 19.13 (7.37) | | 12.32 (6.26) | |  | 17.39 (7.44) | | 18.19 (5.79) | |
| PSI-CDI | 21.36 (7.41) | | 18.58 (5.98) | |  | 21.55 (7.51) | | 22.20 (6.73) | |
| PSI-PD | 40.92 (5.39) | | 31.79 (8.93) | |  | 38.22 (8.94) | | 36.40 (7.49) | |
| PSI-DC | 29.70 (8.93) | | 26.26 (6.89) | |  | 28.23 (9.13) | | 28.80 (8.82) | |
| WHOQOL-D1 | 3.20 (0.50) | | 4.43 (3.27) | |  | 3.30 (0.74) | | 3.06 (0.76) | |
| WHOQOL-D2 | 2.76 (0.50) | | 4.67 (2.92) | |  | 2.74 (0.78) | | 2.61 (0.95) | |
| WHOQOL-D3 | 2.67 (0.74) | | 4.33 (2.58) | |  | 2.97 (1.15) | | 2.71 (1.49) | |
| WHOQOL-D4 | 3.49 (0.54) | | 4.63 (3.01) | |  | 3.50 (1.15) | | 3.09 (1.63) | |
| *Note.* TA-ICBT = Therapist-assisted Internet-delivered Cognitive Behavior Therapy; WLC= Waitlist Control; T1 = Time one; T2 = Post-intervention/delay period; T3= 10-week follow-up; EPDS = Edinburgh Postnatal Depression Scale; DASS = Depression Anxiety Stress; PSI = Parenting Stress Index; CDI = Parent-Child Dysfunctional Interaction; PD = Parental Distress; DC = Difficult Child; WHOQOL = World Health Organization Quality of Life-BREF; D1 = Domain 1, Physical Health; D2= Domain 2, Psychological Health; D3 = Domain 3, Social Relationships; D4 = Domain 4, Environmental. | | | | | | | | | |
