# Supplementary material for: A Randomised Controlled Trial of Therapist-Assisted, Internet-Delivered Cognitive Behavior Therapy for Women with Maternal Depression
Source: PLoS One. 2016 Mar 1;11(3):e0149186. doi: 10.1371/journal.pone.0149186 (PMC4773121; doi:10.1371/journal.pone.0149186)
Supplement: S3 Table — (DOC) [file pone.0149186.s007.doc]

S3 Table Regression Analyses of Secondary Outcome Variables

| Variable | | *B* | *SE B* | Β | *R* |
| --- | --- | --- | --- | --- | --- |
| DASS-Stress T2 *F*(3, 38) = 4.23, *p* = .01, *R*^2^ = .25 | | | | | |
|  | *Constant* | 12.97 |  |  |  |
|  |  |  |  |  |  |
|  | *Group* | -5.72 | 2.36 | -.41^**^ | .40 |
|  | *DASS-Stress T1* | .28 | .13 | .31^*^ | .26 |
| DASS-Anxiety T 2 *F*(3, 33) = 9.23, *p* <. 001, *R*^2^ = .46 | | | | |  |
|  | *Constant* | 5.35 |  |  |  |
|  |  |  |  |  |  |
|  | *Group* | -2.64 | 2.0 | -.20 | .22 |
|  | *DASS-Anx T1* | .59 | .12 | .65^**^ | .59 |
| DASS-Depression T 2 *F*(3, 38) = 6.16, *p* = .002, *R*^2^ = .33 | | | | |  |
|  | *Constant* | 4.19 |  |  |  |
|  |  |  |  |  |  |
|  | *Group* | -4.27 | 2.7 | -.25 | .31 |
|  | *DASS-Dep T1* | .54 | .15 | .49^**^ | .50 |
| PSI-Total T2 *F*(3, 35) = 19.86, *p* < .001, *R*^2^ = .63 | | | | |  |
|  | *Constant* | 23.00 |  |  |  |
|  |  |  |  |  |  |
|  | *Group* | -8.85 | 4.46 | -.25a | .24 |
|  | *PSI-Tot T1* | .73 | .10 | .75^**^ | .75 |
| PSI-PD T2 *F*(3, 37) = 16.24, *p* < .001, *R*^2^ = .57 | | | | |  |
|  | *Constant* | 7.72 | 5.53 |  |  |
|  |  |  |  |  |  |
|  | *Group* | -6.46 | 2.11 | -.41^**^ | .26 |
|  | *PSI-PD T1* | .77 | .12 | .72^**^ | .63 |
| PSI-CDI T2 *F*(3, 37) = 10.55, *p* < .001, *R*^2^ = .46 | | | | |  |
|  | *Constant* | 11.55 | 3.75 |  |  |
|  |  |  |  |  |  |
|  | *Group* | .61 | -1.84 | -.05 | .19 |
|  | *PSI-CDI T1* | .56 | .11 | .62^**^ | .65 |
| PSI-DC T2 *F*(3, 35) = 14.15, *p*< .001, *R*^2^ = .55 | | | | |  |
|  | *Constant* | 7.83 |  |  |  |
|  |  |  |  |  |  |
|  | *Group* | -3.09 | 2.2 | -.19 | .14 |
|  | *PSI-DC T1* | .67 | .11 | .74^**^ | .72 |
| WHO-D1 T2 *F*(3, 37) = 5.25, *p* < .01, *R*^2^ = .30 | | | | |  |
|  | *Constant* | 2.06 |  |  |  |
|  |  |  |  |  |  |
|  | *Group* | .22 | .15 | .24 | -.13 |
|  | *WHO-D1 T1* | .32 | .13 | .39^**^ | .18 |
| WHO-D2 T2 *F*(3, 36) = 8.79, *p* < .001, *R*^2^ = .49 | | | | |  |
|  | Constant | .62 |  |  |  |
|  |  |  |  |  |  |
|  | Group | .42 | .18 | .34^*^ | -.14 |
|  | WHO-D2 T1 | .72 | .15 | .61^**^ | .33 |
| WHO-D3 T2 *F*(3, 37) = 6.57, *p* < .001, *R*^2^ = .35 | | | | |  |
|  | Constant | 1.62 |  |  |  |
|  |  |  |  |  |  |
|  | Group | .32 | .23 | .24 | .08 |
|  | WHO-D3 T1 | .57 | .13 | .65^**^ | .28 |
| WHO-D4 T2 *F*(3, 37) = 17.63, *p* < .001, *R*^2^ = .59 | | | | |  |
|  | Constant | 1.13 |  |  |  |
|  |  |  |  |  |  |
|  | Group | .33 | .14 | .31^*^ | .04 |
|  | WHO-D4 T1 | .79 | .11 | .79^**^ | .39 |

*Note*. DASS = Depression Anxiety Stress; PSI-CDI-= Parenting Stress Index-Parent-Child Dysfunctional Interaction; PSI-PD = Parenting Stress Index- Parental Distress; PSI-DC = Parenting Stress Index-Difficult Child; WHO-QOL = World Health Organization Quality of Life-BREF; WHO-QOL-1 = Physical Health; WHO-QOL-2= Psychological Health; WHO-QOL-3 = Social Relationships; WHO-QOL-4 = Environmental.
*a p < .10 * p* < .05, ** p < .01
