# Supplementary material for: A Randomised Controlled Trial of Therapist-Assisted, Internet-Delivered Cognitive Behavior Therapy for Women with Maternal Depression
Source: PLoS One. 2016 Mar 1;11(3):e0149186. doi: 10.1371/journal.pone.0149186 (PMC4773121; doi:10.1371/journal.pone.0149186)
Supplement: S4 Table — (DOC) [file pone.0149186.s008.doc]

**S4 Table Descriptive Statistics for Program Engagement in the TA-ICBT condition (*N*=24)**

|  | M (*SD*) | Min | Max |
| --- | --- | --- | --- |
|  |  |  |  |
| Number of days of program access | 69.04 (30.10) | 8 | 129 |
| Number of program visits | 26.88 (11.63) | 7 | 56 |
| Number of modules completed | 5.92 (1.52) | 2 | 7 |
| Emails sent from client | 5.4 (4.15) | 0 | 19 |
| Emails sent from therapist | 10.52 (3.95) | 3 | 20 |
|  |  |  |  |
